# Supplementary material for: The C-terminal SUMOylation-dependent regulation of αKNL2 governs its centromere targeting and interaction with CENH3
Source: Plant Commun. 2025 Nov 19;7(2):101617. doi: 10.1016/j.xplc.2025.101617 (PMC12903445; doi:10.1016/j.xplc.2025.101617)
Supplement: Document S1. Supplemental Figures 1–12 and Supplemental Table 1 [file mmc1.pdf]

Plant Communications, Volume 7

Supplemental information

The C-terminal SUMOylation-dependent regulation of αKNL2 governs its centromere targeting and interaction with CENH3

Manikandan Kalidass, Jitka Vaculíková, Jothipriya Ramakrishnan Chandra, Barbora Králová, Venkata Ganesh Jarubula, Sevim D. Kara Öztürk, Dmitri Demidov, Veit Schubert, David Potesil, Jan J. Palecek, and Inna Lermontova

Supplemental information

The C-terminal SUMOylation-dependent regulation of αKNL2 governs its centromere targeting and interaction with CENH3

Manikandan Kalidass[1*], Jitka Vaculíková[2], Jothipriya Ramakrishnan Chandra[1], Barbora Králová[2], Venkata Ganesh Jarubula[1,3], Sevim D. Kara Öztürk[4], Dmitri Demidov[1], Veit Schubert[1], David Potesil[5], Jan J. Palecek[2,5], Inna Lermontova[1*]

[1]Leibniz Institute of Plant Genetics and Crop Plant Research (IPK) Gatersleben, Corrensstrasse 3, D-06466 Seeland, Germany

[2]National Centre for Biomolecular Research, Faculty of Science, Masaryk University, Kamenice 5, 62500 Brno, Czech Republic

[3]Department of Program Center Metacom, Leibniz Institute of Plant Biochemistry, Weinberg 3, D-06120 Halle (Saale), Germany

[4]Department of Agricultural Genetic Engineering, Ayhan Şahenk Faculty of Agricultural Sciences and Technologies, Niğde Ömer Halisdemir University, 51240, Niğde, Türkiye

[5]Central European Institute of Technology (CEITEC), Masaryk University, Kamenice 5, 62500 Brno, Czech Republic

*Authors for correspondence: kalidass@ipk-gatersleben.de; lermonto@ipk.gatersleben.de

The supplemental file includes 12 Supplemental Figures and 1 Supplemental Table

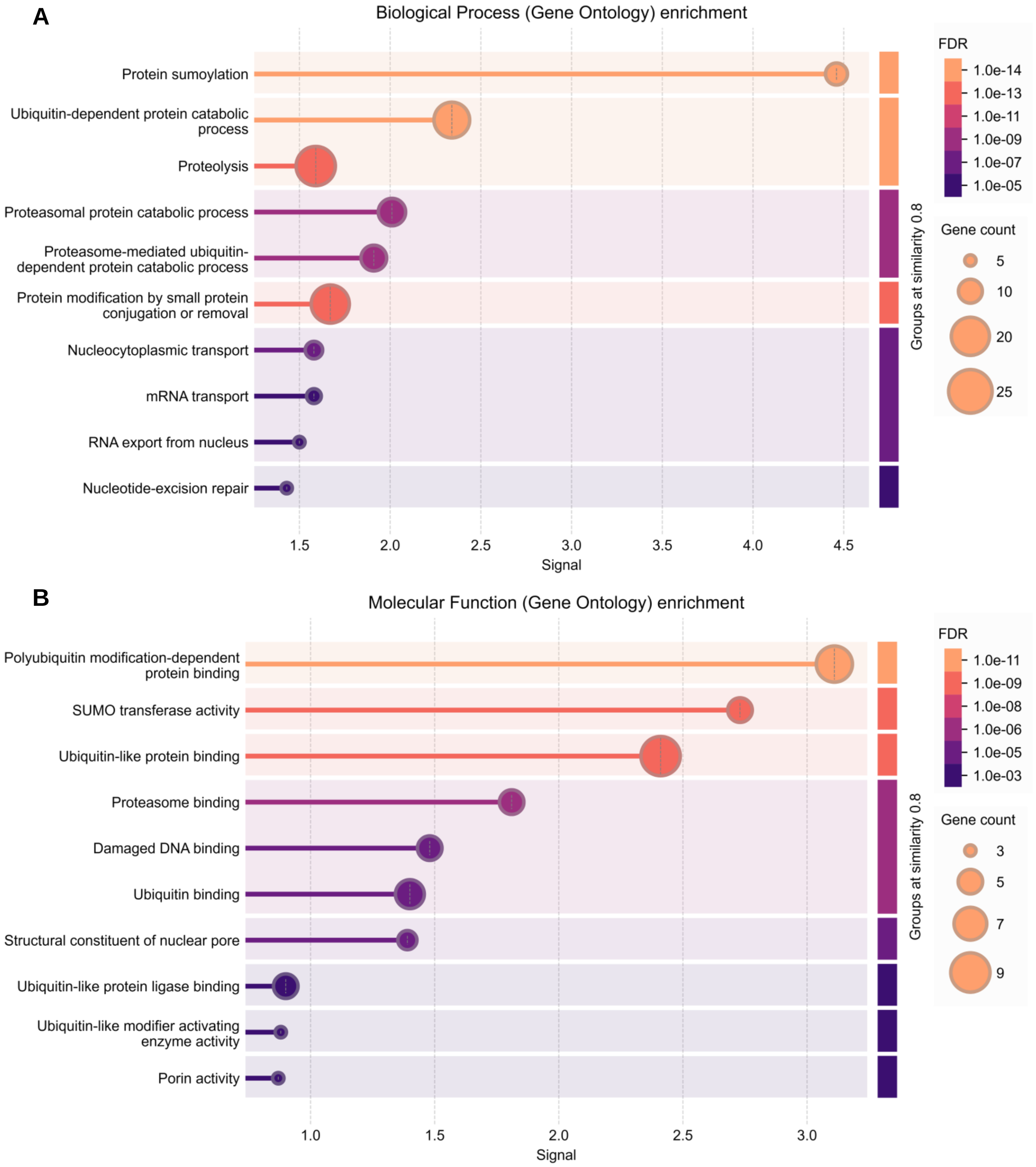

Supplementary Figure 1. The post-translational modification pathway analysis of αKNL2 interactors based on Y2H screening

(A, B) The gene ontology analysis such as biological process **(A)** and molecular function **(B)** of post-translational modification of αKNL2 interactors identified proteolysis, SUMOylation and transport pathways.

A

		VENUS-C							
		SUMO1	SUMO2	SUMO3	SUMO5	ULP1d	ULP1d-C	ULP1d-N	Empty
VENUS-N	αKNL2								
	αKNL2-C			Nucleolus		Nucleolus	Nucleolus		
	αKNL2-N								
	Empty								

Positive interaction   No interaction

B

		VENUS-C			
		αKNL2	αKNL2-C	αKNL2-N	Empty
VENUS-N	SUMO1				
	SUMO2				
	SUMO3		Nucleolus		
	SUMO5				
	ULP1d		Nucleolus		
	ULP1d-C		Nucleolus		
	ULP1d-N				
	Empty				

Positive interaction   No interaction

Supplementary Figure 2. The interaction analysis of SUMO and ULP1d proteins with αKNL2 by BiFC

The BiFC interactions shown for the combinations such as αKNL2, αKNL2-N, αKNL2-C fused to VENn and SUMO1, SUMO2, SUMO3, SUMO5, ULP1d, ULP1d-N or ULP1d-C fused to VENc **(A)** and vice versa **(B)**. SUMO3 and ULP1d showed interaction only with αKNL2-C, while other SUMO proteins did not interact with any αKNL2 variants. The empty BiFC negative controls were used to validate the positive interactions.

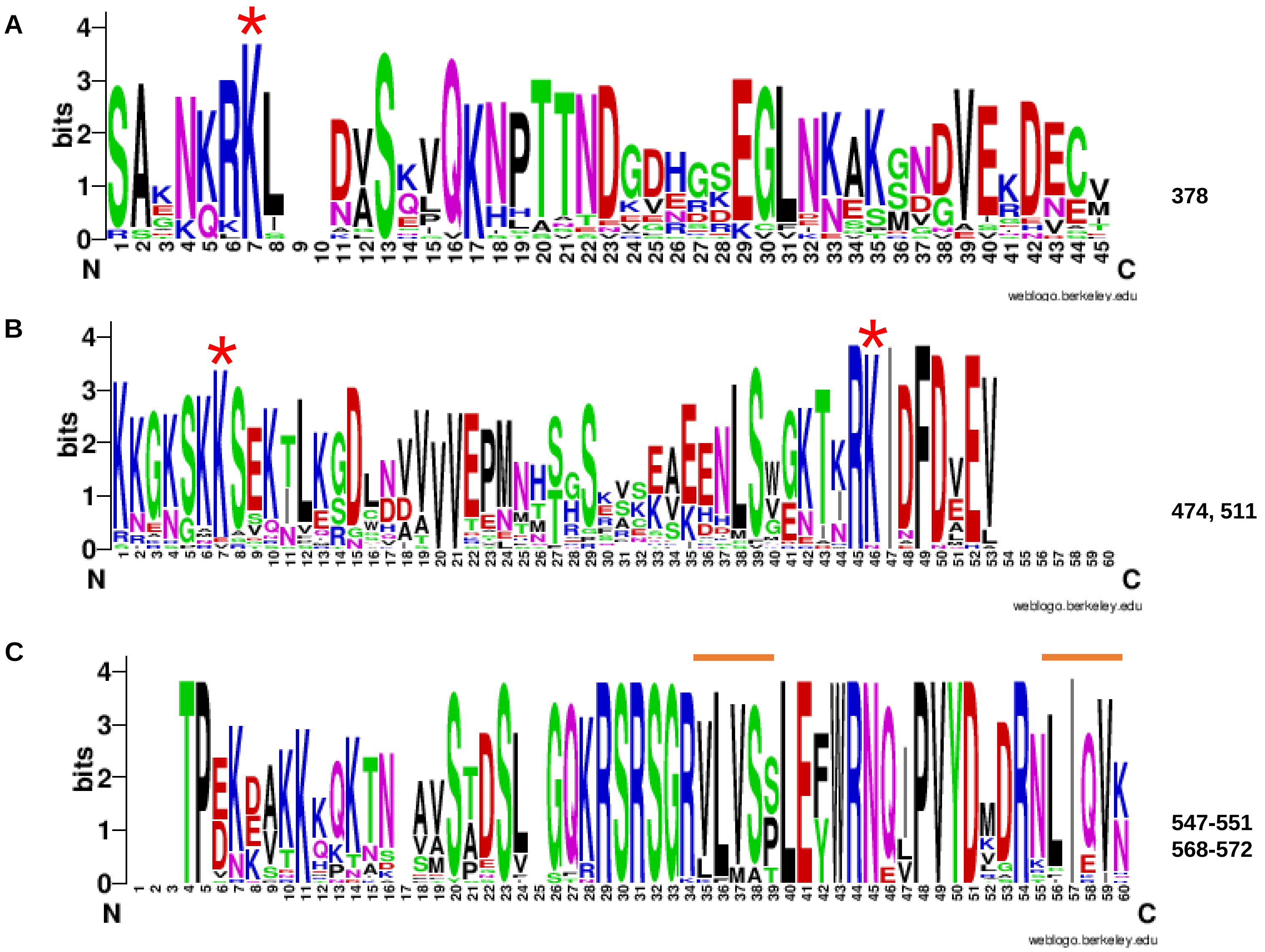

Supplementary Figure 3. The conservation analysis of SUMOylation and SIM sites in αKNL2-C

(A–C) The conservation of SUMOylation and SIM sites predicted by GPS-SUMO in αKNL2-C across Brassicales species, as illustrated using WebLogo (https://weblogo.berkeley.edu/logo.cgi). Conserved lysine residues and SUMO interaction sites are indicated by red asterisks and orange lines, respectively.

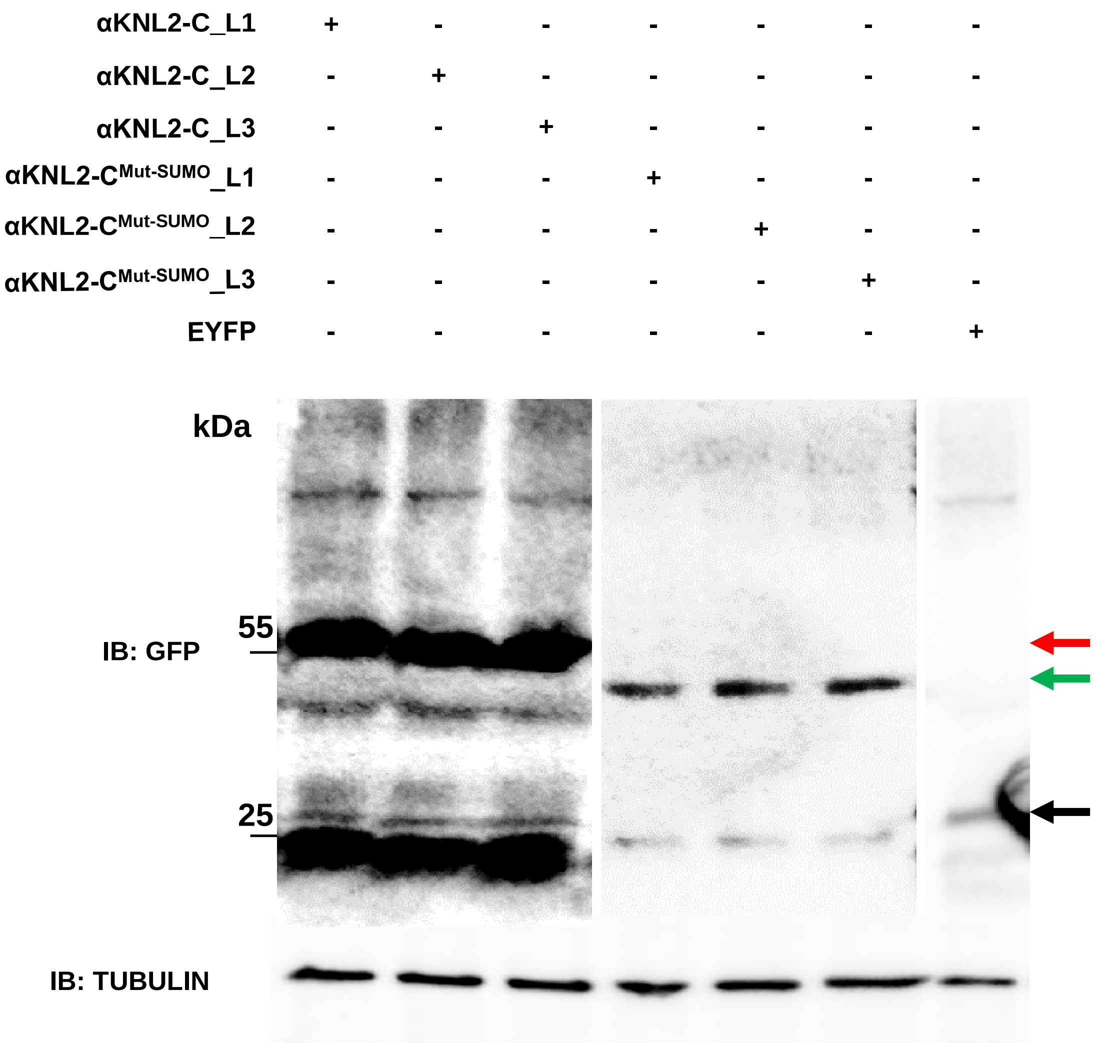

Supplementary Figure 4. Immunoblot detection of αKNL2-C-EYFP and SUMOylation-deficient αKNL2-C$^{Mut-SUMO}$-EYFP in Arabidopsis transgenic lines

Total protein extracts from three independent transgenic lines expressing αKNL2-C-EYFP, SUMOylation-deficient mutant αKNL2-C$^{Mut-SUMO}$-EYFP or EYFP alone were subjected to GFP immunoblotting. The red arrow, green and black indicates the expected size of the αKNL2-C-EYFP, αKNL2-C$^{Mut-SUMO}$-EYFP and EYFP fusion protein, respectively. The tubulin was used as a loading control to confirm equal protein loading. IB, Immunoblot.

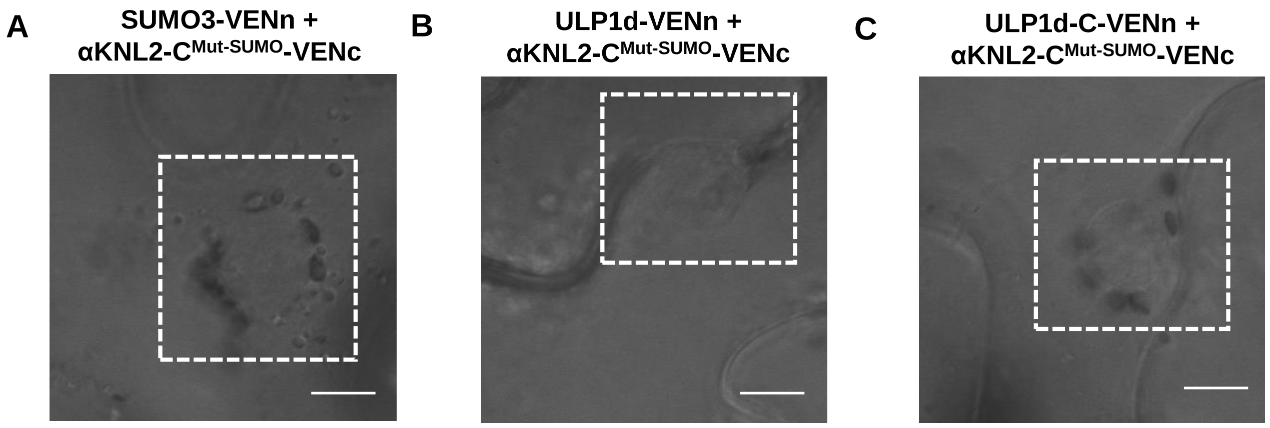

D

		VENUS-C		
	Proteins	SUMO3	ULP1d	ULP1d-C
VENUS-N	αKNL2			
	αKNL2-C	Nucleolus	Nucleolus	Nucleolus
	αKNL2-N			
	αKNL2-C$^{Mut\text{-}SUMO}$			

Positive interaction — No interaction — No interaction after mutagenesis

E

		VENUS-C		
	Proteins	SUMO3	ULP1d	ULP1d-C
VENUS-N	K378R--αKNL2-C	Nucleolus	Nucleolus	Nucleolus
	K474R--αKNL2-C	Nucleolus	Nucleolus	Nucleolus
	K511R--αKNL2-C	Nucleolus	Nucleolus	Nucleolus
	Δ547-551--αKNL2-C	Nucleolus	Nucleolus	Nucleolus
	Δ568-572--αKNL2-C	Nucleolus	Nucleolus	Nucleolus
	K378R-K474R--αKNL2-C	Nucleolus	Nucleolus	Nucleolus
	K474R-K511R--αKNL2-C			

Positive interaction — No interaction — No interaction after mutagenesis

Supplementary Figure 5. The interaction of SUMO3 and ULP1d with SUMO mutant variants of αKNL2-C by BiFC

(A-C) No interaction was found between SUMO3, ULP1d, ULP1d-C fused to VENn with αKNL2-C$^{Mut\text{-}SUMO}$ fused to VENc. The nucleus lacking Venus fluorescence is indicated by white dotted circles. Scale bars represents 5 µm. **(D)** The similar interaction results was found when the orientation of the Venus fusion was reversed. The interaction was indicated in a colour code below the table. **(E)** Analysis of the interactions between individual SUMOylation and SIM sites of αKNL2-C with SUMO3, ULP1d, or ULP1d-C. Double lysine mutations (K474R and K511R) abolished interactions with SUMO3 and ULP1d. Interaction outcomes are indicated by a color code below the table.

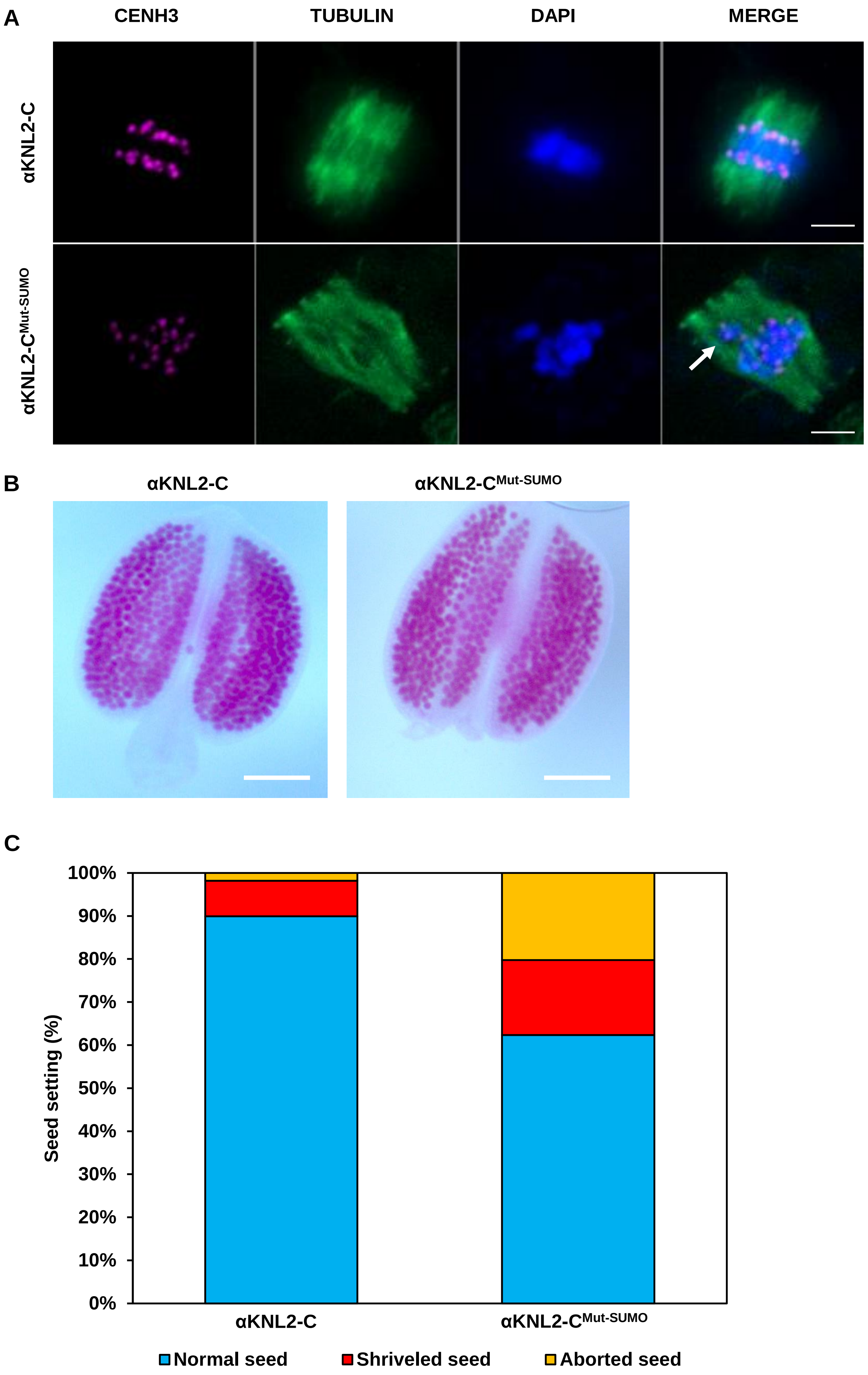

Supplementary Figure 6. Analysis of chromosome segregation defects, pollen viability, and seed set in the SUMOylation-deficient αKNL2 mutant

(A) Representative images of mitotic chromosome segregation in Arabidopsis αKNL2 SUMO mutant plants. Microtubules (green) are stained with anti-tubulin, centromeres (magenta) with anti-CENH3, and DNA (blue) with DAPI. The upper row shows normal metaphase alignment, while the lower panel depicts a misaligned chromosome (arrow) during metaphase. Scale bars 5 μm. **(B)** Alexander staining of pollen grains from αKNL2-C (left) and αKNL2$^{\text{Mut-SUMO}}$ mutant (right) plants. The mutant anthers did not show any difference compared to αKNL2-C control plants, suggesting no defects in pollen viability. Scale bars 10 μm. **(C)** Analysis of seed setting in *Arabidopsis* αKNL2-C or αKNL2-C$^{\text{Mut-SUMO}}$ mutant variant. Bar graph showing the number of normal, shriveled and aborted seeds per silique for 10 plants per construct, with 10 siliques analyzed per plant.

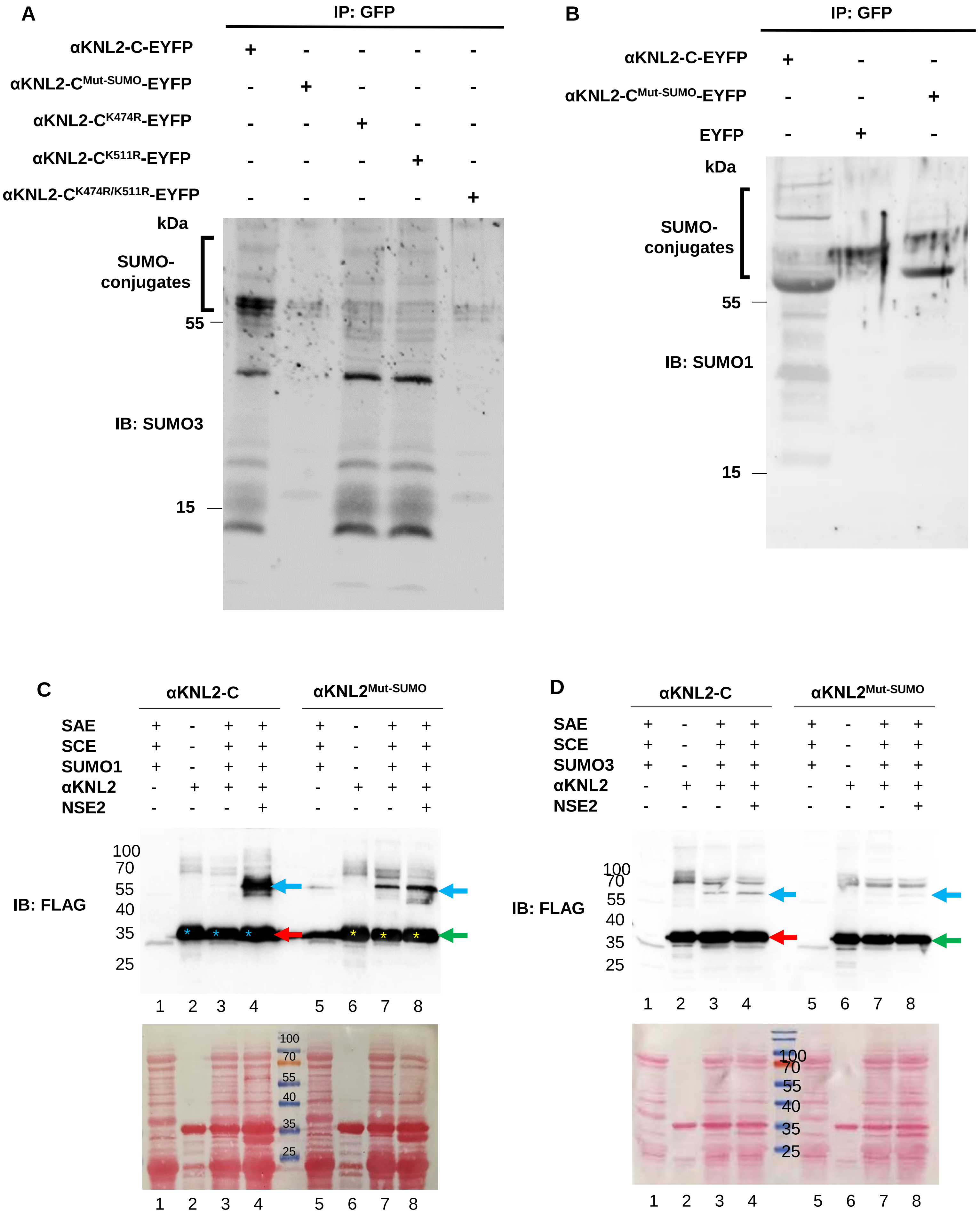
A
IP: GFP
αKNL2-C-EYFP
αKNL2-C$^{Mut-SUMO}$-EYFP
αKNL2-C^{K474R}-EYFP
αKNL2-C^{K511R}-EYFP
αKNL2-C$^{K474R/K511R}$-EYFP
kDa
SUMO-conjugates
55
15
IB: SUMO3
B
IP: GFP
αKNL2-C-EYFP
αKNL2-C$^{Mut-SUMO}$-EYFP
EYFP
kDa
SUMO-conjugates
55
15
IB: SUMO1
C
αKNL2-C
αKNL2$^{Mut-SUMO}$
SAE
SCE
SUMO1
αKNL2
NSE2
IB: FLAG
100
70
55
40
35
25
1 2 3 4 5 6 7 8
D
αKNL2-C
αKNL2$^{Mut-SUMO}$
SAE
SCE
SUMO3
αKNL2
NSE2
IB: FLAG
100
70
55
40
35
25
1 2 3 4 5 6 7 8

Supplementary Figure 7. The in vivo and in vitro SUMOylation analysis of αKNL2-C

(A, B) In vivo SUMOylation of αKNL2 by SUMO3 or SUMO1 in *Nicotiana benthamiana*. **(A)** Leaves expressing αKNL2-C-EYFP, αKNL2-C$^{Mut\text{-}SUMO}$-EYFP, αKNL2-C^{K474R}-EYFP, αKNL2-C^{K511R}-EYFP, or αKNL2-C$^{K474R/K511R}$-EYFP were tested with SUMO3. **(B)** Leaves expressing αKNL2-C-EYFP, αKNL2-C$^{Mut\text{-}SUMO}$-EYFP, or EYFP alone were tested with SUMO1. Total protein extracts were immunoprecipitated with GFP-Trap beads and probed with anti-SUMO3 or anti-SUMO1 antibodies. SUMO conjugates are marked by black brackets. IB, immunoblot; IP, immunoprecipitation. **(C, D)** The in vitro SUMOylation assay was performed to assess the SUMOylation efficiency of αKNL2-C and its SUMO mutant variant using the SUMO1 (**C**) or SUMO3 (**D**) isoforms. The reactions included enzymes only (lanes 1 and 5), substrate only (lanes 2 and 6), a mixture of enzymes and substrate (lanes 3 and 7), and a complete reaction with the addition of NSE2 SUMO-E3 ligase (lanes 4 and 8). Following incubation, samples were analyzed using SDS-PAGE and immunoblotting. The membrane was reversibly stained with Ponceau S red (lower panels) as a loading control to verify equal protein loading across all reactions. Both αKNL2 variants and their SUMOylated forms were detected using an anti-FLAG antibody (upper panels). The red arrows represents the unmodified αKNL2-C, the green arrows marks the unmodified αKNL2-C$^{Mut\text{-}SUMO}$ mutant, and the blue arrows indicate SUMOylated forms. The SUMO mutant shows a significantly reduced SUMOylation efficiency compared to the wild-type variant. Notably, the addition of NSE2 did not enhance SUMO3 efficiency for either αKNL2 variant, while it enhanced SUMO1 efficiency for both variants. IB, immunoblot; IP, immunoprecipitation.

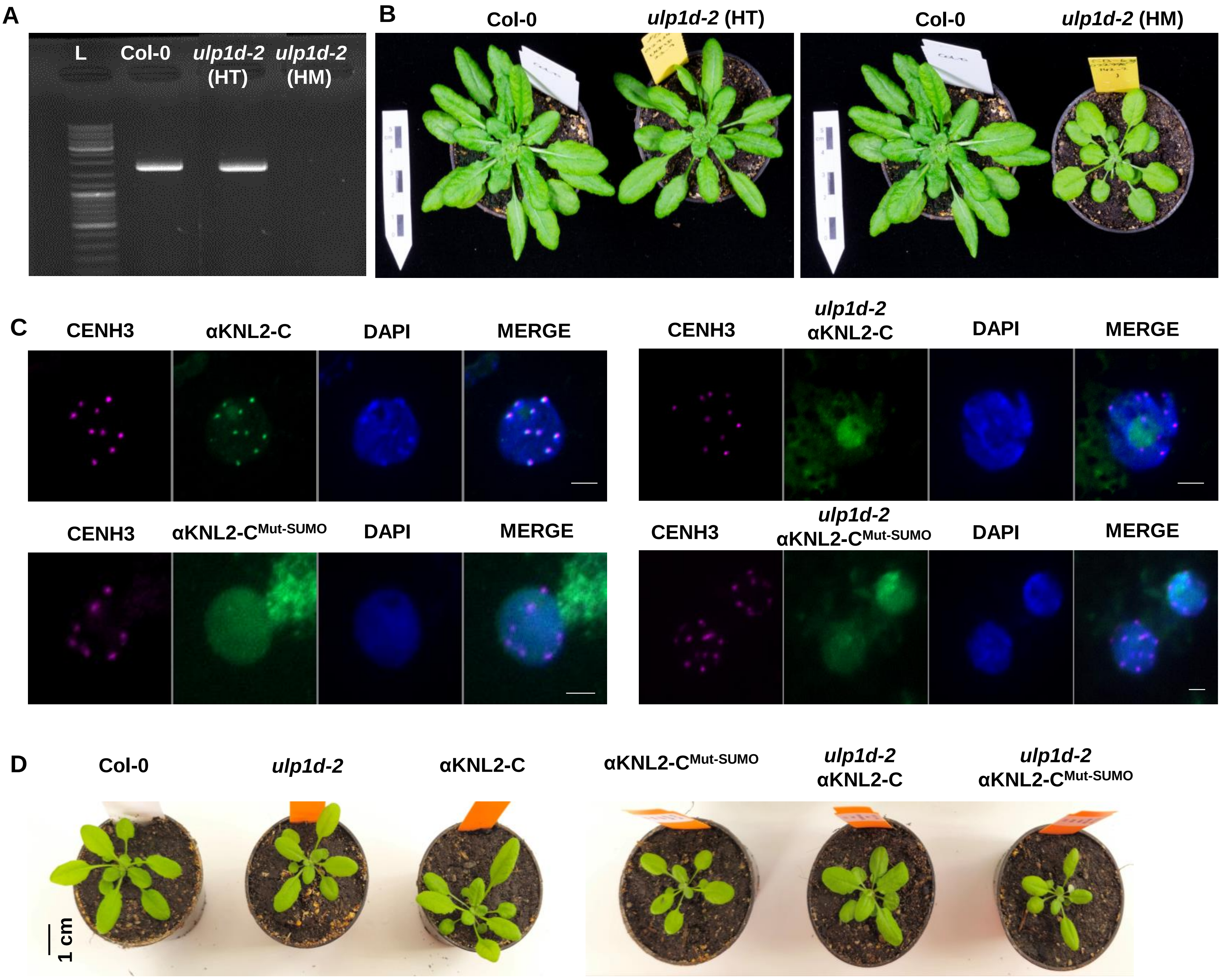

Supplementary Figure 8. The phenotype characteristics and localization of αKNL2-C and αKNL2-C$^{Mut\text{-}SUMO}$ in *ulp1d-2*
(A-B) RT-PCR analysis of ULP1d expression (A) and the phenotype of 5 weeks grown plants (B) of wild-type (Col-0), *ulp1d-2* heterozygous and homozygous backgrounds. **(C)** Immunostaining experiments showing the co-localization of αKNL2-C and KNL2-C$^{Mut\text{-}SUMO}$ (green) in meristematic nuclei of wild-type and *ulp1d-2* mutants. The nuclei were stained with anti-CENH3 (red) and DAPI was used as a counterstain. Scale bars represents 5 µm. **(D)** The phenotype of the αKNL2-C and KNL2-C$^{Mut\text{-}SUMO}$ in wild-type and *ulp1d-2* mutants grown for 4 weeks in soil. Scale bar represents 1 cm.

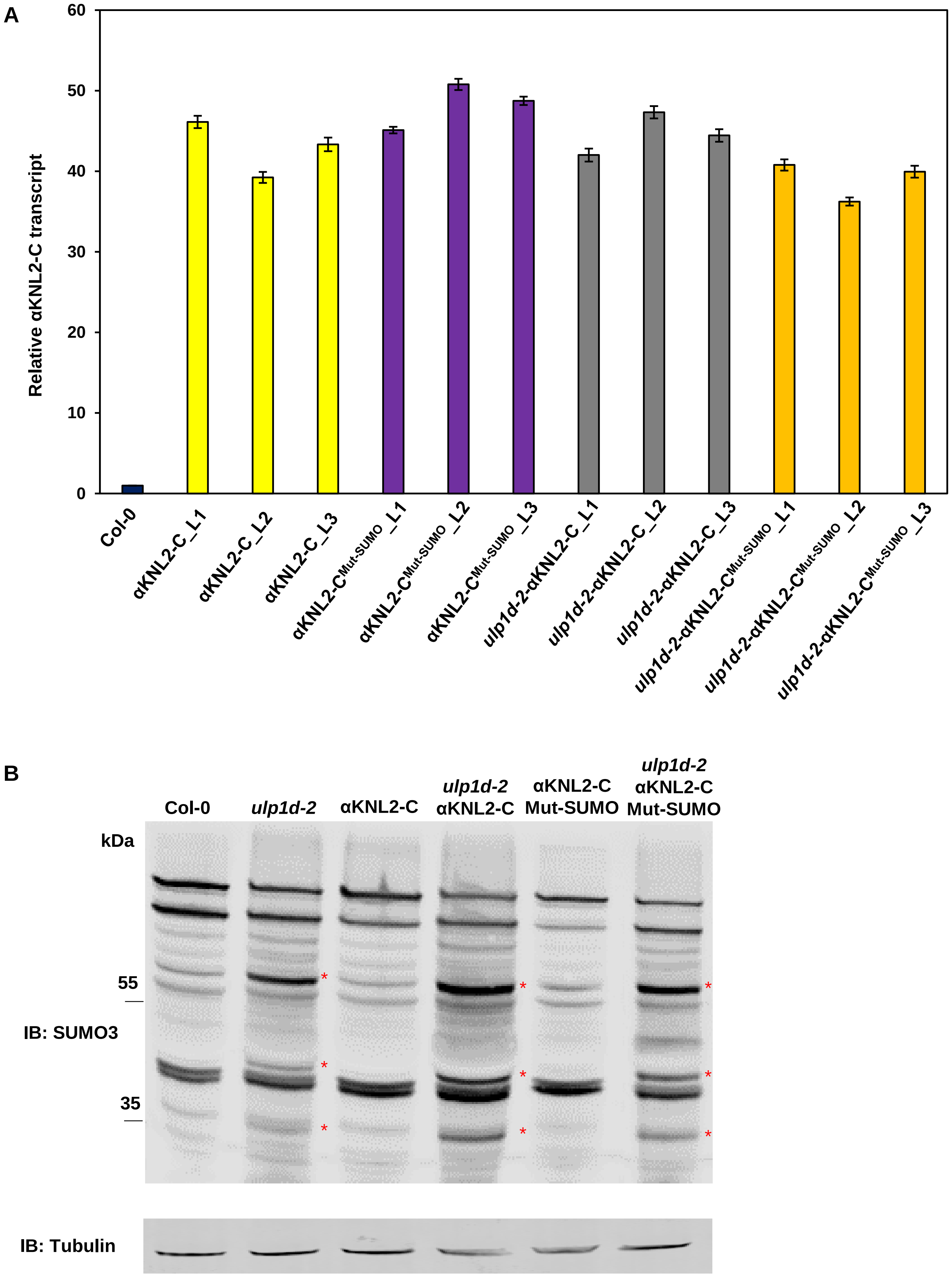

Supplementary Figure 9. Transcript levels of *αKNL2* and SUMO3 western blot in αKNL2-C and αKNL2-C$^{Mut\text{-}SUMO}$ lines in wild-type and *ulp1d-2* mutant plants

(A) Quantitative real-time PCR (RT-qPCR) analysis of αKNL2 transcripts in αKNL2-C and SUMOylation-deficient αKNL2-C$^{Mut\text{-}SUMO}$ independent Arabidopsis transgenic lines. Wild-type (Col-0) were included as controls. Transcript levels were normalized to *ACTIN2 and UBQ* expression. Similar transcript levels were detected across αKNL2-C and αKNL2-C$^{Mut\text{-}SUMO}$ transgenic lines in Col-0 and *ulp1d-2.* Data represent as mean ± SEM. Statistical analysis by ANOVA revealed no significant differences between the lines ($p > 0.5$). **(B)** Western blot analysis against anti-SUMO3 in the total protein extracts from wild-type, *ulp1d-2,* αKNL2-C, and αKNL2-C$^{Mut\text{-}SUMO}$ in wild-type and *ulp1d-2* mutants. The red asterisks shows the increase in the band intensities in *ulp1d-2* mutant background compared to wild-type (Col-0). IB, Immunoblot.

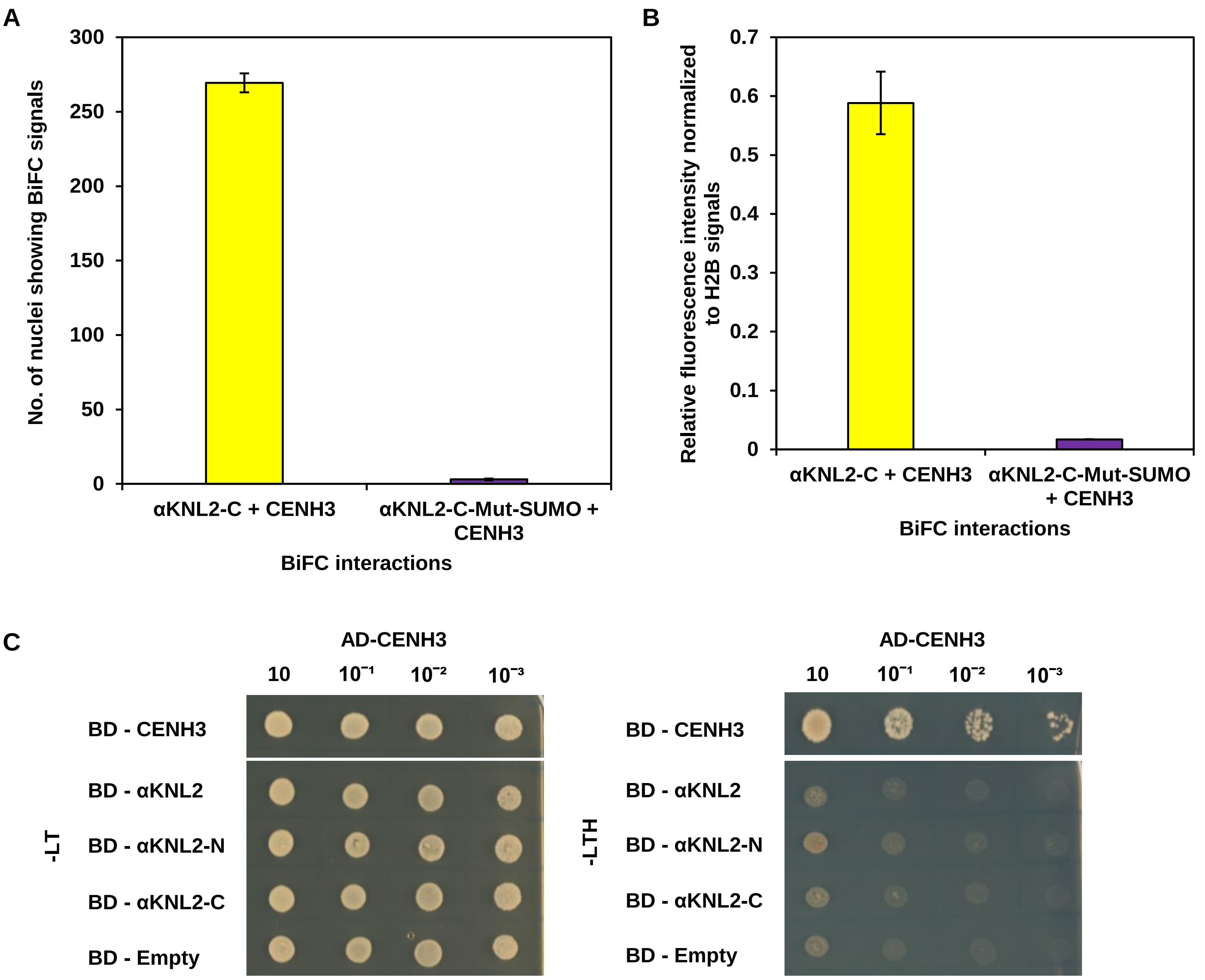

Supplemental Figure 10. BiFC quantification and yeast two-hybrid assay for interactions between αKNL2 and CENH3 **(A)** Bar graphs represent the number of nuclei showing BiFC signals for αKNL2, αKNL2$^{Mut\text{-}SUMO}$ and CENH3 interactions. The number of nuclei showing BiFC signals was measured in 80mm^2 area. Data are presented as mean ± SEM. **(B)** The fluorescence intensity for BiFC signals were measured after normalization with H2B signals from 30 nuclei per sample (n = 30). Data are presented as mean ± SEM. **(C)** Zygotes expressing both prey CENH3 and bait (CENH3, αKNL2, αKNL2-N, αKNL2-C) are selected on -LT (Double dropout: YNB without Leu and Trp). Protein-protein interactions are assessed on -LTH (Triple dropout: YNB without Leu, Trp, and His). The strength of the protein-protein interactions was evaluated by a drop dilution test. AD, activating domain; BD, binding domain.

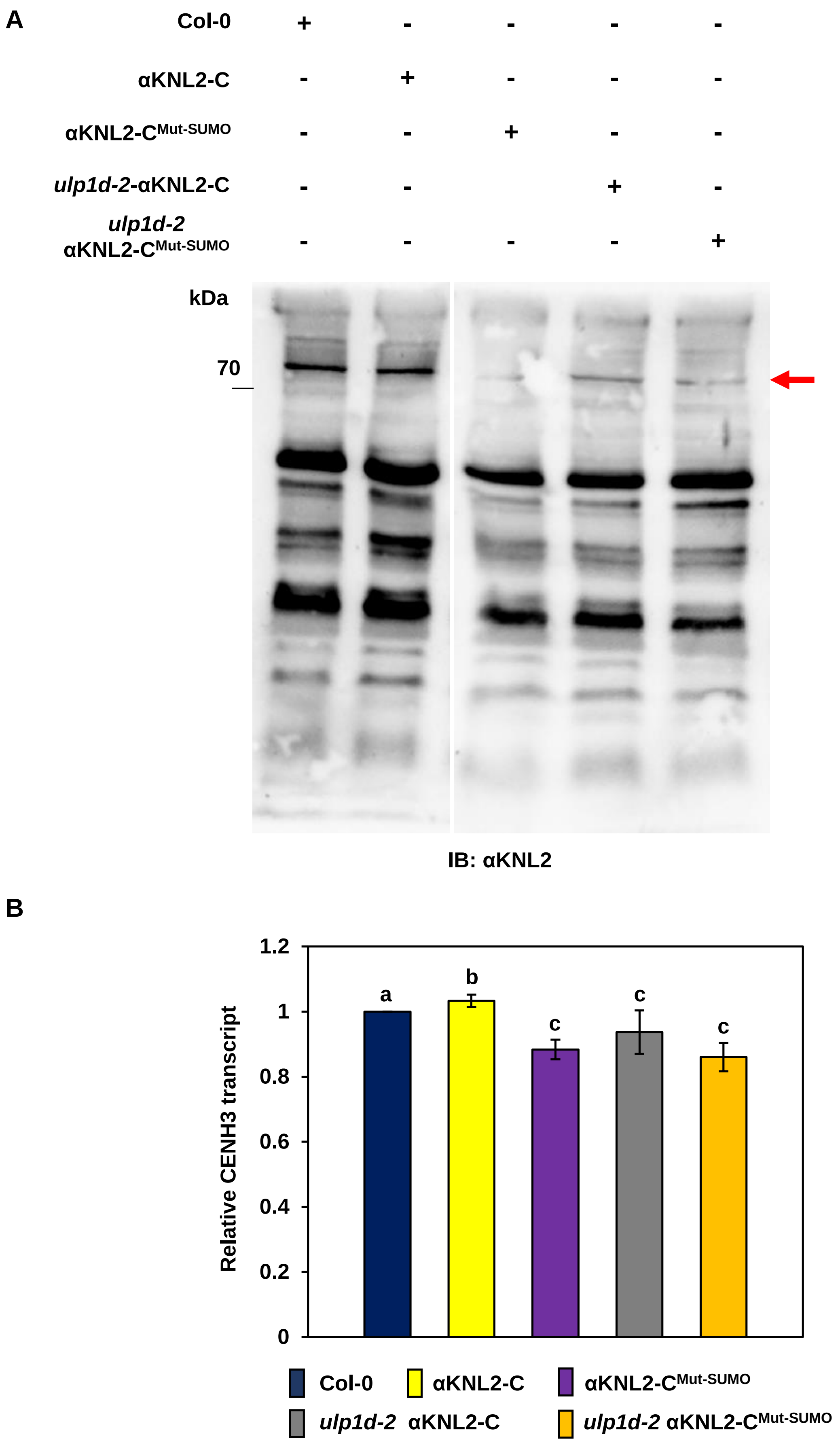

Supplementary Figure 11. αKNL2 protein and *CENH3* transcript levels in αKNL2-C and αKNL2-C$^{Mut\text{-}SUMO}$ lines in wild-type and *ulp1d-2* mutant plants

(A) Immunoblot analysis of αKNL2 in wild-type (Col-0), αKNL2-C-EYFP, and αKNL2-C$^{Mut\text{-}SUMO}$-EYFP in Col-0 and *ulp1d-2* background lines. The nuclear protein extracts were separated by SDS-PAGE and probed with anti-αKNL2 antibodies to detect endogenous αKNL2. The red arrow indicates the αKNL2 specific band. The tubulin control is same as Fig. 7C. **(B)** Quantification of *CENH3* transcript levels by RT-qPCR. Data are normalized to *ACTIN2, UBQ* expression and shown as mean ± SEM from three biological replicates. Significant differences are marked by lowercase letters based on ANOVA and Tukey's multiple comparison tests ($P < 0.005$).

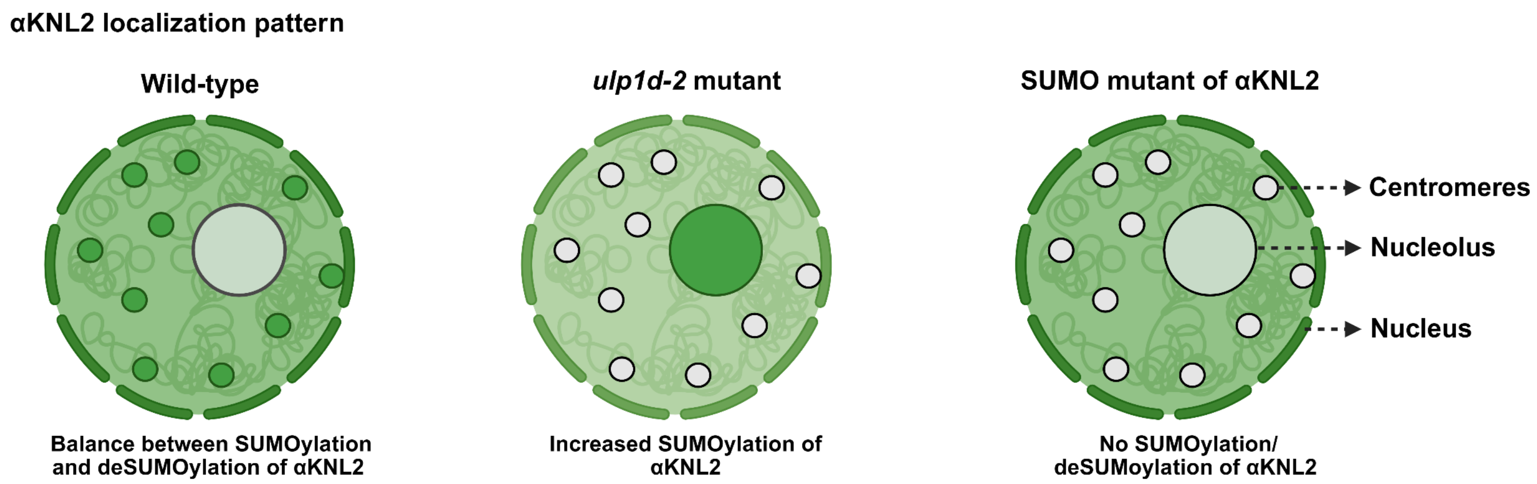

Supplementary Figure 12. SUMOylation-dependent regulation of αKNL2 localization and its impact on centromere function

In wild-type cells, αKNL2 (green) predominantly localizes to the centromere region and facilitates proper CENH3 deposition. In *ulp1d* mutants, αKNL2 localization is restricted to the nucleolus and is absent from the centromeres. In SUMOylation-deficient αKNL2 mutants, αKNL2-C fails to localize correctly and is entirely mislocalized to the cytoplasm. Nucleoplasmic signals were observed across all cases, with occasional weak nucleolar staining detected in both wild-type and SUMO mutant backgrounds.

Supplementary Table 1. Primers used in this study

S. No	Gene	Forward primer	Reverse primer
Amplification of genes using attB primers			
1	SUMO1 (AT4G26840)	GGGGACAAGTTTGTACAAAAAAGCAGGCTTCATGTCTGCAAACCAGGAGG	GGGGACCACTTTGTACAAGAAAGCTGGGTGGCCGTAGCACCACCACCGC
2	SUMO2 (AT5G55160)	GGGGACAAGTTTGTACAAAAAAGCAGGCTTCATGTCTGCTACTCCGGAAGA	GGGGACCACTTTGTACAAGAAAGCTGGGTAAAGCAGAAGAGCTTCAGGC
3	SUMO3 (AT5G55170)	GGGGACAAGTTTGTACAAAAAAGCAGGCTTCATGTCTAACCCTCAAGATGA	GGGGACCACTTTGTACAAGAAAGCTGGGTAAGCCCATTATGATCGAAAAG
4	SUMO5 (AT2G32765)	GGGGACAAGTTTGTACAAAAAAGCAGGCTTCATGGTGAGTTCCACAGACAC	GGGGACCACTTTGTACAAGAAAGCTGGGTAGGAGTGTAAGGACCGCCACC
5	ULP1d (AT1G60220)	GGGGACAAGTTTGTACAAAAAAGCAGGCTTCATGACGAAGAGGAAGAAGG	GGGGACCACTTTGTACAAGAAAGCTGGGTTTACTCTGTCTGGTCACTGAC
6	ULP1d-N (AT1G60220)	GGGGACAAGTTTGTACAAAAAAGCAGGCTTCATGACGAAGAGGAAGAAGG	GGGGACCACTTTGTACAAGAAAGCTGGGTCTTACGGCGCCTTGAACTTTG
7	ULP1d-C (AT1G60220)	GGGGACAAGTTTGTACAAAAAAGCAGGCTTCATGAAATCAGAGGACACAGTG	GGGGACCACTTTGTACAAGAAAGCTGGGTCTCTGTCTGGTCACTGACACG
Primers used to confirm the positive entry and destination clones			
8	attB1	GGGGACAAGTTTGTACAAAAAAGCAGGCTTC	
9	attB2	GGGGACCACTTTGTACAAGAAAGCTGGGTC	
Primers used for PCR-based site-directed mutagenesis			
10	K378R-αKNL2C	AAACAAAAGGAGAATCGATGCGAG	TCCGCACTTTTGACTTTCGTCCCAG
11	K474R-αKNL2C	GAAAATCAAAGAGAAGTGAGAAGA	CTTTCGACAGGGGATCTTGAAATGC
12	K511R-αKNL2C	AATAAAGAGGAGAATCGACTTTG	TTTTCCCATGACAAGTTTTCTTCAG
13	Δ547-551-αKNL2C	CTAGAGTTTTGGCGTAACCAAATTC	CCTTCCTGATCTTGACCGTTTCTGT
14	Δ568-572-αKNL2C	GATGGTAGTGAGACTAACTCCGCTC	GTTCCGATCCATATCATAAACAGG
Primers used for cloning constructs for in vitro SUMOylation assay			
15	αKNL2 C-terminus (WT and SUMO mutant) to pET-Duet ; JJ225, JJ226	ACCATCATCACCACAGCCAGATGAATTACTCTGGGACG	CTGAAAATACAGGTTTTCCGCTTTGATTTTCAAGTTTCTTCG
16	NSE2 to pET28 c+ ; JJ200, JJ201	GGTGGACAGCAAATGGGTCGGATCCCCATGGCGTCGGCGTCCTCG	GGTGGTGGTGGTGGTGCTCGAGCTAATCTTCATCCACATCTTCTGTGAA
**Primers used for RT-qPCR analysis**			
17	qαKNL2-C	TCGACTTTGATGTGGAGGTAACAC	GAATCAGTAGACGCCGCATTGG
18	qCENH3	GCAGGTCCAACTACGACCC	GCTGGTGAAGTTGTAGGATTTGT
