# Supplementary material for: The C-terminal SUMOylation-dependent regulation of αKNL2 governs its centromere targeting and interaction with CENH3
Source: Plant Commun. 2025 Nov 19;7(2):101617. doi: 10.1016/j.xplc.2025.101617 (PMC12903445; doi:10.1016/j.xplc.2025.101617)
Supplement: Document S2. Article plus supplemental information [file mmc3.pdf]

Plant Communications
Research article

CellPress
Partner Journal

The C-terminal SUMOylation-dependent regulation of αKNL2 governs its centromere targeting and interaction with CENH3

Manikandan Kalidass[1,*], Jitka Vaculíková[2], Jothipriya Ramakrishnan Chandra[1], Barbora Královál[2], Venkata Ganesh Jarubula[1,3], Sevim D. Kara Öztürk[4], Dmitri Demidov[1], Veit Schubert[1], David Potesil[5], Jan J. Palecek[2,5] and Inna Lermontova[1,*]

[1]Leibniz Institute of Plant Genetics and Crop Plant Research (IPK) Gatersleben, Corrensstrasse 3, 06466 Seeland, Germany

[2]National Center for Biomolecular Research, Faculty of Science, Masaryk University, Kamenice 5, 62500 Brno, Czech Republic

[3]Department of Program Center Metacom, Leibniz Institute of Plant Biochemistry, Weinberg 3, 06120 Halle (Saale), Germany

[4]Department of Agricultural Genetic Engineering, Ayhan Şahenk Faculty of Agricultural Sciences and Technologies, Niğde Ömer Halisdemir University, Niğde 51240, Türkiye

[5]Central European Institute of Technology (CEITEC), Masaryk University, Kamenice 5, 62500 Brno, Czech Republic

***Correspondence: Manikandan Kalidass (kalidass@ipk-gatersleben.de), Inna Lermontova (lermonto@ipk.gatersleben.de)**

https://doi.org/10.1016/j.xplc.2025.101617

ABSTRACT

The centromere is a specialized domain that facilitates chromosome segregation during mitosis and serves as the site of kinetochore formation. KINETOCHORE NULL2 (αKNL2) is essential for the recognition and loading of the centromeric histone H3 variant CENH3 at centromeres. A yeast two-hybrid screen for αKNL2 interactors identified components of the SUMOylation pathway. However, the role of αKNL2 SUMOylation in *Arabidopsis* has not yet been determined. In this study, we demonstrated that the C-terminal region of αKNL2 (designated αKNL2-C) interacts with small ubiquitin-like modifier 3 (SUMO3) and ULP1d, as shown by bimolecular fluorescence complementation and co-immunoprecipitation assays. Bioinformatic and functional analyses of αKNL2-C identified three SUMOylation sites and two SUMO-interacting motifs, which were shown to be critical for growth, fertility, and chromosome alignment. Of the three SUMOylation sites, Lys474 and Lys511 are the most critical for the centromeric localization of αKNL2, underscoring the importance of αKNL2 SUMOylation for its function. Additionally, both *in vitro* and *in vivo* assays showed that αKNL2-C undergoes SUMOylation by SUMO1 or SUMO3. The *Arabidopsis* SUMO protease mutant *ulp1d-2* exhibits a mild accumulation of SUMOylated αKNL2. We further showed that SUMOylation of αKNL2 promotes its binding to CENH3 and controls protein stability. Our findings demonstrate that C-terminal SUMOylation of αKNL2 is crucial for its centromeric localization, interaction with CENH3, and kinetochore assembly, emphasizing the significance of post-translational modifications in chromosome segregation and cell division in plants.

Key words: cell division, centromere, kinetochore, SUMOylation, plant development, protein–protein interactions

Kalidass M., Vaculíková J., Chandra J.R., Královál B., Jarubula V.G., Kara Öztürk S.D., Demidov D., Schubert V., Potesil D., Palecek J.J., and Lermontova I. (2025). The C-terminal SUMOylation-dependent regulation of αKNL2 governs its centromere targeting and interaction with CENH3. Plant Comm. **7**, 101617.

INTRODUCTION

Centromeres are essential chromosomal regions that ensure accurate chromosome segregation during cell division. They serve as assembly sites for the kinetochore, a multiprotein complex that interacts with spindle microtubules to facilitate chromosome movement. Centromeric histone H3 (CENH3; known as CENP-A in humans) acts as a marker for active centromeres and is crucial for kinetochore establishment (Talbert et al., 2002; Naish and Henderson, 2024). KINETOCHORE NULL2 (αKNL2) is a key kinetochore protein that plays a pivotal role in CENH3 loading at centromeres and kinetochore assembly. As a upstream component of the constitutive centromere-associated network (CCAN), αKNL2 forms a structural bridge connecting CENH3 nucleosomes with outer kinetochore components. *Arabidopsis* αKNL2 contains a conserved N-terminal Swi3-Ada2-NCoR-TFIIIB-associated

© 2025 The Authors. Published by Elsevier Inc. on behalf of CAS Center for Excellence in Molecular Plant Sciences, Chinese Academy of Sciences, Chinese Society for Plant Biology. This is an open access article under the CC BY license (http://creativecommons.org/licenses/by/4.0/).

(SANTA)domain, similar to KNL2 in other organisms (Zhang et al., 2006). Most vertebrate and plant KNL2 homologs also possess a C-terminal CENPC-k motif that facilitates binding to CENH3 nucleosomes (French et al., 2017; Hori et al., 2017; Sandmann et al., 2017). Deletion of the CENPC-k motif or mutation of its single conserved amino acid abolishes the centromeric localization of αKNL2 (Sandmann et al., 2017). Reduced levels of CENH3 protein in a *knl2* knockout mutant have been linked to micronuclei in pollen tetrads, anaphase bridges during mitosis, and a 30% seed abortion rate (Lermontova et al., 2013). In *Arabidopsis thaliana* (*A. thaliana)*, full-length αKNL2 cannot be stably overexpressed due to its targeted degradation via the ubiquitin–proteasome system, a process orchestrated by APC/C^{CDC20}-mediated ubiquitination of αKNL2 that is essential for maintaining kinetochore function and centromere integrity (Lermontova et al., 2013; Kalidass et al., 2025). This highlights the importance of post-translational modification (PTM) of αKNL2 for mitotic fidelity and plant development.

PTMs play key roles in virtually all cellular processes and include covalent modifications such as phosphorylation, ubiquitination, and SUMOylation. Ubiquitin polymers are well known for their classical role in targeting proteins to the proteasome for degradation. Although proteasome-mediated degradation is a common fate of ubiquitinated proteins, the small ubiquitin-like modifier (SUMO) is often associated with the modulation of protein–protein interactions via non-covalent binding to SUMO-interacting motifs (SIMs), a process that can also influence protein localization (Mahajan et al., 1998; Matunis et al., 1998; Pichler et al., 2005). SUMO also shares structural similarity with ubiquitin, and during SUMOylation, it is covalently attached to lysine residues in substrate proteins through a cascade of E1 activating, E2 conjugating, and E3 ligase enzymes. In *Arabidopsis*, there are four SUMO isoforms: SUMO1, SUMO2, SUMO3, and SUMO5. Among these, SUMO1 and SUMO2 are nearly identical and function redundantly, whereas SUMO3 and SUMO5 exhibit more specialized roles. Under normal physiological conditions, SUMOs primarily function as monomers and mediate signaling processes. Their activity is tightly regulated by SUMO-specific proteases, which remove SUMO modifications from target proteins in a process known as deSUMOylation. SUMO deconjugation is performed by the conserved ubiquitin-like protease/sentrin-specific protease (ULP/SENP) family of SUMO-specific proteases. The *Arabidopsis* ULP family has at least seven members (ESD4, ULP1a/ELS1, ULP1b, ULP1c/OTS2, ULP1d/OTS1, ULP2a, and ULP2b), which may contribute to both specificity and redundancy within the SUMO pathway (Chosed et al., 2006; Colby et al., 2006). ESD4 and ULP1a were previously associated with the control of flowering time and plant development (Murtas et al., 2003; Hermkes et al., 2011). ULP1c and ULP1d have been implicated in salt stress responses, modulation of salicylic acid (SA) signaling, and deSUMOylation of phytochrome B (Conti et al., 2008, 2014; Sadanandom et al., 2015; Bailey et al., 2016).

SUMOylation is a crucial modification of chromatin proteins, including histone H3. Centromere- and kinetochore-associated factors are reportedly enriched among SUMOylated proteins in several species (Azuma et al., 2003; Montpetit et al., 2006; Zhang et al., 2008; Mukhopadhyay et al., 2010; Li et al., 2016), and both SUMO E3 ligase and SUMO protease enzymes have been found to colocalize with kinetochores (Ban et al., 2011; Cubeñas-Potts et al., 2013; Suhandynata et al., 2019). In contrast, recent evidence suggests that SUMOylation of the kinetochore protein Nuf2 is required to promote the recruitment of the SIM-containing centromere-associated protein CENP-E, which is essential for the proper alignment of chromosomes in metaphase (Subramonian et al., 2021). In *A. thaliana*, the conserved ATPase Associated with diverse cellular Activities (AAA+) and molecular chaperone CDC48/p97 interacts with SUMOylated CENH3 to remove it from centromeres, leading to disruption of centromeric heterochromatin and activation of rRNA genes (Mérai et al., 2014). Nevertheless, previous reports show that the SUMO protease SENP6 mediates MIS18BP1/HsKNL2 deSUMOylation to regulate CENP-A loading in humans. SENP6 depletion leads to RNF4-mediated degradation of HsKNL2 and, consequently, failure of CENP-A to accumulate at centromeres (Fu et al., 2019; Liebelt et al., 2019). HsKNL2 is co-modified by SUMO and ubiquitin and is subsequently targeted by the SUMO-targeted ubiquitin ligase RNF4. These findings provide evidence that SUMO–ubiquitin crosstalk regulates HsKNL2 during mitosis (Cuijpers et al., 2017) and indicate that the timing and specificity of kinetochore localization are regulated by SUMOylation. Nonetheless, the role of SUMOylation at centromeres in plants appears to be complex and remains to be fully elucidated.

In this study, we found that SUMOylation of *Arabidopsis* αKNL2 is critical for its localization and interaction with CENH3 at centromeres. We demonstrated that SUMO3 and ULP1d interact with αKNL2 using yeast two-hybrid (Y2H) library screening, bimolecular fluorescence complementation (BiFC), and co-immunoprecipitation (coIP). In addition, we identified SUMOylation and SIM sites in αKNL2-C that are essential for its centromeric localization. Among these, SUMOylation at Lys474 and Lys511 is crucial for αKNL2 SUMOylation. Accumulation of the SUMOylation-deficient αKNL2 mutant led to defects in growth, fertility, and mitosis. *In vitro* and *in vivo* SUMOylation assays showed that the C-terminal region of αKNL2 (designated αKNL2-C) is modified by SUMO1 or SUMO3. Additionally, we found that the *ulp1d-2* mutant exhibited accumulation of SUMOylated αKNL2. Our study also revealed that αKNL2-C interacts with CENH3, whereas this interaction was disrupted in the SUMOylation-deficient αKNL2 mutant.

RESULTS

SUMO3 and ULP1d interact with the C terminus of αKNL2

Affinity purification–mass spectrometry (AP–MS) and Y2H library screening were used to identify proteins that interact with αKNL2. For this purpose, the full-length *Arabidopsis* αKNL2 protein (αKNL2, amino acids [aa] 1–598), along with its N-terminal (αKNL2-N, aa 1–363) and C-terminal (αKNL2-C, aa 364–598) fragments, was used (Figure 1A). Pathway enrichment analysis revealed that αKNL2 is involved in processes such as PTMs, metabolism, and transcription (Kalidass et al., 2025). To further investigate the functional roles of these interacting proteins, Gene Ontology enrichment analysis was performed, focusing on biological processes and molecular functions related to PTMs. These included biological process and molecular

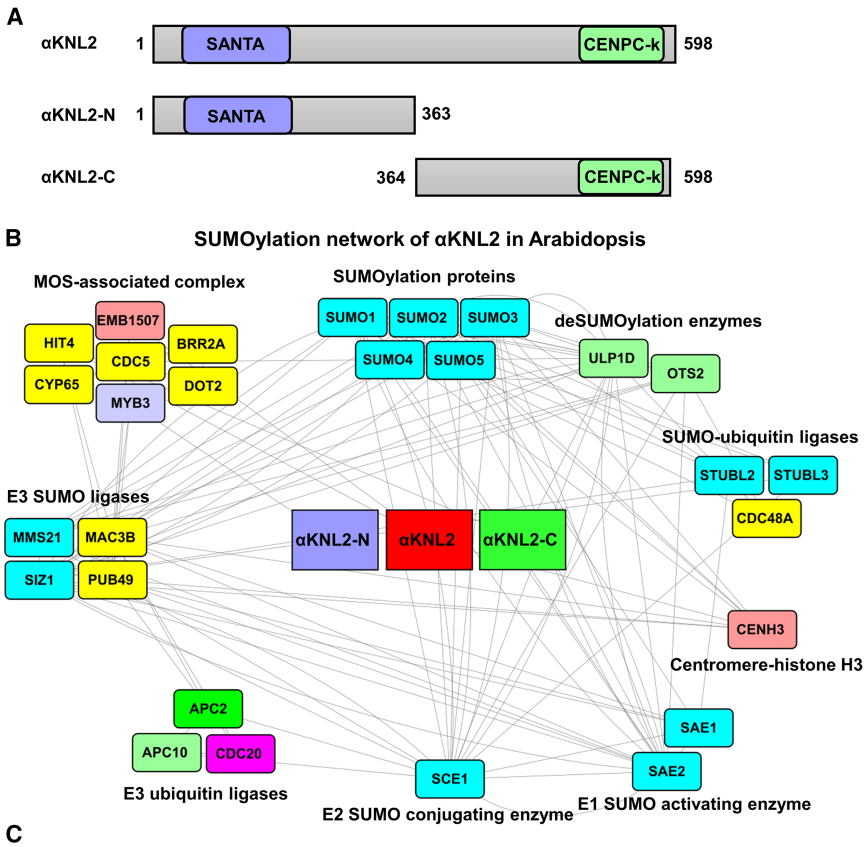

C

TAIR ID	Description	Blast hit	No. of clones
AT1G60220	*Arabidopsis thaliana* UB-like protease 1D (ULP1d)	Protein is similar to the clone	3
AT1G10570	*Arabidopsis thaliana* Cysteine proteinases superfamily protein (ULP1c)	Protein is partially similar to the clone	

Figure 1. The αKNL2 interactome reveals associations with the SUMOylation machinery in *Arabidopsis*.
(A) Schematic illustration of the domain organization of the αKNL2 protein (aa 1–598), highlighting its N-terminal (αKNL2-N, aa 1–363) and C-terminal (αKNL2-C, aa 364–598) regions. The SANTA domain (purple box) is located in the N-terminal region, and the C-terminal region contains the conserved CENPC-k motif (green).
(B) Protein–protein interaction network for αKNL2 generated from Y2H library screening and AP–MS results. Rectangular boxes represent αKNL2 interactors grouped by functional annotations. Interactors identified through AP–MS are shown in yellow boxes, whereas those identified via Y2H are displayed in colored boxes. Proteins in blue boxes, identified through STRING, were used to connect pathways but were not identified as αKNL2 interactors. The network was constructed using STRING and Cytoscape software.
(C) Sequencing analysis of αKNL2-C clones from the Y2H screening identified ULP1d as an interactor.

function categories such as ubiquitination, SUMOylation, nucleocytoplasmic transport, and RNA export from the nucleus (Supplemental Figure 1). Furthermore, protein interaction network analysis indicated that αKNL2 is regulated by SUMOylation pathways and revealed a specific subnetwork of SUMOylation-related αKNL2 interactors (Figure 1B).

To investigate the role of SUMOylation in αKNL2 regulationULP1d, a SUMO protease, along with the SUMO isoforms SUMO1, SUMO2, SUMO3, and SUMO5, were tested for interaction with αKNL2 using BiFC. In the BiFC assay, full-length αKNL2, αKNL2-N, and αKNL2-C were fused to the N-terminal half of Venus (VENn), whereas ULP1d and the SUMO isoforms were fused to the C-terminal half of Venus (VENc), and vice versa. Interaction analysis revealed that αKNL2-C interacts with ULP1d and SUMO3 in the nucleolus (Figure 2A and Supplemental Figure 2). BiFC analysis further showed that αKNL2-C specifically interacts with the C-terminal region of ULP1d in the nucleolus (Figure 2A). BiFC quantification consistently showed strong interactions between αKNL2-C and both SUMO3 and ULP1d-C, as indicated by a high number of nuclei displaying BiFC fluorescence signals and by increased fluorescence intensity (Figures 2B and 2C). Moreover, full-length αKNL2 and αKNL2-N showed no interactions with ULP1d or SUMO3 when fused to either half of Venus. Furthermore, none of the αKNL2 fragments interacted with SUMO1, SUMO2, or SUMO5 (Supplemental Figure 2), which suggests that SUMO3 specifically binds to αKNL2.

To further validate the interactions between αKNL2 and components of the SUMOylation pathway, a coIP assay was performed. Specifically, αKNL2-C^{HA} was co-expressed with SUMO3^{cMYC} and ULP1d-C^{HA} was co-expressed with αKNL2-C^{cMYC} in *Nicotiana benthamiana* (*N. benthamiana)* leaves. As a negative control, αKNL2-C^{cMYC} was co-expressed with an empty hemagglutinin (HA) vector. In all cases, total protein extracts were subjected to IP using HA magnetic beads. Subsequent western blot analysis with an anti-cMYC antibody detected SUMO3^{cMYC} and αKNL2-C^{cMYC} when co-expressed with αKNL2-C^{HA} or ULP1d-C^{HA} but not with the empty HA control (Figure 2D). These findings corroborate the BiFC and Y2H results and confirm the interactions of αKNL2-C with SUMO3 and ULP1d.

SUMOylation sites in the C terminus of αKNL2 regulate its centromere targeting

The increasing identification of SUMOylation sites in eukaryotic cells has enabled the development of computational tools, such as group-based prediction system for SUMOylation (GPS-SUMO) (http://sumosp.biocuckoo.org/), to predict potential SUMOylation targets. Using this tool, three lysine residues—K378, K474, and K511—were identified as potential SUMOylation sites in *Arabidopsis* αKNL2-C. In addition, two SIMs were identified in the C-terminal region, at residues

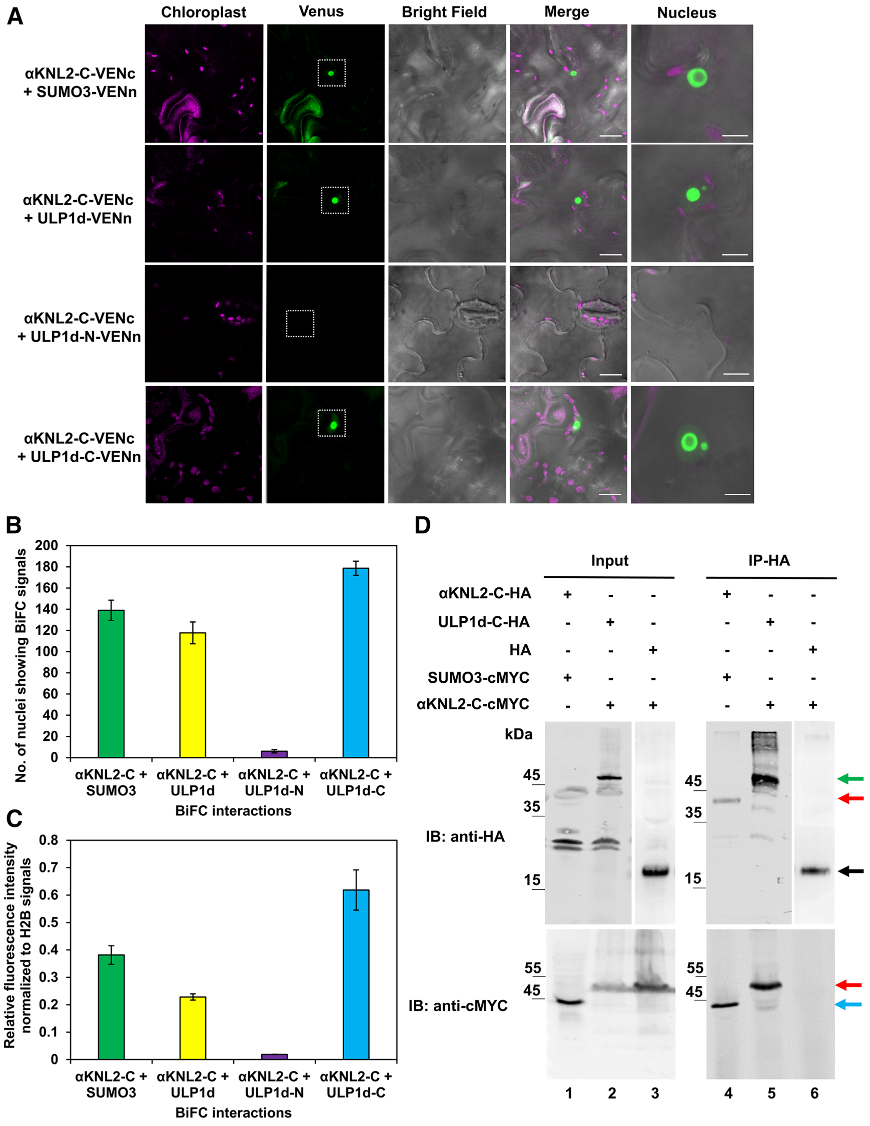

Figure 2. Interaction of the αKNL2 C terminus with SUMO pathway components.

(A) BiFC analysis showing interactions between αKNL2-C fused to VENc and SUMO3, ULP1d, ULP1d-N, or ULP1d-C fused to VENn. White dotted boxes indicate BiFC signals (Venus fluorescence). Scale bars: 50 μm. Right: a magnified view of the BiFC signals in the nucleolus. Enlarged images may not always correspond to the same nuclei shown in the overview image. Scale bars: 5 μm.

(B and C) Bar graphs showing the number of nuclei with BiFC signals **(B)** and the corresponding mean fluorescence intensity **(C)** for each interaction pair. The number of nuclei exhibiting BiFC signals was measured in an 80 mm^2 area. Fluorescence intensity was normalized using H2B signals (n = 30 nuclei per sample). Data are presented as mean ± SEM.

(D) CoIP analysis showing interactions between αKNL2-C and SUMO3 or ULP1d-C. *N. benthamiana* leaves were infiltrated with constructs encoding αKNL2-C-HA and SUMO3-cMYC (lanes 1 and 4), ULP1d-C-HA and αKNL2-C-cMYC (lanes 2 and 5), or HA and αKNL2-C-cMYC (lanes 3 and 6). Total protein extracts were immunoprecipitated using anti-HA magnetic beads, and samples were analyzed by immunoblotting with anti-HA and anti-cMYC antibodies before (input) and after immunoprecipitation (IP). Interactions between αKNL2-C and SUMO3 or ULP1d-C were detected by anti-cMYC immunoblotting, whereas no interaction was detected in the empty-HA control. Red, green, blue, and black arrows indicate the molecular weights (MWs) of αKNL2-C, ULP1d-C, SUMO3, and empty HA, respectively. All HA-tagged constructs included a VENc fusion, and all cMYC-tagged constructs included a VENn fusion, which increase the MWs of the constructs accordingly. IB, immunoblot.

547–551 and 568–572. Sequence alignment of αKNL2 homologs from Brassicales genomes confirmed the conservation of these SUMOylation and SIM sites (Figure 3A and Supplemental Figures 3A–3C).

Consequently, a SUMOylation-deficient αKNL2-C mutant was generated in which these three lysine residues were substituted with arginine (K→R) and the SIMs were deleted (αKNL2-$C^{Mut\text{-}SUMO}$). This mutant was fused to enhanced yellow fluorescent protein (EYFP) and expressed in *N. benthamiana* plants under the 35S promoter. Co-localization assays in *N. benthamiana* showed that wild-type αKNL2-C co-localized with CENH3 at centromeres, whereas the SUMOylation-deficient mutant failed to localize to centromeres and instead accumulated in the nucleoplasm and cytoplasm (Figures 3B–3D). To further investigate the *in vivo* localization of αKNL2-$C^{Mut\text{-}SUMO}$ in *A. thaliana*, stable transgenic lines expressing either αKNL2-C-EYFP or αKNL2-$C^{Mut\text{-}SUMO}$-EYFP fusion constructs were generated. Root tip analysis of at least three independent T2 lines expressing αKNL2-$C^{Mut\text{-}SUMO}$-EYFP showed a clear loss of αKNL2-specific centromeric signals relative to the unmutated variant. Additionally, the fluorescence was largely distributed throughout the cytoplasm and nucleoplasm and, in some cases, the nucleolus (Figures 3E and 3F). Western blot analysis using an anti-GFP antibody confirmed comparable αKNL2 protein levels across all three independent lines for both the wild-type and SUMOylation-deficient constructs, indicating that the observed localization differences are not due to differences in protein expression (Supplemental Figure 4). In addition, BiFC analysis of the SUMOylation-deficient αKNL2-C mutant showed no fluorescence when co-expressed with ULP1d, ULP1d-C, or SUMO3, which indicates that the SUMOylation sites and SIMs are crucial for αKNL2-C binding to SUMO3 and ULP1d (Supplemental Figures 5A–5D). These findings demonstrate that SUMOylation and/or SUMO interaction are crucial for the centromeric localization of αKNL2.

To further dissect the roles of SUMOylation and SUMO interaction, site-directed mutagenesis was performed on each of the three predicted SUMOylation sites and two SIMs in αKNL2-C. The resulting mutated constructs were fused to EYFP and transiently expressed in *N. benthamiana* leaves. Surprisingly, all

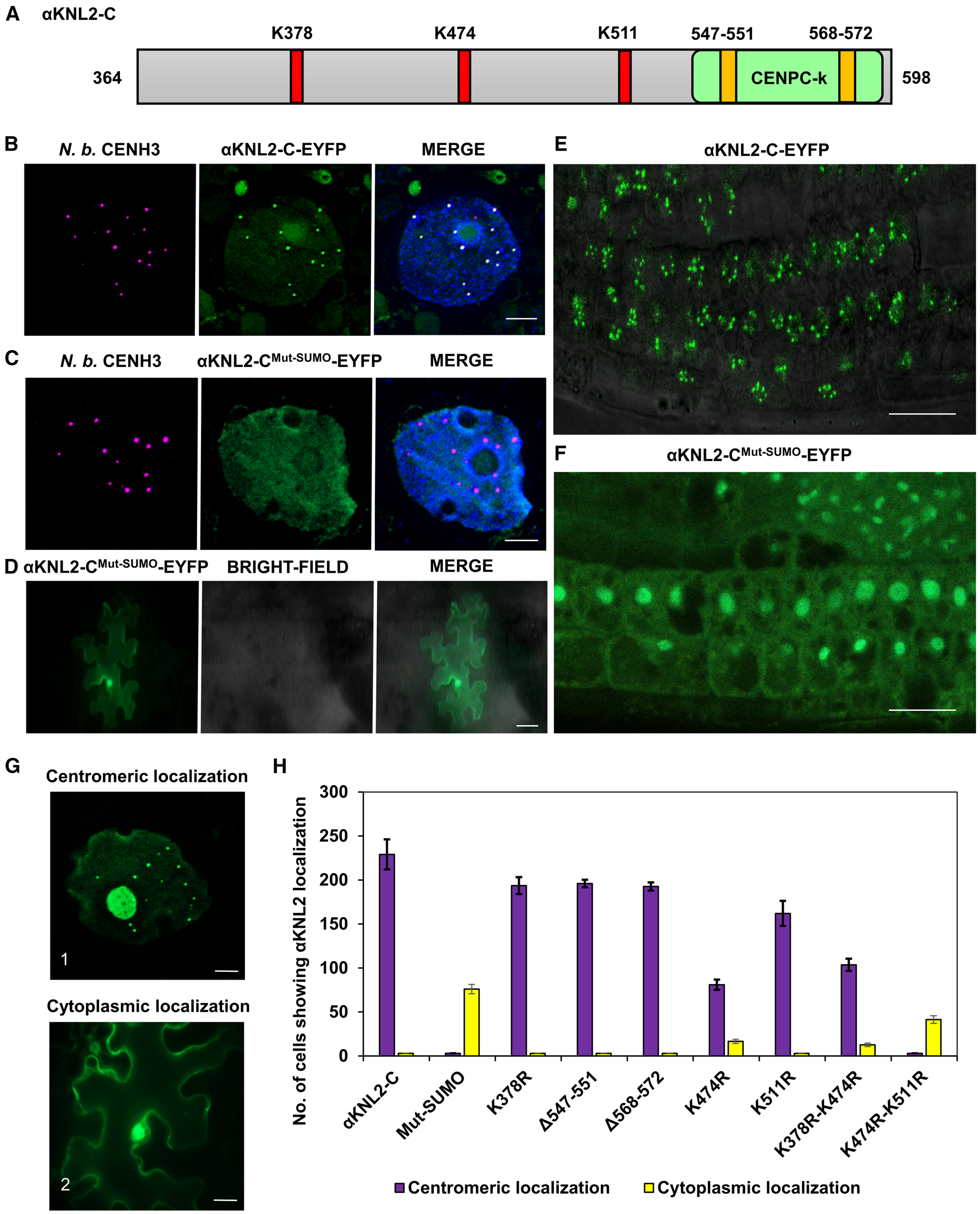

Figure 3. The SUMOylation-deficient mutant of αKNL2-C disrupts its centromere targeting.

(A) The C-terminal part of αKNL2 (αKNL2-C, aa 364–598) contains three conserved lysine **(K)** residues (K378, K474, and K511; red boxes) and two SUMO interaction sites (aa 547–551 and 568–572; orange boxes).

(B) Co-localization of αKNL2-C-EYFP (green) with CENH3 (magenta) in *N. benthamiana*, indicating centromere-specific signals. Scale bars: 5 μm.

(C and D) Localization patterns of the SUMOylation-deficient αKNL2 mutant (αKNL2-C$^{Mut\text{-}SUMO}$-EYFP) in *N. benthamiana* leaves. The construct showed nucleoplasmic signals **(C)** that did not co-localize with *N. benthamiana* CENH3 at centromeres and cytoplasmic localization **(D)**. Scale bars: 5 μm **(C)** and 50 μm **(D)**.

(E and F) Localization of αKNL2-C-EYFP **(E)** and αKNL2-C$^{Mut\text{-}SUMO}$-EYFP **(F)** in *Arabidopsis* root tips, resembling the patterns observed in *N. benthamiana*. Scale bars: 10 μm.

(legend continued on next page)

single-lysine mutants displayed centromere-specific localization. Specifically, αKNL2-C^{K378R}-EYFP, αKNL2-C^{K511R}-EYFP, αKNL2-C$^{\Delta 547\text{-}551}$-EYFP, and αKNL2-C$^{\Delta 568\text{-}572}$-EYFP retained centromeric localization, whereas αKNL2-C^{K474R}-EYFP showed both centromeric and cytoplasmic localization (Figure 3G). Therefore, αKNL2 double mutants were generated, targeting lysine residues 378 and 474 (K378R/K474R) or 474 and 511 (K474R/K511R). The localization pattern of αKNL2-C$^{K378R/K474R}$-EYFP resembled that of the K474R single mutant. In contrast, αKNL2-C$^{K474R/K511R}$-EYFP was predominantly mislocalized to the cytoplasm, with fewer cells showing centromere-associated signals (Figure 3G). Quantification of the fluorescence patterns revealed that αKNL2-C^{K378R}-EYFP, αKNL2-C^{K511R}-EYFP, αKNL2-C$^{\Delta 547\text{-}551}$-EYFP, and αKNL2-C$^{\Delta 568\text{-}572}$-EYFP displayed centromeric localization (198–211 nuclei per 80 mm^2) comparable to the wild-type αKNL2-C-EYFP (220–232 nuclei per 80 mm^2). In contrast, αKNL2-C^{K474R}-EYFP and αKNL2-C$^{K378R/K474R}$-EYFP exhibited partial loss of centromeric localization (81–103 nuclei per 80 mm^2) along with cytoplasmic signals (12–16 cells per 80 mm^2). Notably, αKNL2-C$^{K474R/K511R}$-EYFP showed nucleoplasmic and cytoplasmic signals (42 cells per 80 mm^2), similar to the pattern of αKNL2-C$^{Mut\text{-}SUMO}$-EYFP (76 cytoplasmic cells per 80 mm^2) (Figure 3H). In addition, BiFC analysis showed that αKNL2-C$^{K474R/K511R}$ produced no detectable fluorescence when co-expressed with ULP1d, ULP1d-C, or SUMO3, unlike other single- or double-lysine mutants (Supplemental Figure 5E). These findings suggest that SUMOylation at Lys474 and Lys511 is critical for both the interaction with SUMO pathway components and the centromeric targeting of αKNL2-C.

The SUMOylation-deficient αKNL2 mutant exhibits impaired plant development and mitosis

Given that the SUMOylation-deficient αKNL2 mutant showed disrupted centromere targeting in *N. benthamiana* and *Arabidopsis*, its effects on plant growth and development were investigated. Transgenic *Arabidopsis* plants expressing αKNL2-C-EYFP did not exhibit any phenotypic differences compared with wild-type (Col-0) plants (Lermontova et al., 2013). Therefore, the αKNL2-C-EYFP line was used as a control to assess the effect of the SUMOylation-deficient αKNL2 mutant. Following fluorescence screening of 12 independent transgenic lines, 3 lines exhibiting reproducible and uniform expression were selected for further analysis. Analysis of these 3 independent lines expressing the αKNL2-C$^{Mut\text{-}SUMO}$-EYFP fusion construct revealed an average reduction in root length of 28.14% compared with αKNL2-C-EYFP plants (Figures 4A and 4B). In addition, plants expressing αKNL2-C$^{Mut\text{-}SUMO}$ exhibited significant differences in vegetative growth and development (Figure 4C). Previous studies have demonstrated that the *αknl2* knockout mutant and lines expressing degradation-resistant αKNL2 variants with mutations in ubiquitination sites display mitotic defects and reduced fertility (Lermontova et al., 2013; Kalidass et al., 2025). Based on these findings, we hypothesized that overexpression of αKNL2-C$^{Mut\text{-}SUMO}$-EYFP, which fails to localize to centromeres in *Arabidopsis*, may lead to mitotic abnormalities.

Consistent with this hypothesis, analysis of root tip meristems from 3 independent transgenic lines expressing αKNL2-C$^{Mut\text{-}SUMO}$-EYFP revealed mitotic abnormalities. On average, 26% of the analyzed cells (8 of 30 from each line) displayed misaligned metaphase chromosomes (Figures 4D and 4E and Supplemental Figure 6A). Fertility assessments also revealed impaired reproductive development in αKNL2-C$^{Mut\text{-}SUMO}$-EYFP plants, as evidenced by reduced silique size relative to αKNL2-C-EYFP plants across three independent lines (Figures 4F and 4G). However, pollen viability was unaffected, as confirmed by Alexander staining (Supplemental Figure 6B). Furthermore, seed analysis from 10 siliques of a representative αKNL2-C$^{Mut\text{-}SUMO}$-EYFP line showed that, on average, 20% of seeds were aborted and 18% were shriveled (Supplemental Figures 6B and 6C). These findings suggest that SUMOylation of αKNL2 is crucial for mitotic progression and fertility in *Arabidopsis*.

In vivo and *in vitro* SUMOylation reveals isoform-specific modification of αKNL2

To investigate whether αKNL2 undergoes SUMOylation *in planta*, total proteins were extracted from leaves of *N. benthamiana* infiltrated with constructs expressing αKNL2-C-EYFP, αKNL2-C$^{Mut\text{-}SUMO}$-EYFP, or EYFP alone. The proteins were immunoprecipitated using anti-GFP affinity beads and analyzed by immunoblotting with either an anti-GFP or anti-SUMO antibody. Immunoblotting with anti-GFP detected bands at the expected molecular weight (MW) of αKNL2-C-EYFP (~55 kDa) and at higher MWs, suggesting PTMs of αKNL2-C. In contrast, the αKNL2-C$^{Mut\text{-}SUMO}$-EYFP sample showed markedly reduced or absent higher-MW bands, which indicates that the lysines mutated in this construct may serve as potential SUMOylation sites (Figure 5A). To examine the SUMOylation of αKNL2, specific antibodies against SUMO1 and SUMO3 were used. We found that SUMO3 was strongly conjugated to αKNL2-C, producing distinct higher-MW bands (>55 kDa) that were strongly reduced in the SUMOylation-deficient mutant (Figure 5A). To further test whether K474 and K511 serve as SUMO acceptor sites, as suggested by the BiFC results, we performed a SUMOylation assay with anti-SUMO3 antibodies. This analysis showed that the single K474R and K511R mutants behaved similarly to wild-type αKNL2-C, whereas the double K474R/K511R mutation markedly reduced SUMOylation (Supplemental Figure 7A). In addition, anti-SUMO1 immunoblotting revealed that αKNL2-C is also modified by SUMO1, although this modification was only mildly reduced in the SUMOylation-deficient mutant (Supplemental Figure 7B). No SUMOylated bands were detected in the EYFP pull-down controls (Figure 5A and Supplemental Figure 7B). Together, these results demonstrate that K474 and K511 function redundantly as the major αKNL2 SUMOylation sites, with SUMO3 conjugation being strongly dependent on both residues. This confirms that the modifications observed in

(G) Centromere-specific signals (1) and cytoplasmic localization patterns (2) were observed for individual SUMOylation and SIM site mutations in αKNL2-C. Scale bars: 5 μm.

(H) Quantitative analysis of cells exhibiting distinct fluorescence patterns from **(G)**, comparing SUMOylation-deficient mutants with αKNL2-C as a control. The number of cells exhibiting αKNL2 localization was quantified within an 80 mm^2 area. For some constructs, zero values were plotted as 3 to aid visualization. Data are presented as mean ± SEM.

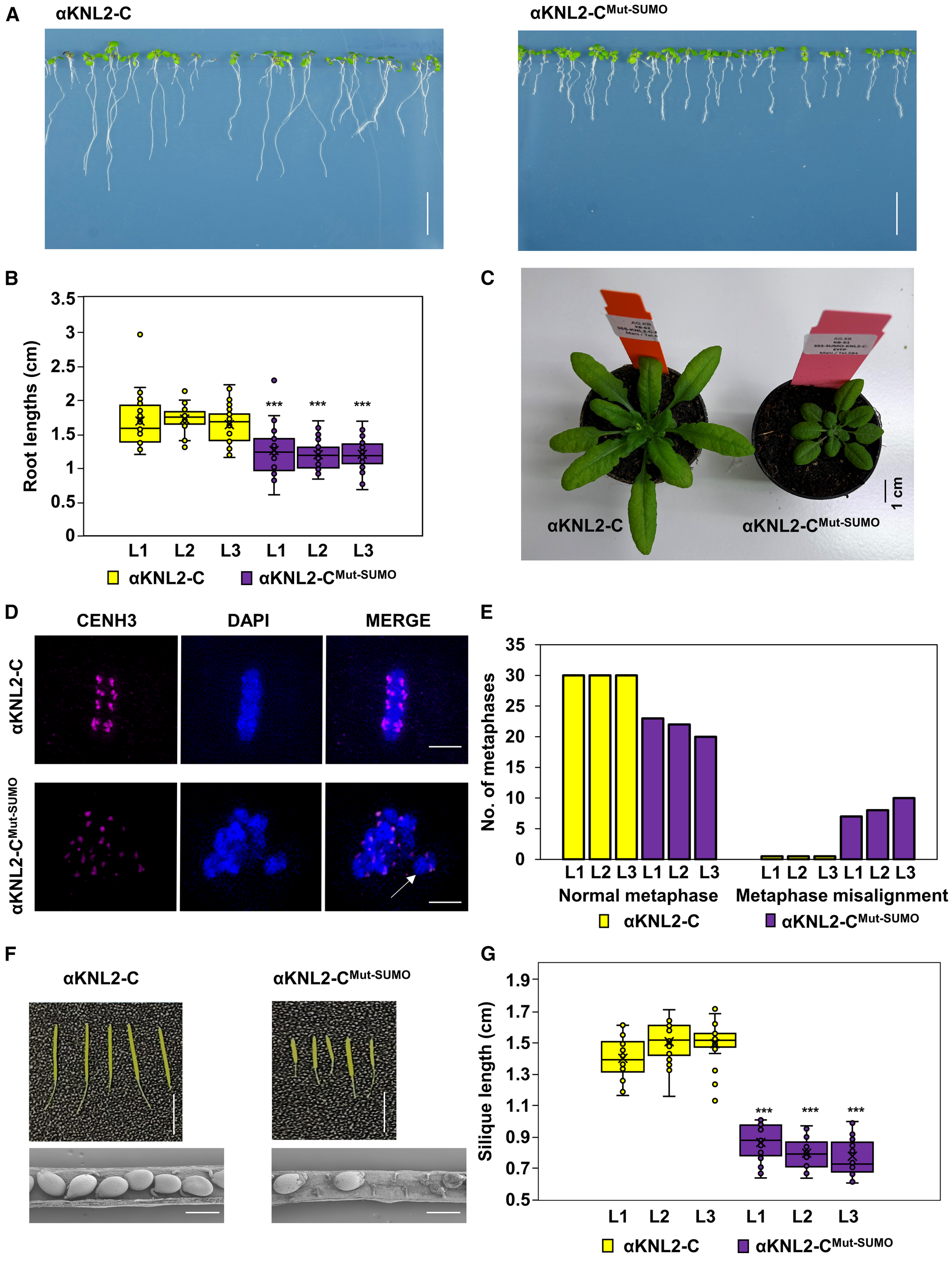

Figure 4. Phenotypic analysis of the SUMOylation-deficient αKNL2 mutant.

(A) Root growth phenotype of 7-day-old *Arabidopsis* seedlings expressing αKNL2-C$^{Mut\text{-}SUMO}$-EYFP compared with αKNL2-C-EYFP. Scale bars: 1 cm.

(B) Boxplot showing primary root lengths in αKNL2-C-EYFP and αKNL2-C$^{Mut\text{-}SUMO}$-EYFP seedlings. Seven-day-old seedlings from three independent transgenic lines per construct were analyzed (n = 25 seedlings per line). Boxplots show the median (horizontal line), interquartile range (box), data range (whiskers), and individual data points. Means are marked by ×. αKNL2-C$^{Mut\text{-}SUMO}$-EYFP lines exhibited shorter primary roots than αKNL2-C-EYFP lines. Statistical significance was determined using Welch's t-test; ***$p < 0.005$.

(C) Phenotypic comparison of 5-week-old plants expressing αKNL2-C$^{Mut\text{-}SUMO}$-EYFP and αKNL2-C-EYFP grown in soil.

(legend continued on next page)

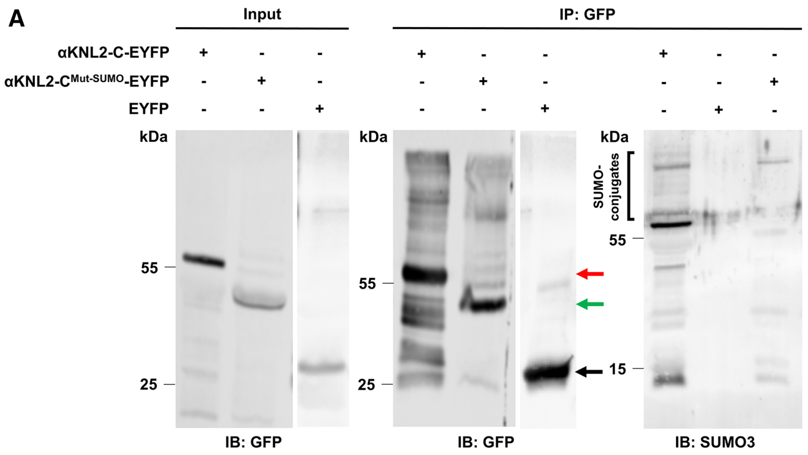

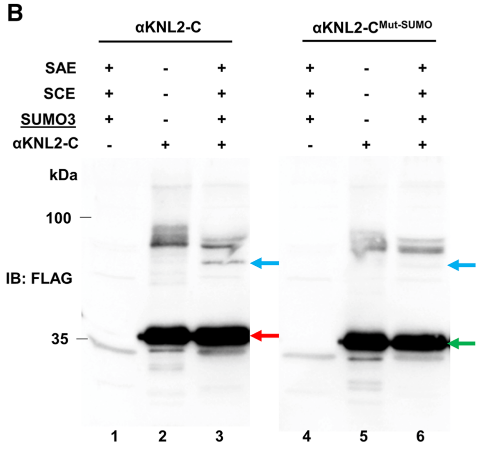

C

MS analysis of SUMO3/H88K	
Lysine modified	Peptide detected
K424	DVCVAINNEVISPVKGFGK
K511	KIDFDVEVTPEK
K598	IKAENLYFQGDYK
K598	IKAENLYFQGDYKDDDDK

Figure 5. *In vivo* and *in vitro* SUMOylation analysis of αKNL2.

(A) *In vivo* SUMOylation analysis of αKNL2 in *N. benthamiana* leaves expressing αKNL2-C-EYFP or αKNL2-C$^{Mut\text{-}SUMO}$-EYFP. Leaves expressing EYFP alone served as controls. Total protein extracts were immunoprecipitated using anti-GFP beads. Input samples were probed with an anti-GFP antibody, whereas immunoprecipitated samples were probed with anti-GFP or anti-SUMO3. Red, green, and black arrows denote the MWs of αKNL2-C, αKNL2-C$^{Mut\text{-}SUMO}$, and EYFP, respectively. SUMO conjugates are highlighted by black brackets.

(B) *In vitro* SUMOylation assays with enzymes only (lanes 1 and 4), substrate only (lanes 2 and 5), and a mixture of enzymes and substrates (lanes 3 and 6). After incubation, samples were analyzed using SDS-PAGE followed by anti-FLAG immunoblotting to detect αKNL2. The full, uncropped blot, including the Ponceau S loading control, is provided in Supplemental Figure 7. The red arrow indicates unmodified αKNL2-C, the green arrow indicates the unmodified αKNL2-C$^{Mut\text{-}SUMO}$ mutant, and the blue arrows indicate SUMOylated forms. Mutations of the conserved Lys residues reduced SUMOylation efficiency compared with the wild-type variant.

(C) Peptides detected by MS analysis, with lysines modified by SUMO3 highlighted in red.

wild-type αKNL2-C were indeed due to SUMOylation of the conserved lysine residues.

To complement the *in vivo* observations, an *in vitro* SUMOylation assay (Tomanov et al., 2022) was performed to directly evaluate the SUMOylation efficiency of αKNL2-C and its SUMOylation-deficient variant. Purified αKNL2-C proteins (αKNL2-C and αKNL2-C$^{Mut\text{-}SUMO}$; Figure 5B, lanes 2 and 5, and Supplemental Figure 7C) were incubated at 30°C for 2 h with a minimal enzymatic system comprising the E1 SUMO-activating enzyme (SAE), the E2 SUMO-conjugating enzyme (SCE), and the SUMO3 isoform (Figure 5B, lanes 1 and 4). Samples were analyzed using SDS-PAGE followed by western blotting with an anti-FLAG antibody to detect both unmodified and SUMOylated αKNL2. In reactions containing αKNL2-C, a distinct higher-MW band corresponding to the SUMOylated form was observed (Figure 5B, lane 3). In contrast, reactions with αKNL2-C$^{Mut\text{-}SUMO}$ exhibited significantly reduced SUMOylation levels, as indicated by the much weaker signal of the SUMOylated band (Figure 5B, lane 6). The *in vitro* assay results align with the *in vivo* findings, providing further evidence that SUMOylation by SUMO3 is a key PTM that regulates αKNL2 function. Importantly, the inability of αKNL2-C$^{Mut\text{-}SUMO}$ to be SUMOylated by SUMO3 highlights the critical role of the conserved SUMOylation sites K474 and K511. To further investigate the roles of different SUMO isoforms, similar *in vitro* reactions were conducted with SUMO1. Interestingly, in this setup, no significant reduction in SUMOylation was observed for αKNL2-C$^{Mut\text{-}SUMO}$ (Supplemental Figure 7D, left, lanes 3 and 7), indicating that SUMO1 can SUMOylate αKNL2$^{Mut\text{-}SUMO}$ even in the absence of the conserved SUMOylation sites. This suggests that SUMO1 may modify alternative, less conserved sites on αKNL2. In

(D) Mitotic metaphase images of αKNL2-C-EYFP and αKNL2-C$^{Mut\text{-}SUMO}$-EYFP plants visualized by spatial super-resolution structured illumination microscopy, with misaligned chromosomes highlighted (white arrows). Scale bars: 5 μm.

(E) Quantification of abnormal metaphases in a SUMOylation-deficient αKNL2 mutant. Analysis of 30 metaphase cells per line showed that 26% of metaphases in αKNL2-C$^{Mut\text{-}SUMO}$-EYFP plants displayed misalignment. Data represent three independent transgenic lines. Significant differences between groups were assessed using Welch's *t*-test; ***$p < 0.05$.

(F) Comparison of silique size between αKNL2-C-EYFP and αKNL2-C$^{Mut\text{-}SUMO}$-EYFP plants (top). Scale bars: 1 cm. Lower panel: scanning electron microscopy images of siliques. Scale bars: 20 μm.

(G) Boxplot showing silique length in αKNL2-C-EYFP and αKNL2-C$^{Mut\text{-}SUMO}$-EYFP plants. Siliques from 3 independent transgenic lines per construct were analyzed ($n = 25$ siliques per line). The boxplots show the median (horizontal line), interquartile range (box), data range (whiskers), and individual data points. Means are indicated by ×. Statistical significance was assessed using Welch's *t*-test; ***$p < 0.005$.

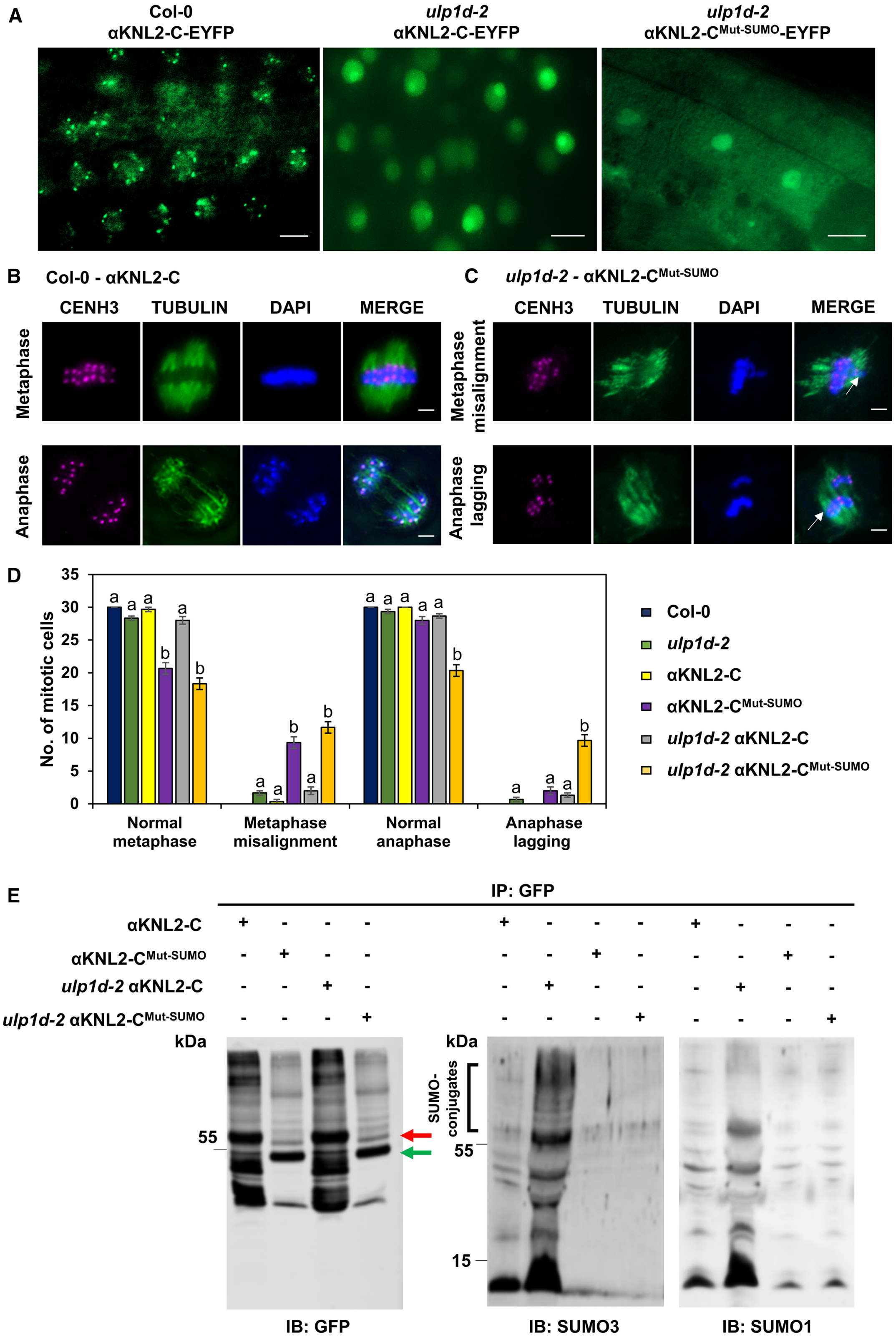

Figure 6. The accumulation of αKNL2 at centromeres is impaired in the absence of ULP1d.
(A) *Arabidopsis* root tips showing localization of αKNL2-C-EYFP in wild-type and *ulp1d-2* plants, and αKNL2-C$^{Mut\text{-}SUMO}$-EYFP in *ulp1d-2* plants. Scale bars: 10 μm.

(legend continued on next page)

addition, SUMO1 conjugation was enhanced by the E3 ligase NSE2, whereas SUMO3 conjugation was not (Supplemental Figures 7C and 7D; compare lanes 3 and 4 or 7 and 8). These results highlight the potential for differential regulation of αKNL2 by distinct SUMO isoforms and SUMO ligases. Although SUMO3-dependent SUMOylation appears to require the conserved sites in αKNL2-C, SUMO1-mediated modification occurs independently of these sites, underscoring the functional diversity of SUMO isoforms in the regulation of αKNL2.

To validate and complement the *in vitro* SUMOylation results, we performed MS analysis to identify the specific lysine residues in αKNL2-C modified by SUMO1 and SUMO3. For this purpose, wild-type αKNL2-C protein was subjected to *in vitro* SUMOylation reactions using either SUMO1(H89K) or SUMO3(H88K) cleavable variants in the presence of the SUMO E3 ligase NSE2. As a negative control, we used the unmodified wild-type αKNL2-C protein incubated under the same conditions without the SUMOylation machinery. The MS analysis revealed that SUMO1 modified multiple lysine residues, including K424, K445, K511, K540, K572, K583, K585, and K598, indicating extensive SUMOylation across the C-terminal region. In contrast, SUMO3-dependent modification was detected only at K424, K511, and K598 (Figure 5C). These findings are consistent with our *in vitro* assay results (Supplemental Figures 7C and 7D), which showed stronger SUMOylation by SUMO1 than SUMO3. Notably, K511, a lysine residue predicted *in silico* (via GPS-SUMO), was confirmed by MS, whereas another predicted site, K474, was not identified, possibly due to technical limitations or preferential modification of other residues *in vitro*. These findings demonstrate that αKNL2 is SUMOylated in a site- and isoform-specific manner, with SUMO3-dependent modification restricted to specific conserved C-terminal lysines.

SUMO conjugation of αKNL2 increases upon ULP1d knockout in *Arabidopsis*

ULP1d, a deSUMOylation enzyme, was identified as an αKNL2 interactor through Y2H screening. To investigate the role of ULP1d in αKNL2 deSUMOylation, we used the previously characterized transfer DNA insertion mutant line *ulp1d-2* (SALK_022798) (Castro et al., 2016). Knockout of the ULP1d gene in homozygous *ulp1d-2* mutants was confirmed via RT-PCR analysis (Supplemental Figure 8A). Homozygous *ulp1d-2* plants exhibited distinct vegetative development phenotypes compared with heterozygous mutants and wild-type plants, consistent with previous reports (Supplemental Figure 8B).

We hypothesized that, if ULP1d mediates the deSUMOylation of αKNL2, the absence of ULP1d would result in increased αKNL2 SUMOylation levels. To test this, we introduced the fusion constructs αKNL2-C-EYFP and αKNL2-C$^{\text{Mut-SUMO}}$-EYFP into the *ulp1d-2* mutant background. Three independent T2 transgenic *Arabidopsis* lines expressing either αKNL2-C-EYFP or αKNL2-C$^{\text{Mut-SUMO}}$-EYFP in the wild-type (Col-0) or *ulp1d-2* background were analyzed. Examination of root tips revealed that centromeric localization of αKNL2-C was abolished in *ulp1d-2* mutants and was primarily confined to the nucleolus, contrasting with its localization in wild-type plants (Figure 6A and Supplemental Figure 8C). This finding underscores the role of SUMOylation in centromeric targeting of αKNL2. In contrast, αKNL2-C$^{\text{Mut-SUMO}}$-EYFP localized predominantly to the nucleoplasm and cytoplasm and occasionally to the nucleolus, mirroring its behavior in the wild-type background (Figure 6A and Supplemental Figure 8C). Transgenic plants expressing αKNL2-C$^{\text{Mut-SUMO}}$-EYFP exhibited defects in vegetative growth, particularly in shoot development, compared with *ulp1d-2 mutants, ulp1d-2* mutants expressing αKNL2-C-EYFP, and wild-type plants (Supplemental Figure 8D). RT-qPCR analysis confirmed similar αKNL2 transcript levels across all three independent lines for both the wild-type and SUMOylation-deficient constructs in wild-type and *ulp1d-2* backgrounds, indicating that the observed phenotypic differences are not due to altered *αKNL2* expression (Supplemental Figure 9A).

Analysis of root tip meristems revealed significant mitotic abnormalities, including misaligned metaphases and mis-segregated chromosomes during anaphase (Figure 6B). The average frequency of mitotic abnormalities was 30%–36% in αKNL2-C$^{\text{Mut-SUMO}}$-EYFP plants and 4%–6% in αKNL2-C-EYFP plants in the *ulp1d-2* background. Similarly, frequencies of 6%–30% were observed in αKNL2-C$^{\text{Mut-SUMO}}$-EYFP plants compared with 0%–1% in αKNL2-C-EYFP plants in the wild-type background. In contrast, minimal or no mitotic abnormalities were observed in *ulp1d-2* (5%) and wild-type plants (0%) (Figure 6C). To quantify αKNL2 SUMOylation levels in the *ulp1d-2* mutant relative to the wild type, total proteins were extracted from plants expressing either αKNL2-C-EYFP or αKNL2-C$^{\text{Mut-SUMO}}$-EYFP. Western blotting with anti-SUMO3 showed increased SUMOylation in the *ulp1d-2* mutants compared to wild-type plants (Supplemental Figure 9B). Furthermore, IP with anti-GFP beads followed by immunoblotting with anti-GFP, anti-SUMO3, and anti-SUMO1 antibodies confirmed a mild accumulation of SUMOylated αKNL2-C in *ulp1d-2*. Interestingly, immunoprecipitated αKNL2-C$^{\text{Mut-SUMO}}$-EYFP samples displayed similar patterns in the wild-type and *ulp1d-2* backgrounds (Figure 6D). These findings demonstrate that ULP1d is a critical protease required for αKNL2 deSUMOylation, which enables the proper centromeric localization of αKNL2 and its functional roles in plant development.

(B and C) Mitotic metaphases and anaphases of αKNL2-C-EYFP and αKNL2-C$^{\text{Mut-SUMO}}$-EYFP in wild-type and *ulp1d-2* plants, showing normal chromosomes **(B)** or misaligned and mis-segregated chromosomes (indicated by white arrows; $n = 30$) **(C)**. Scale bars: 5 μm. Chromosomes were stained with anti-CENH3 (magenta) and anti-tubulin (green), with DAPI as a counterstain.

(D) Quantification of abnormal metaphase and anaphase events from **(B and C)**. A total of 30 cells per line were analyzed, with data from 3 independent lines. Results are shown as mean ± SEM ($n = 3$). Significant differences (indicated by lowercase letters) are based on ANOVA and Tukey's multiple comparisons test ($p < 0.005$).

(E) SUMOylation analysis of αKNL2 in wild-type and *ulp1d-2* mutant plants. Total protein extracts were immunoprecipitated using anti-GFP beads. Immunoprecipitated samples were probed with anti-GFP, anti-SUMO3, or anti-SUMO1. Red and green arrows indicate the MWs of αKNL2-C and αKNL2-C$^{\text{Mut-SUMO}}$, respectively. SUMO conjugates are highlighted by black brackets.

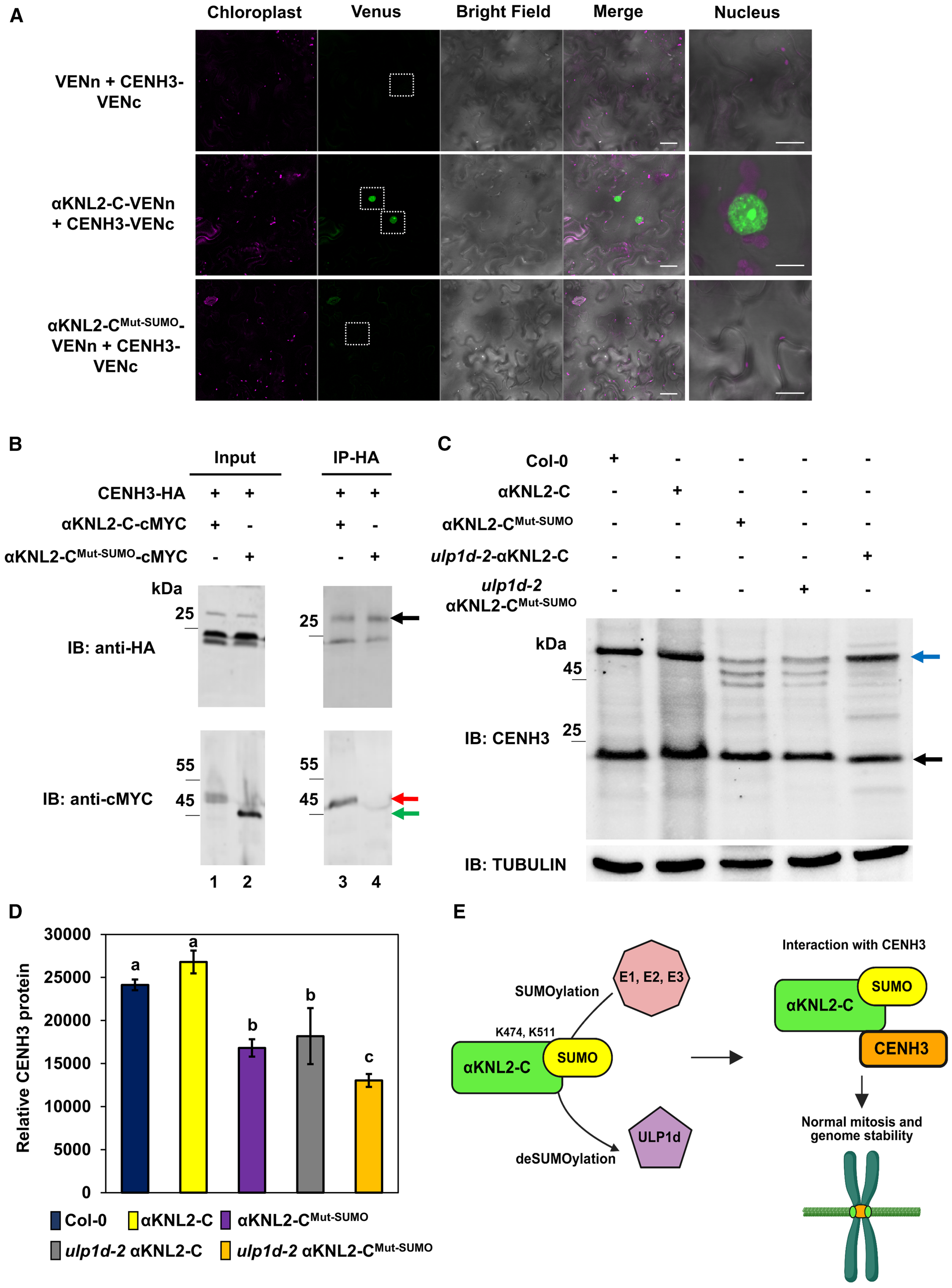

Figure 7. SUMOylation of αKNL2-C is required for its interaction with CENH3.

(A) Confocal microscopy images of *N. benthamiana* leaf epidermal cells transiently expressing CENH3–VENc with either αKNL2-C–VENn, αKNL2-C$^{Mut\text{-}SUMO}$–VENn, or the VENn empty vector for BiFC interaction analysis. Venus fluorescence was detected in the nucleus (white dotted boxes). Scale bars: 50 μm. Right: an enlarged view of the corresponding BiFC signals in the nucleus. Enlarged images may not always correspond to the same nuclei shown in the overview image. Scale bars: 5 μm.

(legend continued on next page)

SUMOylation of αKNL2 is essential for its association with CENH3

Arabidopsis αKNL2 functions as a licensing factor for loading CENH3 at centromeres and is essential for kinetochore assembly. However, direct interaction between αKNL2 and CENH3 in *Arabidopsis* has not previously been demonstrated. In our AP–MS experiment, CENH3 was co-precipitated with αKNL2-C. To validate this association, we performed BiFC and coIP experiments using full-length αKNL2 and its N- and C-terminal regions together with CENH3. The BiFC assay revealed that αKNL2-C^{VENc} specifically interacts with CENH3^{VENn} in the nucleus (Figure 7A). In contrast, neither full-length αKNL2 nor its N-terminal region showed any detectable interaction with CENH3, even after treatment with the proteasome inhibitor MG115 (data not shown). These results demonstrate that αKNL2-C primarily interacts with CENH3.

To investigate the factors influencing αKNL2 interaction with CENH3, we examined the role of αKNL2 SUMOylation and SIM sites using the mutant construct αKNL2-C$^{Mut\text{-}SUMO}$. Unlike wild-type αKNL2-C, this SUMOylation-deficient variant did not interact with CENH3 in the BiFC assay (Figure 7A and Supplemental Figure 10A), indicating that αKNL2-C SUMOylation likely facilitates its interaction with CENH3. Moreover, to confirm the interaction between αKNL2 and CENH3, a Y2H co-transfection assay was performed. Yeast strains expressing αKNL2, αKNL2-N, or αKNL2-C^{BD} as bait and CENH3^{AD} as prey did not grow on selective triple dropout (TDO) medium, indicating that CENH3 may not interact directly with αKNL2. The interaction between CENH3^{AD} and CENH3^{BD} was used as a positive control (Supplemental Figure 10B). This suggests that the interaction may require additional factors or modifications, such as SUMOylation, which are absent in yeast. To further validate the interaction between αKNL2-C and CENH3, a coIP assay was performed. The CENH3^{HA} fusion construct was co-expressed with either αKNL2-C^{cMYC} or a SUMOylation-deficient mutant of αKNL2-C^{cMYC}. Proteins were immunoprecipitated using anti-HA magnetic beads, and subsequent western blotting with an anti-cMYC antibody detected αKNL2-C^{cMYC} co-precipitated with CENH3^{HA}. No interaction was observed between the SUMOylation-deficient αKNL2-C^{cMYC} and CENH3^{HA} (Figure 7B). These findings are consistent with the BiFC results and indicate that the SUMOylation and SIM sites of αKNL2-C are essential for its interaction with CENH3.

Consistent with these observations, western blotting of nuclear protein extracts using anti-CENH3 and anti-αKNL2 antibodies revealed reduced levels of endogenous CENH3 monomer (~19 kDa) and αKNL2 (~75 kDa) in plants expressing the SUMOylation-deficient αKNL2-C mutant in the wild-type background. In the *ulp1d-2* background, αKNL2-C$^{Mut\text{-}SUMO}$ lines exhibited an even greater reduction of CENH3 and αKNL2 proteins relative to αKNL2-C-EYFP plants (Figures 7C and 7D and Supplemental Figure 11A). Furthermore, in wild-type plants, CENH3 was also detected as a ~54 kDa band, likely reflecting its incorporation into stable nucleosomal complexes containing other histones. In contrast, SUMOylation-deficient αKNL2 mutants exhibited additional bands in the ~45–49 kDa range, which may represent unstable CENH3 complexes (Figure 7C). Notably, the transcript levels of both *αKNL2* and *CENH3* remained largely unchanged in these mutants (Supplemental Figures 9A and 11B). This suggests that αKNL2 SUMOylation regulates the protein stability or centromeric deposition of CENH3 and αKNL2 rather than their transcription. Collectively, these findings reveal that SUMOylation of αKNL2 acts as a key regulatory mechanism that promotes its association with CENH3 and facilitates accurate CENH3 deposition and kinetochore assembly in *Arabidopsis*.

DISCUSSION

In *Arabidopsis*, αKNL2 is primarily known for its essential role in kinetochore assembly and CENH3 loading. As a critical regulator of mitosis, αKNL2 is tightly controlled by multifaceted, highly coordinated regulatory mechanisms. Recent studies have begun to uncover the pathways that regulate αKNL2 activity and function in *Arabidopsis* (Yalagapati et al., 2024; Kalidass et al., 2025). Notably, evidence from both animal and plant systems underscores the significance of PTMs in regulating αKNL2 function. In this study, we demonstrated the critical role of SUMOylation in the regulation of αKNL2 function. Functional analyses revealed that the SUMOylation sites in αKNL2 facilitate SUMO conjugation and are indispensable for αKNL2 centromeric localization and kinetochore assembly. Interestingly, expression of the SUMOylation-deficient αKNL2-C variant in the *Arabidopsis* wild-type background resulted in defects in chromosome alignment, growth, and fertility. Therefore, this PTM promotes centromere targeting of αKNL2 and its interaction with CENH3, thereby significantly enhancing αKNL2 protein stability and ensuring proper mitotic progression and cell division (Figure 7E and Supplemental Figure 12).

SUMOylation is a reversible PTM that plays a pivotal role in processes such as the cell cycle, DNA repair, transcription, signal transduction, and chromatin remodeling (Müller et al., 2001; Hay, 2005; van den Berg and Jansen, 2023). Several key cell cycle regulators, including CENP-A (Mérai et al., 2014; Ohkuni et al., 2018; van den Berg et al., 2023), CENP-E (Zhang et al., 2008), Aurora B (Fernández-Miranda et al., 2010), and BUBR1

(B) CoIP interactions between CENH3 and αKNL2-C. *N. benthamiana* leaves were infiltrated with constructs containing CENH3-HA and αKNL2-C-cMYC (lanes 1 and 3) or CENH3-HA and αKNL2-C$^{Mut\text{-}SUMO}$-cMYC (lanes 2 and 4). Total protein extracts were precipitated with anti-HA magnetic beads and probed before (input) and after IP using anti-HA and anti-cMYC antibodies. Black, red, and green arrows indicate the MWs of CENH3, αKNL2-C, and αKNL2-C$^{Mut\text{-}SUMO}$, respectively.

(C) Western blotting of *Arabidopsis* transgenic lines expressing αKNL2-C-EYFP or αKNL2-C$^{Mut\text{-}SUMO}$-EYFP in the Col-0 or *ulp1d-2* background using anti-CENH3. CENH3 monomers and dimers are indicated by black and blue arrows, respectively. Tubulin was used as a loading control.

(D) Quantification of CENH3 monomer levels in the lines shown in **(C)**. Data are presented as mean ± SEM. Significant differences (indicated by lowercase letters) are based on ANOVA and Tukey's multiple comparisons test ($p < 0.005$).

(E) Schemata of the molecular mechanism for SUMOylation-based regulation of αKNL2-C. αKNL2-C is SUMOylated with SUMO1 or SUMO3 at K474 and K511 by the SUMO conjugation machinery (E1, E2, and E3), and this modification is reversed by the SUMO protease ULP1d. Proper SUMO cycling is essential for αKNL2-C function and efficient centromeric loading of CENH3, which ensure normal mitosis and maintain genome stability.

(Yang et al., 2012), have been identified as SUMOylation targets. Our study provides both *in vivo* and *in vitro* evidence that αKNL2 undergoes SUMOylation, predominantly by SUMO3 and SUMO1. In mammals, SUMO1 primarily stabilizes structural proteins, whereas SUMO2 and SUMO3 are involved in the dynamic regulation of protein turnover and interactions. Similarly, in plants, SUMO1 and SUMO2 likely bind key centromeric proteins to regulate their functions during mitosis and meiosis; SUMO3 and SUMO5 may further contribute to the modulation of centromeric protein dynamics, particularly in response to environmental stress (van den Berg and Jansen, 2023).

Our findings indicate that αKNL2 covalently binds to both SUMO1 and SUMO3, pointing to potentially distinct regulatory roles for its SUMOylation by different SUMO isoforms. However, SUMO1 does not interact with αKNL2 in BiFC analysis, likely due to differences in the biochemical and structural properties of SUMO1 and SUMO3. The greater propensity of SUMO3 to form SUMO conjugates may correspond to its stronger binding in BiFC contexts (Chupreta et al., 2005; Castaño-Miquel et al., 2011; Park et al., 2011; Roy and Sadanandom, 2021). Moreover, BiFC requires a stable complex for fluorescence restoration (Miller et al., 2015), and SUMO3 likely forms a stronger or more stable interaction with αKNL2. In contrast, SUMO1–αKNL2 binding may be transient or structurally incompatible with BiFC detection, or SUMO1 may require specific E3 ligases for its recruitment or conjugation. SUMO E3 ligases are crucial for facilitating SUMO conjugation and significantly enhance the efficiency of this process. An *in vitro* assay demonstrated that NSE2 enhances SUMOylation of αKNL2 via SUMO1 modification. Given the canonical role of the SMC5/6 complex and its NSE2 subunit in genome stability maintenance (Aragón, 2018; Palecek, 2018), it is plausible that SUMO1 conjugation to αKNL2 occurs through the involvement of NSE2. Increasing evidence suggests that NSE2 is critical in mitosis through its role as a SUMO ligase for centromeric proteins (Andrews et al., 2005). Therefore, we speculate that NSE2 may function as a specific SUMO E3 ligase for αKNL2. Our future efforts will address this question.

A previous study found that αKNL2 variants fused to EYFP localize to chromocenters and occasionally to the nucleolus and nuclear bodies in *Arabidopsis* and *N. benthamiana* (Lermontova et al., 2013). Protein–protein interaction network analysis revealed that αKNL2 may have multiple roles at the nuclear periphery, including chromatin organization, CENH3 loading, RNA and DNA interactions, nucleotide excision repair, and regulation of nuclear transport. Although the association of αKNL2 with multiple partner proteins suggests roles in distinct subcellular processes, the mechanisms that govern the specificity and regulation of these interactions remain to be elucidated. Using AP–MS, Y2H library screening, and protein interaction assays, we identified SUMO3 and ULP1d as specific interactors of αKNL2-C, suggesting a regulatory mechanism involving SUMOylation and deSUMOylation. This interplay between SUMO3 and ULP1d indicates a dynamic balance of SUMOylation essential for proper αKNL2 function. SUMOylation influences the subcellular localization of proteins by modulating nuclear import/export signals or by promoting interactions with nucleolar targeting sequences (Müller et al., 2001; Wilson and Rangasamy, 2001). SUMOylated proteins often relocalize to nuclear or nucleolar compartments as part of their regulatory roles. Consistent with these observations, the interactions of ULP1d and SUMO3 with αKNL2 were detected in the nucleolus and nuclear bodies, consistent with the occasionally observed localization pattern of αKNL2. Many kinetochore proteins frequently shuttle through the nucleolus to undergo PTMs such as SUMOylation and deSUMOylation, which affect their stability, localization, and interactions. Similarly, in humans, SENP3 and SENP5 are nucleolus-localized enzymes that facilitate modification of several SUMO-2/3 substrates (Gong and Yeh, 2006).

The predictive analysis identified conserved SUMOylation sites and SIM motifs in αKNL2-C, and mutating these residues abolished its centromeric localization and SUMO3 binding. However, single amino acid substitutions did not fully disrupt centromeric localization or SUMO3 binding, suggesting that at least one conserved SUMOylation site must remain functional for proper targeting. Among the evaluated constructs, simultaneous mutations at K474 and K511 caused the most pronounced defects, underscoring their critical role in SUMO-mediated centromere targeting of αKNL2. Consistent with this, K511 (a lysine residue predicted *in silico*) was confirmed by MS, whereas K474 was not detected. The absence of K474 in the MS dataset may reflect technical limitations (such as low stoichiometry, poor peptide detectability, or complex SUMO remnants after digestion) or preferential modification of other lysines under the *in vitro* reaction conditions. It is also possible that K474 SUMOylation depends on cellular factors or structural conformations not fully recapitulated *in vitro*. Moreover, in our *in vivo* AP–MS analysis, native non-cleavable SUMO proteins were present, preventing direct site identification by MS. Importantly, we cannot exclude the possibility that SUMO3 modifications at the C-terminal lysines may create docking platforms for SUMO-targeted ubiquitin ligases, which would suggest SUMO–ubiquitin crosstalk in the regulation of αKNL2 stability and centromere proteostasis. Future studies will explore this potential interplay between SUMOylation and ubiquitin-mediated turnover of αKNL2.

Furthermore, expression of the SUMO/SIM-deficient αKNL2 mutant in *Arabidopsis* wild type causes developmental defects, mitotic abnormalities, and impaired fertility. Nevertheless, pollen viability in the mutants remained unaffected, suggesting that meiosis remained largely intact. The observed fertility defects may instead stem from mitotic abnormalities during gametophyte development or early embryogenesis. These results underscore the importance of PTMs in the regulation of αKNL2 function in chromosome segregation and plant development. Previous studies have also shown that SUMOylation is crucial for the proper function of many centromeric proteins, and that disruption of SUMOylation through mutations at SUMO attachment sites can impair these processes, causing aberrant localization. For instance, in yeast, the assembly of CENP-A/Cse4 is stimulated by C-terminal SUMOylation, and mutations that disrupt SUMOylation of Cse4 can lead to its mislocalization away from centromeres, affecting kinetochore assembly and chromosome segregation (Ohkuni et al., 2018, 2020). SUMOylation-deficient mutants of BubR1 show mislocalization and fail to function properly in spindle checkpoint signaling, leading to defects in chromosome segregation (Yang et al., 2012). In human cells, CENP-E is specifically modified by SUMO-2/3 and binds SUMO-2/3 polymeric chains, a function crucial for its localization to the kinetochore (Zhang et al., 2008).

Identification of ULP1d as a deSUMOylation enzyme for αKNL2 sheds light on the dynamic regulation of deSUMOylation in centromere function. In *ulp1d-2* mutants, SUMOylation of αKNL2-C-EYFP by SUMO1 and SUMO3 was enhanced, which resulted in severe developmental and mitotic defects. Notably, αKNL2-C-EYFP localization in *ulp1d-2* mutants shifted from the centromere to the nucleolus, highlighting the critical role of ULP1d-mediated deSUMOylation in facilitating centromere targeting of αKNL2. Interestingly, localization of the SUMOylation mutant αKNL2-C$^{\text{Mut-SUMO}}$-EYFP in *ulp1d-2* mutants remained unaffected, which suggests that the deSUMOylation sites had already been altered. Similarly, deSUMOylation is mediated by a related family of SUMO-specific proteases, including ULP in yeast and SENP in mammals, and is essential for centromere function. In humans, SENP6 regulates a network of proteins, including the CCAN, the CENP-A loading factors Mis18BP1 and Mis18A, and DNA damage response factors; SENP6 deficiency leads to impaired proliferation, G2/M accumulation, and frequent micronucleus formation (Liebelt et al., 2019). In *Saccharomyces cerevisiae*, Ulp2 is recruited to the kinetochore via the Ctf3$^{\text{CENP-I}}$–Mcm16$^{\text{CENP-H}}$–Mcm22$^{\text{CENP-K}}$ complex and selectively targets CCAN subunits; mutations that impair recruitment or SUMO binding result in increased chromosome loss and hypersensitivity to replication stress (Suhandynata et al., 2019).

Our study demonstrates that αKNL2 SUMOylation is essential for its association with CENH3. The αKNL2–CENH3 interaction was validated through BiFC and coIP assays; however, the Y2H assay suggests that the interaction may not be direct. The absence of interaction in the Y2H assay, despite detection by BiFC and coIP, indicates that αKNL2-C may require additional factors or modifications, such as SUMOylation, that are not present in yeast. Given the necessity of SUMOylation for this interaction, such a modification might influence the conformation of αKNL2-C or facilitate its recruitment by other proteins. Notably, αKNL2-C interacts with CENH3 in *Arabidopsis*, as demonstrated by pull-down and BiFC assays. Consistently, structural studies in chicken have shown that ggKNL2, which contains a CENPC-k motif at its C terminus, specifically recognizes CENP-A/CENH3 nucleosomes through the RG-loop in its C-terminal region (Jiang et al., 2023). In *Caenorhabditis elegans*, the extended N-terminal tail of CENP-A directly interacts with KNL-2, playing a crucial role in chromatin assembly and partially compensating for Scm3/HJURP function (de Groot et al., 2021). These findings suggest that the binding regions may vary across species, necessitating validation in each specific organism.

In plants, the SUMOylation-deficient αKNL2 mutant failed to interact with CENH3, as evidenced by both BiFC and coIP assays. Consistent with this, localization analysis showed that αKNL2-C$^{\text{Mut-SUMO}}$-YFP mainly accumulates in the cytoplasm but also enters the nucleus, indicating that SUMOylation is not required for nuclear import but is essential for the stable association of αKNL2 with CENH3. Previous studies have shown that CENP-C interacts with CENP-A–containing nucleosomes upon CDK1-mediated phosphorylation of CENP-C in human and chicken mitotic cells (Watanabe et al., 2019; Ariyoshi et al., 2021), emphasizing the importance of PTMs in CENP-A/CENH3–kinetochore interactions. Moreover, our analysis of SUMOylation-deficient αKNL2 mutants revealed a marked reduction in the levels of endogenous monomeric CENH3 and αKNL2. In wild-type plant extracts, CENH3 predominantly migrates as a ~54-kDa complex, likely reflecting its incorporation into stable nucleosomal structures with associated histones. However, in SUMOylation-deficient αKNL2 mutants, additional intermediate bands were detected, which likely correspond to partially disassembled CENH3–histone subcomplexes. This suggests that impaired SUMOylation disrupts nucleosome integrity, leading to defective centromeric loading and compromised chromatin stability. These findings suggest that SUMOylation serves as a regulatory mechanism for the αKNL2–CENH3 interaction and CENH3 loading in *Arabidopsis*, a process crucial for centromere assembly and function. Our study reveals that dynamic SUMOylation of αKNL2, regulated by ULP1d, is critical for its centromeric localization, interaction with CENH3, and roles in plant development and mitosis, providing insights into centromere organization and genome stability.

METHODS

Plasmid construction

The complete open reading frames of SUMO1, SUMO2, SUMO3, SUMO5, and ULP1d were amplified via RT-PCR using 1 μg of RNA extracted from flower buds of *A. thaliana* wild type (Columbia-0 ecotype). Primers are listed in Supplemental Table 1. The amplified fragments were cloned into the pDONR221 backbone using the Gateway BP reaction (Invitrogen). The αKNL2, αKNL2-N, and αKNL2-C clones in the pDONR221 vector were generated as described previously (Lermontova et al., 2013). Fragments from the pDONR221 clones were subsequently recombined into Gateway-compatible destination vectors for various applications; the pGWB641 vector, which contains the 35S promoter, was used for *in vivo* subcellular localization studies, whereas the 3′-Venus-N and 3′-Venus-C Gateway vectors, which include cMYC and HA tags under the 35S promoter, were used for BiFC and coIP analyses. For the negative control in coIP experiments, an HA tag with a Venus-C fragment was cloned into the 3′-Venus-C expression vector.

αKNL2-C fragments with simultaneous mutations at three predicted SUMOylation sites and two predicted SUMO interaction motifs were synthesized and cloned into a Gateway-compatible pENTR-TOPO vector by Twist Bioscience (https://www.twistbioscience.com/). Clones with individually deleted SUMOylation sites or SUMO interaction motifs were generated from the αKNL2-C/pDONR221 clone using a site-directed mutagenesis protocol (Phusion Site-Directed Mutagenesis Kit, Thermo Scientific). Mutagenized αKNL2 fragments were recombined from pDONR221 or pENTR-TOPO clones into the Gateway-compatible pGWB641 vector using the Gateway LR reaction (Invitrogen).

The C-terminal fragments of αKNL2 (in pDONR221) and the αKNL2$^{\text{Mut−SUMO}}$ variant (in pTwist-ENTR) were used as templates for cloning into an expression vector suitable for the SUMO *in vitro* assay. Individual fragments were amplified using specific primers listed in Supplemental Table 1. The PCR products were cloned into the BamHI site of the pET-Duet vector, which includes a His tag at the N terminus and a FLAG tag at the C terminus (Adamus et al., 2020), using the NEBuilder HiFi DNA Assembly Kit (New England Biolabs, USA). cDNA from *A. thaliana* was used as a template for cloning NSE2 into the expression vector pET28c+. The PCR product was amplified using specific primers listed in Supplemental Table 1 and subsequently cloned into the BamHI and XhoI sites of the pET28c+ vector, which includes a His tag and T7 tag at the N terminus.

Plant transformation and cultivation

Transient transformation of *N. benthamiana* was carried out using *Agrobacterium tumefaciens* following the protocol described by Walter et al.

(2004). Fluorescence signals were analyzed in the lower epidermal cell layers of tobacco leaves 48 h post-infiltration. Each expression plasmid was introduced into *N. benthamiana* in at least three independent infiltration experiments.

For stable transformation of *A. thaliana*, plasmids were transferred into *A. tumefaciens* strain GV3101 via electroporation. Transformation of *A. thaliana* (Col-0) was performed using the floral dip method (Clough and Bent, 1998). Transgenic lines were subsequently generated, and the morphology and GFP signals resulting from plasmid-mediated gene expression were analyzed in at least three independent single-insertion lines.

A. thaliana and *N. benthamiana* plants used for localization studies, nuclei extraction, and BiFC analysis were grown under the following temperature conditions: *A. thaliana* was cultivated under a 16-h light/8-h dark photoperiod with day/night temperatures of 20°C/18°C, whereas *N. benthamiana* was grown under a 12-h photoperiod at a constant temperature of 26°C.

Bimolecular fluorescence complementation (BiFC) assay

To visualize protein interactions *in vivo*, BiFC was performed following the protocol of Yadala et al. (2022). Leaves of 2- to 4-week-old *N. benthamiana* plants were co-infiltrated with *A. tumefaciens* strain GV3101 containing two BiFC vectors expressing the proteins of interest. HC-Pro, an RNA silencing suppressor, was co-expressed to enhance transient expression (Kasschau et al., 2003). Each BiFC combination was tested in at least three independent infiltration experiments using three different plants ($n = 3$ biological replicates).

Yeast-two hybrid (Y2H) co-transformation assay

Y2H assays were performed to assess the interaction between two selected proteins. The cDNAs encoding the proteins of interest were cloned into the pGADT7 and pGBKT7 vectors and co-transformed into the *S. cerevisiae* Y2H Gold strain, following the Matchmaker Gold Y2H System protocol (Takara Bio, catalog number 630489). Transformed yeast cells were plated on –LT (SD/−Leu/−Trp) selective medium and incubated at 30°C for 3–5 days. Colonies were subsequently picked, serially diluted (1/10, 1/100, and 1/1000), and spotted onto –LT and –LTH (SD/−Leu/−Trp/−His) plates to evaluate interaction-dependent growth. All co-transformations and interaction assays were performed in at least two independent experiments.

Protein extraction, immunoprecipitation (IP), and immunoblot (IB) analyses

Total protein extracts were obtained from *Arabidopsis* seedlings expressing αKNL2-C or its SUMOylation site mutants fused to EYFP using a previously described phenol extraction method (Hurkman and Tanaka, 1986). The nuclear protein extracts were isolated according to the protocol of Huang et al. (2021). Protein extraction was performed in triplicate for each genotype or treatment across 3 independent experiments. IP and coIP were conducted using GFP- and HA-trap kits (Chromotek) according to the manufacturer's instructions. The samples were then used for immunoblotting analyses.

For immunoblot analyses, 2× SDS sample buffer (125 mM Tris-HCl [pH 6.8], 4% SDS, 20% glycerol, 10% β-mercaptoethanol, and 0.02% bromophenol blue) was added to each protein sample, followed by boiling for 10 min. The protein samples were separated by SDS-PAGE using a 10% acrylamide gel and transferred to a polyvinylidene fluoride membrane (Thermo Scientific) via electroblotting. Membranes were blocked for 1 h at room temperature in PBS containing 5% (w/v) low-fat milk powder and then incubated overnight at 4°C with primary antibodies diluted in 1% BSA/PBS: rabbit anti-SUMO1 (1:1000; Abcam), rabbit anti-SUMO3 (1:1000; Abcam), mouse anti-GFP (1:1000; JL8, Living Colors), mouse anti-HA (1:10 000; Proteintech), mouse anti-cMYC (1:500), mouse anti-tubulin (1:1000; Sigma, T9026), rabbit anti-CENH3 (1:1000; Abcam, ab72001), or rabbit anti-αKNL2 (1:1000; LifeTein, rb115). The membranes were then incubated with secondary anti-mouse or anti-rabbit antibodies (1:5000) conjugated to IRDye 800CW and visualized using an LI-COR Biosciences Odyssey scanner. Signal intensities were quantified using Image Studio (version 3.1, LI-COR Biosciences).

Protein purification

Expression vectors encoding the enzymes of the SUMO machinery (His-SUMO1 [AT4G26840]; His-SUMO3 [AT5G55170]; untagged SCE1 [AT3G57870]; and SAE, consisting of the smaller His-tagged subunit SAE1b [AT5G50680] and the larger subunit SAE2 [AT2G21470]) were kindly provided by the laboratory of Prof. Andreas Bachmair (Tomanov et al., 2022).

His-tagged *Arabidopsis* SUMOylation enzymes and αKNL2 proteins (wild-type and SUMOylation-deficient variants) were expressed in *Escherichia coli* BL21(DE3)RIL cells. Transformed cells were grown in Lysogeny Broth (LB) medium at 37°C to an optical density of 0.5 at 600 nm. Protein expression was induced with 1 mM isopropyl β-D-1-thiogalactopyranoside (IPTG) at 37°C for 3 h. Cells were pelleted and resuspended in lysis/binding buffer (50 mM phosphate buffer [pH 8.0], 300 mM NaCl, 10 mM imidazole, 10% glycerol, and 0.5% Triton X-100), followed by sonication. The lysate was cleared by centrifugation, and the supernatant was incubated with TALON His-affinity resin (Clontech, USA) for 1.5 h at 4°C. The resin was applied to a gravity-flow column, washed with wash buffer (50 mM phosphate buffer [pH 8.0], 300 mM NaCl, and 20 mM imidazole), and eluted with 250 mM imidazole. Elution fractions were analyzed using SDS–PAGE and Coomassie staining. Fractions containing the target protein were pooled and concentrated using Amicon Ultra centrifugal filter units (Molecular Weight Cut-Off [MWCO]; Merck Millipore, USA). The concentrated fractions were aliquoted, and protein concentration was determined by SDS–PAGE with a BSA standard.

The SUMO E3 ligase NSE2 was expressed in Lysogeny Broth medium at 37°C until the optical density at 600 nm reached 0.5. Expression was induced using 0.5 mM IPTG at 30°C for 3 h. Cells were pelleted and resuspended in the same lysis/binding buffer as above, with the addition of 0.5 mM Tris(2-carboxyethyl)phosphine (TCEP). The remaining purification steps were identical to those described above. For elution, 350 mM imidazole was used, as better yields were obtained under these conditions. The untagged SCE enzyme was expressed and purified in a similar manner. Expression was induced using 1 mM IPTG at 20°C for 1 h. After centrifugation (4500 *g*, 4°C, 20 min), the pellet was resuspended in SUMO buffer (20 mM Tris–HCl [pH 7.4], 5 mM $MgCl_2$, and 0.5 mM Tris(2-carboxyethyl)phosphine). Lysates were sonicated, and protein concentrations were determined using SDS–PAGE with a BSA standard.

In vitro SUMO assay

For the *in vitro* SUMO assay, a protocol similar to that of Tomanov et al. (2022) was used. The SUMOylation reaction mixture contained 2 μM SAE, 1.75 μM SCE1, 14 μM SUMO3 or SUMO1, 2 μM αKNL2 protein, 7 μM NSE2 SUMO E3 ligase, 10× SUMO buffer, and 5 mM ATP. The reaction volume was adjusted to 20 μl with water. The mixture was incubated at 30°C for 2 h. After incubation, 20 μl of 2× Laemmli sample buffer was added, and samples were heated at 95°C for 5 min. Samples (20 μl) were separated on 12% SDS-PAGE gels, transferred to nitrocellulose membranes, reversibly stained with Ponceau S, and analyzed by immunoblotting with an anti-FLAG horseradish peroxidase (HRP)-conjugated antibody (1:3000; Abcam, A8592).

Mass spectrometry (MS) analysis of αKNL2 SUMOylation *in vitro*

For MS-based identification of SUMOylation sites, the same enzymatic system described above for the *in vitro* SUMO assay was scaled up to a final volume of 100 μl, and SUMO proteins were substituted with cleavable

SUMO1(H89K) or SUMO3(H88K) variants. After 2 h of incubation at 30°C, the reaction mixture was added to 30 μl of equilibrated anti-FLAG magnetic beads (Sigma-Aldrich) and incubated at 4°C with gentle rotation. The beads were washed several times with buffer lacking imidazole to remove unbound proteins and contaminants. Following the IP washes, bead-bound protein complexes were reduced using 25 mM DTT (30 min at 56°C), alkylated using 100 mM iodoacetamide (20 min at room temperature in the dark), quenched using 75 mM DTT (20 min at room temperature), and digested directly on the beads using LysC (0.2 μg, Promega) in 50 mM $NaHCO_{3\ buffer}$ for 2 h at 37°C. The beads were removed from the initial digestion reaction, and a second digestion step was performed using trypsin (0.5 μg; sequencing grade, Promega) for 18 h at 37°C. The resulting peptides were extracted using 2.5% formic acid (FA) in 50% acetonitrile (ACN) and 100% ACN supplemented with n-dodecyl-β-D-maltoside (final concentration 0.1%; Sigma-Aldrich), transferred to liquid chromatography–MS vials, and concentrated in a SpeedVac concentrator (Thermo Fisher Scientific).

Liquid chromatography–MS/MS analyses of peptide solutions were performed using the UltiMate 3000 RSLCnano system (Thermo Fisher Scientific) connected to a timsTOF Pro or Pro 2 mass spectrometer (Bruker). Before LC separation, tryptic digests were concentrated and desalted on-line using a trapping column (Acclaim PepMap 100 C18, 300 μm inner diameter (ID), 5 mm long, 5 μm particles; Thermo Fisher Scientific). After washing the trapping column with 0.1% trifluoroacetic acid, the peptides were eluted onto an analytical column (Aurora C18, 75 μm inner diameter, 250 mm long, 1.7 μm particles, heated to 50°C; PN AUR3-25075C18-CSI, Ion Opticks) at a flow rate of 150 nl/min using a 60-min linear gradient (3%–42% mobile phase B; mobile phase A, 0.1% FA in water; mobile phase B, 0.1% FA in 80% ACN). Columns were equilibrated before sample injection into the sample loop. The analytical column was placed inside the Column Toaster heater (Bruker), and its emitter side was connected to the CaptiveSpray ion source (Bruker) according to the manufacturer's instructions. The column temperature was set to 50°C, and a spray voltage of 1.4 kV was used. Parallel accumulation-serial fragmentation (PASEF) data denoising was switched off. MS data were acquired over an *m/z* range of 100–1700 and a $1/K_0$ range of 0.6–1.4 $V \times s \times cm^{-2}$ using the data-dependent acquisition-parallel accumulation–serial fragmentation (DDA-PASEF) method; 10 PASEF scans were acquired with a scheduled target intensity of 20 000 and an intensity threshold of 2500. Active exclusion was set to 0.4 min, with precursor reconsideration enabled for signals at least 4× more intense.

Raw MS data were processed using DataAnalysis software (version 6.1), and MS2 spectra were exported in Mascot generic format (MGF). The MS2 spectra were searched using Proteome Discoverer (v1.4; Thermo Fisher Scientific) and an in-house Mascot server (v2.6.2) in a two-step workflow. First, the data were searched against a modified common Repository of Adventitious Proteins (cRAP) database (based on https://www.thegpm.org/crap/) using the following parameters: precursor and fragment tolerance of 10 ppm and 0.03 Da, respectively; full tryptic/P specificity with up to 2 missed cleavages allowed; oxidation (M), deamidation (NQ), and N-terminal acetylation as variable modifications; and carbamidomethyl (C) as a fixed modification. The peptide cutoff score was set to 30. Following the cRAP database search, MS2 spectra assigned to any protein in the cRAP database with a Mascot ion score of 30 or higher were removed, and the remaining spectra were searched against a custom database containing proteins of interest. The same search parameters were used with the following modifications: diglycine on lysine QTGG(K) was added as a variable modification, up to 3 missed cleavages were allowed, and a peptide cutoff score of 20 was used. Search results were manually inspected in Proteome Discoverer and DataAnalysis (for extracted ion chromatograms), considering all acquired data, especially the following characteristics: fragment assignment quality, mass error, Mascot ion score, and extracted ion chromatograms of QTGG(K)-modified peptides. Spectra matched to SUMOylated peptides of interest were checked against the results from a parallel search against the full *A. thaliana* proteome (https://ftp.uniprot.org/pub/databases/uniprot/current_release/knowledgebase/reference_proteomes/Eukaryota/UP000006548/UP000006548_3702.fasta.gz; June 19, 2025; 27 448 protein sequences) using the same parameters as those applied to the dedicated protein database.

RNA isolation and RT-qPCR analysis

Total RNA was extracted from 7-day-old seedlings using TRIzol reagent and treated with DNase to remove genomic DNA contamination. First-strand cDNA synthesis was performed using the Genaxxon Scriptase RT cDNA synthesis kit with an oligo(dT)18 primer and 2 μg of total RNA as input. RT-qPCR was carried out on an Applied Biosystems QuantStudio 6 Flex system using Genaxxon SYBR Green Supermix. Each transcript was analyzed in triplicate across three independent biological replicates. *ACTIN* and *UBQ* were used as internal reference genes for normalization. PCR reactions (10 μl) were set up to amplify ACTIN, UBQ, αKNL2-C, and CENH3. The thermal cycling conditions consisted of initial denaturation at 95°C for 5 min, followed by 40 cycles of 15 s at 95°C, 30 s at 62°C for annealing, and 30 s at 72°C for elongation.

Immunostaining

Samples were prepared from *N. benthamiana* leaves infiltrated with a plasmid expressing the protein of interest tagged with EYFP. Nuclei were extracted from infiltrated leaves (Doležel et al., 2007). This preparation preserved EYFP fluorescence, allowing co-localization analysis via immunostaining with an antibody specific to *N. benthamiana* CENH3. For *A. thaliana*, nuclei and chromosomes were prepared for mitotic analysis following Lermontova et al. (2006). Immunostaining of nuclei and chromosomes was conducted as outlined by Jasencakova et al. (2000). Primary antibodies used included rabbit anti-CENH3 (1:1000; LifeTein, rb5558), mouse anti-GFP (1:1000; Chromotek, 5F8), and mouse anti-tubulin (1:1000; Sigma, T9026). Secondary antibodies included rhodamine-conjugated anti-rabbit (1:300; Jackson ImmunoResearch Laboratories) and Alexa 488–conjugated anti-mouse (1:300; Jackson ImmunoResearch Laboratories). Counterstaining was performed with DAPI (Vector Laboratories, USA).

Microscopy

EYFP fluorescence detection was performed on transformed *N. benthamiana* leaves and *A. thaliana* seedlings using a confocal laser scanning microscope (LSM 780, Carl Zeiss). EYFP was excited with a 488-nm laser, and fluorescence was captured using a 505–550 nm band-pass filter. Spatial super-resolution structured illumination microscopy was performed using a Plan-Apochromat 63×/1.4 oil objective with an Elyra PS.1 microscope system and ZENblack software (Carl Zeiss) (Weisshart et al., 2016).

Seed set and plant fertility evaluation

For seed set analysis, siliques were fixed in ethanol:acetic acid (9:1) overnight and then dehydrated in 70% and 90% ethanol for 1 h each. Samples were cleared overnight at 4°C in chloral hydrate solution (chloral hydrate:water:glycerol, 8:2:1). Seeds within siliques were counted under a binocular microscope (Carl Zeiss, Germany).

To evaluate plant fertility in αKNL2 SUMOylation mutant lines, scanning electron microscopy was used. Fresh siliques were fixed using 4% formaldehyde in 50 mM phosphate buffer (pH 7.0) for 16 h at 8°C. Samples were washed briefly with distilled water and dehydrated in an ascending ethanol series (30%, 50%, 70%, 90%, and 100%) twice each. Critical point drying was performed using a Quorum K850 critical point dryer (Quorum Technologies). Dried samples were mounted on carbon adhesive discs, gold coated using an Edwards S150B sputter coater, and imaged with a Zeiss Gemini300 scanning electron microscope (Carl Zeiss Microscopy) at 5 kV acceleration voltage. Images were saved as Tagged Image File Format (TIFF) files.

Bioinformatic analysis

Protein interaction networks and Gene Ontology analyses were performed using Cytoscape v.3.8.2 (https://cytoscape.org/) and STRING (https://string-db.org/). SUMOylation sites in αKNL2 were identified using the GPS-SUMO web tool (https://sumo.biocuckoo.cn/).

Quantification and statistical analysis

Primary root lengths, silique lengths, and BiFC fluorescence intensity were quantified using ImageJ software. Data are presented as mean ± SEM based on at least three independent experiments or biological samples. For pairwise comparisons, Welch's *t*-test was used to account for unequal variances, using the T.TEST function in Excel (two-tailed, unequal variance). Comparisons involving more than two groups were analyzed in R using one-way ANOVA followed by Tukey's *post hoc* multiple comparisons test. Statistical significance was defined as $^{**}p < 0.05$ and $^{***}p < 0.005$ for all tests. All statistical outputs are provided in Supplemental Data Set 1.

DATA AND CODE AVAILABILITY

The MS proteomics data have been deposited to the ProteomeXchange Consortium via the PRIDE partner repository (Perez-Riverol et al., 2025) under the dataset identifier PXD067383 (https://doi.org/10.6019/PXD067383).

FUNDING

This work was supported by Deutsche Forschungsgemeinschaft (DFG) grant DFG LE 2299/5-1. J.V. acknowledges support from ProteoCure COST (European Cooperation in Science and Technology) Action CA20113, which funded the research stay during which the methodology applied in this publication was acquired. J.R.C. was supported by DFG grant DFG LE 2299/8-1. J.J.P. was supported by the Czech Science Foundation (GA23-05284S). The CEITEC Proteomics Core Facility of CIISB, Instruct-CZ Centre, was supported by MEYS CR (LM2023042, CZ.02.01.01/00/23_015/0008175, and e-INFRA CZ [ID: 90 254]).

ACKNOWLEDGMENTS

We thank Prof. Dr. Andreas Bachmair for providing plasmids for the SUMO *in vitro* assay, Dr. Eva Dvořák Tomaštíková for providing anti-SUMO1 and anti-SUMO3 antibodies, Dr. Twan Rutten for scanning electron microscopy analysis, and Heike Kuhlmann and Annette Heber for technical assistance. No conflict of interest is declared.

AUTHOR CONTRIBUTIONS

M.K. and I.L. conceived the study and designed the experiments. M.K., J.V., V.G.J., and S.D.K.Ö. performed plasmid construction, cloning, mutagenesis, localization, and protein interaction studies. M.K. and D.D. conducted the western blot analyses. M.K. and J.R.C. performed immunostaining, RT-qPCR, and mitotic analyses. J.V., B.K., D.P., and J.J.P. conceived and performed the *in vitro* SUMOylation assay and MS analysis. M.K. and V.S. performed microscopy analysis. M.K. and I.L. wrote the manuscript with input from all co-authors. I.L. supervised the study and secured funding. All authors reviewed the manuscript.

SUPPLEMENTAL INFORMATION

Supplemental information is available at *Plant Communications Online*.

Received: April 26, 2025
Revised: August 24, 2025
Accepted: November 15, 2025
Published: November 19, 2025

REFERENCES

Adamus, M., Lelkes, E., Potesil, D., Ganji, S.R., Kolesár, P., Zábrady, K., Zdráhal, Z., and Palecek, J.J. (2020). Molecular insights into the architecture of the human SMC5/6 complex. J. Mol. Biol. **432**:3820–3837.

Andrews, E.A., Palecek, J., Sergeant, J., Taylor, E., Lehmann, A.R., and Watts, F.Z. (2005). Nse2, a component of the Smc5-6 complex, is a SUMO ligase required for the response to DNA damage. Mol. Cell Biol. **25**:185–196.

Aragón, L. (2018). The Smc5/6 complex: new and old functions of the enigmatic long-distance relative. Annu. Rev. Genet. **52**:89–107.

Ariyoshi, M., Makino, F., Watanabe, R., Nakagawa, R., Kato, T., Namba, K., Arimura, Y., Fujita, R., Kurumizaka, H., Okumura, E.i., et al. (2021). Cryo-EM structure of the CENP-A nucleosome in complex with phosphorylated CENP-C. EMBO J. **40**:e105671.

Azuma, Y., Arnaoutov, A., and Dasso, M. (2003). SUMO-2/3 regulates topoisomerase II in mitosis. J. Cell Biol. **163**:477–487.

Bailey, M., Srivastava, A., Conti, L., Nelis, S., Zhang, C., Florance, H., Love, A., Milner, J., Napier, R., Grant, M., et al. (2016). Stability of small ubiquitin-like modifier (SUMO) proteases OVERLY TOLERANT TO SALT1 and-2 modulates salicylic acid signalling and SUMO1/2 conjugation in Arabidopsis thaliana. J. Exp. Bot. **67**:353–363.

Ban, R., Nishida, T., and Urano, T. (2011). Mitotic kinase Aurora-B is regulated by SUMO-2/3 conjugation/deconjugation during mitosis. Genes Cells **16**:652–669.

Castaño-Miquel, L., Seguí, J., and Lois, L.M. (2011). Distinctive properties of Arabidopsis SUMO paralogues support the in vivo predominant role of AtSUMO1/2 isoforms. Biochem. J. **436**:581–590.

Castro, P.H., Couto, D., Freitas, S., Verde, N., Macho, A.P., Huguet, S., Botella, M.A., Ruiz-Albert, J., Tavares, R.M., Bejarano, E.R., et al. (2016). SUMO proteases ULP1c and ULP1d are required for development and osmotic stress responses in Arabidopsis thaliana. Plant Mol. Biol. **92**:143–159.

Chosed, R., Mukherjee, S., Lois, L.M., and Orth, K. (2006). Evolution of a signalling system that incorporates both redundancy and diversity: Arabidopsis SUMOylation. Biochem. J. **398**:521–529.

Chupreta, S., Holmstrom, S., Subramanian, L., and Iñiguez-Lluhí, J.A. (2005). A small conserved surface in SUMO is the critical structural determinant of its transcriptional inhibitory properties. Mol. Cell. Biol. **25**:4272–4282.

Clough, S.J., and Bent, A.F. (1998). Floral dip: a simplified method for Agrobacterium-mediated transformation of Arabidopsis thaliana. Plant J. **16**:735–743.

Colby, T., Matthäi, A., Boeckelmann, A., and Stuible, H.-P. (2006). SUMO-conjugating and SUMO-deconjugating enzymes from Arabidopsis. Plant Physiol. **142**:318–332.

Conti, L., Nelis, S., Zhang, C., Woodcock, A., Swarup, R., Galbiati, M., Tonelli, C., Napier, R., Hedden, P., Bennett, M., et al. (2014). Small ubiquitin-like modifier protein SUMO enables plants to control growth independently of the phytohormone gibberellin. Dev. Cell **28**:102–110.

Conti, L., Price, G., O'Donnell, E., Schwessinger, B., Dominy, P., and Sadanandom, A. (2008). Small ubiquitin-like modifier proteases OVERLY TOLERANT TO SALT1 and-2 regulate salt stress responses in Arabidopsis. Plant Cell **20**:2894–2908.

Cubeñas-Potts, C., Goeres, J.D., and Matunis, M.J. (2013). SENP1 and SENP2 affect spatial and temporal control of sumoylation in mitosis. Mol. Biol. Cell **24**:3483–3495.

Cuijpers, S.A.G., Willemstein, E., and Vertegaal, A.C.O. (2017). Converging small ubiquitin-like modifier (SUMO) and ubiquitin signaling: improved methodology identifies co-modified target proteins. Mol. Cell. Proteomics **16**:2281–2295.

de Groot, C., Houston, J., Davis, B., Gerson-Gurwitz, A., Monen, J., Lara-Gonzalez, P., Oegema, K., Shiau, A.K., and Desai, A. (2021). The N-terminal tail of C. elegans CENP-A interacts with KNL-2 and is essential for centromeric chromatin assembly. Mol. Biol. Cell **32**:1193–1201.

Doležel, J., Greilhuber, J., and Suda, J. (2007). Estimation of nuclear DNA content in plants using flow cytometry. Nat. Protoc. **2**:2233–2244.

Fernández-Miranda, G., Pérez de Castro, I., Carmena, M., Aguirre-Portolés, C., Ruchaud, S., Fant, X., Montoya, G., Earnshaw, W.C., and Malumbres, M. (2010). SUMOylation modulates the function of Aurora-B kinase. J. Cell Sci. **123**:2823–2833.

French, B.T., Westhorpe, F.G., Limouse, C., and Straight, A.F. (2017). *Xenopus laevis* M18BP1 directly binds existing CENP-A nucleosomes to promote centromeric chromatin assembly. Dev. Cell **42**:190–199.e10.

Fu, H., Liu, N., Dong, Q., Ma, C., Yang, J., Xiong, J., Zhang, Z., Qi, X., Huang, C., and Zhu, B. (2019). SENP6-mediated M18BP1 deSUMOylation regulates CENP-A centromeric localization. Cell Res. **29**:254–257.

Gong, L., and Yeh, E.T.H. (2006). Characterization of a family of nucleolar SUMO-specific proteases with preference for SUMO-2 or SUMO-3. J. Biol. Chem. **281**:15869–15877.

Hay, R.T. (2005). SUMO: a history of modification. Mol. Cell **18**:1–12.

Hermkes, R., Fu, Y.-F., Nürrenberg, K., Budhiraja, R., Schmelzer, E., Elrouby, N., Dohmen, R.J., Bachmair, A., and Coupland, G. (2011). Distinct roles for Arabidopsis SUMO protease ESD4 and its closest homolog ELS1. Planta **233**:63–73.

Hori, T., Shang, W.H., Hara, M., Ariyoshi, M., Arimura, Y., Fujita, R., Kurumizaka, H., and Fukagawa, T. (2017). Association of M18BP1/KNL2 with CENP-A Nucleosome Is Essential for Centromere Formation in Non-mammalian Vertebrates. Dev. Cell **42**:181–189.e3.

Huang, S., Zhu, S., Kumar, P., and MacMicking, J.D. (2021). A phase-separated nuclear GBPL circuit controls immunity in plants. Nature **594**:424–429.

Hurkman, W.J., and Tanaka, C.K. (1986). Solubilization of plant membrane proteins for analysis by two-dimensional gel electrophoresis. Plant Physiol. **81**:802–806.

Jasencakova, Z., Meister, A., Walter, J., Turner, B.M., and Schubert, I. (2000). Histone H4 acetylation of euchromatin and heterochromatin is cell cycle dependent and correlated with replication rather than with transcription. Plant Cell **12**:2087–2100.

Jiang, H., Ariyoshi, M., Hori, T., Watanabe, R., Makino, F., Namba, K., and Fukagawa, T. (2023). The cryo-EM structure of the CENP-A nucleosome in complex with ggKNL2. EMBO J. **42**:e111965.

Kalidass, M., Jarubula, V.G., Ratnikava, M., Chandra, J.R., Le Goff, S., Probst, A.V., Esposito, S., Grasser, K.D., Bruckmann, A., Gagneux, J.F., et al. (2025). Ubiquitin-dependent proteolysis of KNL2 driven by APC/CCDC20 is critical for centromere integrity and mitotic fidelity. Plant Cell **37**:koaf164.

Kasschau, K.D., Xie, Z., Allen, E., Llave, C., Chapman, E.J., Krizan, K.A., and Carrington, J.C. (2003). P1/HC-Pro, a Viral Suppressor of RNA Silencing, Interferes with Arabidopsis Development and miRNA Function. Dev. Cell **4**:205–217.

Lermontova, I., Kuhlmann, M., Friedel, S., Rutten, T., Heckmann, S., Sandmann, M., Demidov, D., Schubert, V., and Schubert, I. (2013). Arabidopsis kinetochore null2 is an upstream component for centromeric histone H3 variant cenH3 deposition at centromeres. Plant Cell **25**:3389–3404.

Lermontova, I., Schubert, V., Fuchs, J., Klatte, S., Macas, J., and Schubert, I. (2006). Loading of Arabidopsis centromeric histone CENH3 occurs mainly during G2 and requires the presence of the histone fold domain. Plant Cell **18**:2443–2451.

Li, T., Chen, L., Cheng, J., Dai, J., Huang, Y., Zhang, J., Liu, Z., Li, A., Li, N., Wang, H., et al. (2016). SUMOylated NKAP is essential for chromosome alignment by anchoring CENP-E to kinetochores. Nat. Commun. **7**:12969.

Liebelt, F., Jansen, N.S., Kumar, S., Gracheva, E., Claessens, L.A., Verlaan-de Vries, M., Willemstein, E., and Vertegaal, A.C.O. (2019). The poly-SUMO2/3 protease SENP6 enables assembly of the constitutive centromere-associated network by group deSUMOylation. Nat. Commun. **10**:3987.

Mahajan, R., Gerace, L., and Melchior, F. (1998). Molecular characterization of the SUMO-1 modification of RanGAP1 and its role in nuclear envelope association. J. Cell Biol. **140**:259–270.

Matunis, M.J., Wu, J., and Blobel, G. (1998). SUMO-1 modification and its role in targeting the Ran GTPase-activating protein, RanGAP1, to the nuclear pore complex. J. Cell Biol. **140**:499–509.

Mérai, Z., Chumak, N., García-Aguilar, M., Hsieh, T.-F., Nishimura, T., Schoft, V.K., Bindics, J., Ślusarz, L., Arnoux, S., and Opravil, S. (2014). The AAA-ATPase molecular chaperone Cdc48/p97 disassembles sumoylated centromeres, decondenses heterochromatin, and activates ribosomal RNA genes. Proc. Natl. Acad. Sci. **111**:16166–16171.

Miller, K.E., Kim, Y., Huh, W.-K., and Park, H.-O. (2015). Bimolecular fluorescence complementation (BiFC) analysis: advances and recent applications for genome-wide interaction studies. J. Mol. Biol. **427**:2039–2055.

Montpetit, B., Hazbun, T.R., Fields, S., and Hieter, P. (2006). Sumoylation of the budding yeast kinetochore protein Ndc10 is required for Ndc10 spindle localization and regulation of anaphase spindle elongation. J. Cell Biol. **174**:653–663.

Mukhopadhyay, D., Arnaoutov, A., and Dasso, M. (2010). The SUMO protease SENP6 is essential for inner kinetochore assembly. J. Cell Biol. **188**:681–692.

Müller, S., Hoege, C., Pyrowolakis, G., and Jentsch, S. (2001). SUMO, ubiquitin's mysterious cousin. Nat. Rev. Mol. Cell Biol. **2**:202–210.

Murtas, G., Reeves, P.H., Fu, Y.-F., Bancroft, I., Dean, C., and Coupland, G. (2003). A nuclear protease required for flowering-time regulation in Arabidopsis reduces the abundance of SMALL UBIQUITIN-RELATED MODIFIER conjugates. Plant Cell **15**:2308–2319.

Naish, M., and Henderson, I.R. (2024). The structure, function, and evolution of plant centromeres. Genome Res. **34**:161–178.

Ohkuni, K., Levy-Myers, R., Warren, J., Au, W.-C., Takahashi, Y., Baker, R.E., and Basrai, M.A. (2018). N-terminal sumoylation of centromeric histone H3 variant Cse4 regulates its proteolysis to prevent mislocalization to non-centromeric chromatin. G3 (Bethesda) **8**:1215–1223.

Ohkuni, K., Suva, E., Au, W.-C., Walker, R.L., Levy-Myers, R., Meltzer, P.S., Baker, R.E., and Basrai, M.A. (2020). Deposition of centromeric histone H3 variant CENP-A/Cse4 into chromatin is facilitated by its C-terminal sumoylation. Genetics **214**:839–854.

Palecek, J.J. (2018). SMC5/6: multifunctional player in replication. Genes **10**:7.

Park, H.J., Kim, W.-Y., Park, H.C., Lee, S.Y., Bohnert, H.J., and Yun, D.-J. (2011). SUMO and SUMOylation in plants. Mol. Cells **32**:305–316.

Perez-Riverol, Y., Bandla, C., Kundu, D.J., Kamatchinathan, S., Bai, J., Hewapathirana, S., John, N.S., Prakash, A., Walzer, M., Wang, S., et al. (2025). The PRIDE database at 20 years: 2025 update. Nucleic Acids Res. **53**:D543–D553.

Pichler, A., Knipscheer, P., Oberhofer, E., Van Dijk, W.J., Körner, R., Olsen, J.V., Jentsch, S., Melchior, F., and Sixma, T.K. (2005). SUMO modification of the ubiquitin-conjugating enzyme E2-25K. Nat. Struct. Mol. Biol. **12**:264–269.

Roy, D., and Sadanandom, A. (2021). SUMO mediated regulation of transcription factors as a mechanism for transducing environmental cues into cellular signaling in plants. Cell. Mol. Life Sci. **78**:2641–2664.

Sadanandom, A., Ádám, É., Orosa, B., Viczián, A., Klose, C., Zhang, C., Josse, E.-M., Kozma-Bognár, L., and Nagy, F. (2015).

SUMOylation of phytochrome-B negatively regulates light-induced signaling in Arabidopsis thaliana. Proc. Natl. Acad. Sci. **112**:11108–11113.

Sandmann, M., Talbert, P., Demidov, D., Kuhlmann, M., Rutten, T., Conrad, U., and Lermontova, I. (2017). Targeting of Arabidopsis KNL2 to Centromeres Depends on the Conserved CENPC-k Motif in Its C Terminus. Plant Cell **29**:144–155.

Subramonian, D., Chen, T.-A., Paolini, N., and Zhang, X.-D.D. (2021). Poly-SUMO-2/3 chain modification of Nuf2 facilitates CENP-E kinetochore localization and chromosome congression during mitosis. Cell Cycle **20**:855–873.

Suhandynata, R.T., Quan, Y., Yang, Y., Yuan, W.-T., Albuquerque, C.P., and Zhou, H. (2019). Recruitment of the Ulp2 protease to the inner kinetochore prevents its hyper-sumoylation to ensure accurate chromosome segregation. PLoS Genet. **15**:e1008477.

Talbert, P.B., Masuelli, R., Tyagi, A.P., Comai, L., and Henikoff, S. (2002). Centromeric localization and adaptive evolution of an Arabidopsis histone H3 variant. Plant Cell **14**:1053–1066.

Tomanov, K., Julian, J., Ziba, I., and Bachmair, A. (2022). SUMO Conjugation and SUMO Chain Formation by Plant Enzymes. In Plant Proteostasis: Methods and Protocols (Springer), pp. 83–92.

van den Berg, S.J.W., East, S., Mitra, S., and Jansen, L.E.T. (2023). p97/VCP drives turnover of SUMOylated centromeric CCAN proteins and CENP-A. Mol. Biol. Cell **34**:br6.

van den Berg, S.J.W., and Jansen, L.E.T. (2023). SUMO control of centromere homeostasis. Front. Cell Dev. Biol. **11**:1193192.

Walter, M., Chaban, C., Schütze, K., Batistic, O., Weckermann, K., Näke, C., Blazevic, D., Grefen, C., Schumacher, K., Oecking, C., et al. (2004). Visualization of protein interactions in living plant cells using bimolecular fluorescence complementation. Plant J. **40**:428–438.

Watanabe, R., Hara, M., Okumura, E.-i., Hervé, S., Fachinetti, D., Ariyoshi, M., and Fukagawa, T. (2019). CDK1-mediated CENP-C phosphorylation modulates CENP-A binding and mitotic kinetochore localization. J. Cell Biol. **218**:4042–4062.

Weisshart, K., Fuchs, J., and Schubert, V. (2016). Structured illumination microscopy (SIM) and photoactivated localization microscopy (PALM) to analyze the abundance and distribution of RNA polymerase II molecules on flow-sorted Arabidopsis nuclei. Bio-protocol **6**:e1725.

Wilson, V.G., and Rangasamy, D. (2001). Intracellular targeting of proteins by sumoylation. Exp. Cell Res. **271**:57–65.

Yadala, R., Ratnikava, M., and Lermontova, I. (2022). Bimolecular Fluorescence Complementation to Test for Protein–Protein Interactions and to Uncover Regulatory Mechanisms During Gametogenesis. In Plant Gametogenesis: Methods and Protocols (Springer), pp. 107–120.

Yalagapati, S.P., Ahmadli, U., Sinha, A., Kalidass, M., Dabravolski, S., Zuo, S., Yadala, R., Rutten, T., Talbert, P., Berr, A., et al. (2024). Centromeric localization of αKNL2 and CENP-C proteins in plants depends on their centromere-targeting domain and DNA-binding regions. Nucleic Acids Res. **53**:gkae1242.

Yang, F., Hu, L., Chen, C., Yu, J., O'Connell, C.B., Khodjakov, A., Pagano, M., and Dai, W. (2012). BubR1 is modified by sumoylation during mitotic progression. J. Biol. Chem. **287**:4875–4882.

Zhang, D., Martyniuk, C.J., and Trudeau, V.L. (2006). SANTA domain: a novel conserved protein module in Eukaryota with potential involvement in chromatin regulation. Bioinformatics **22**:2459–2462.

Zhang, X.-D., Goeres, J., Zhang, H., Yen, T.J., Porter, A.C.G., and Matunis, M.J. (2008). SUMO-2/3 modification and binding regulate the association of CENP-E with kinetochores and progression through mitosis. Mol. Cell **29**:729–741.

Plant Communications, Volume 7

Supplemental information

The C-terminal SUMOylation-dependent regulation of αKNL2 governs its centromere targeting and interaction with CENH3

Manikandan Kalidass, Jitka Vaculíková, Jothipriya Ramakrishnan Chandra, Barbora Králová, Venkata Ganesh Jarubula, Sevim D. Kara Öztürk, Dmitri Demidov, Veit Schubert, David Potesil, Jan J. Palecek, and Inna Lermontova

Supplemental information

The C-terminal SUMOylation-dependent regulation of αKNL2 governs its centromere targeting and interaction with CENH3

Manikandan Kalidass[1*], Jitka Vaculíková[2], Jothipriya Ramakrishnan Chandra[1], Barbora Králová[2], Venkata Ganesh Jarubula[1,3], Sevim D. Kara Öztürk[4], Dmitri Demidov[1], Veit Schubert[1], David Potesil[5], Jan J. Palecek[2,5], Inna Lermontova[1*]

[1]Leibniz Institute of Plant Genetics and Crop Plant Research (IPK) Gatersleben, Corrensstrasse 3, D-06466 Seeland, Germany

[2]National Centre for Biomolecular Research, Faculty of Science, Masaryk University, Kamenice 5, 62500 Brno, Czech Republic

[3]Department of Program Center Metacom, Leibniz Institute of Plant Biochemistry, Weinberg 3, D-06120 Halle (Saale), Germany

[4]Department of Agricultural Genetic Engineering, Ayhan Şahenk Faculty of Agricultural Sciences and Technologies, Niğde Ömer Halisdemir University, 51240, Niğde, Türkiye

[5]Central European Institute of Technology (CEITEC), Masaryk University, Kamenice 5, 62500 Brno, Czech Republic

*Authors for correspondence: kalidass@ipk-gatersleben.de; lermonto@ipk.gatersleben.de

The supplemental file includes 12 Supplemental Figures and 1 Supplemental Table

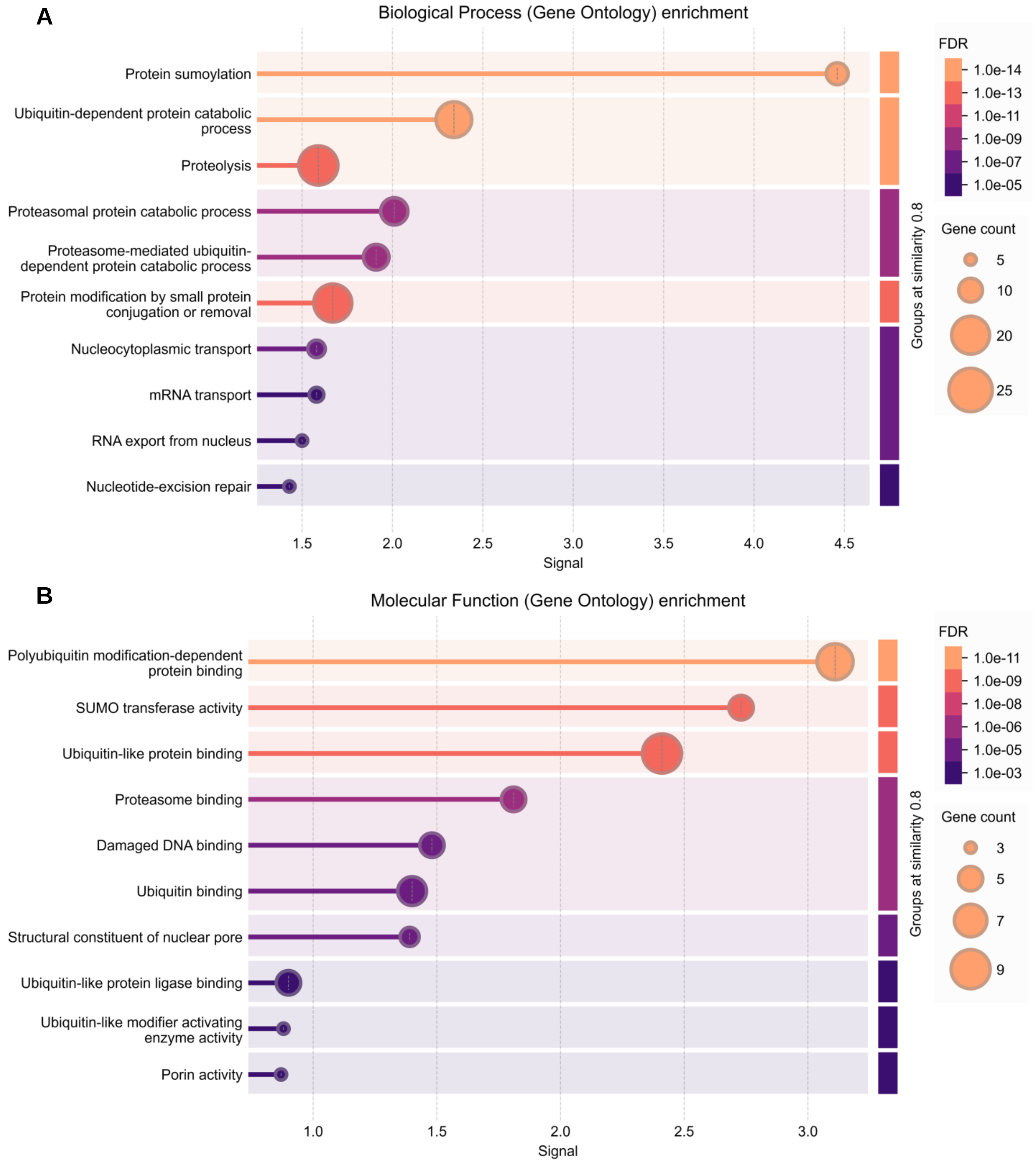

Supplementary Figure 1. The post-translational modification pathway analysis of αKNL2 interactors based on Y2H screening

(A, B) The gene ontology analysis such as biological process **(A)** and molecular function **(B)** of post-translational modification of αKNL2 interactors identified proteolysis, SUMOylation and transport pathways.

A

VENUS-N \ VENUS-C	SUMO1	SUMO2	SUMO3	SUMO5	ULP1d	ULP1d-C	ULP1d-N	Empty
αKNL2								
αKNL2-C			Nucleolus		Nucleolus	Nucleolus		
αKNL2-N								
Empty								

Positive interaction   No interaction

B

VENUS-N \ VENUS-C	αKNL2	αKNL2-C	αKNL2-N	Empty
SUMO1				
SUMO2				
SUMO3		Nucleolus		
SUMO5				
ULP1d		Nucleolus		
ULP1d-C		Nucleolus		
ULP1d-N				
Empty				

Positive interaction   No interaction

Supplementary Figure 2. The interaction analysis of SUMO and ULP1d proteins with αKNL2 by BiFC

The BiFC interactions shown for the combinations such as αKNL2, αKNL2-N, αKNL2-C fused to VENn and SUMO1, SUMO2, SUMO3, SUMO5, ULP1d, ULP1d-N or ULP1d-C fused to VENc **(A)** and vice versa **(B)**. SUMO3 and ULP1d showed interaction only with αKNL2-C, while other SUMO proteins did not interact with any αKNL2 variants. The empty BiFC negative controls were used to validate the positive interactions.

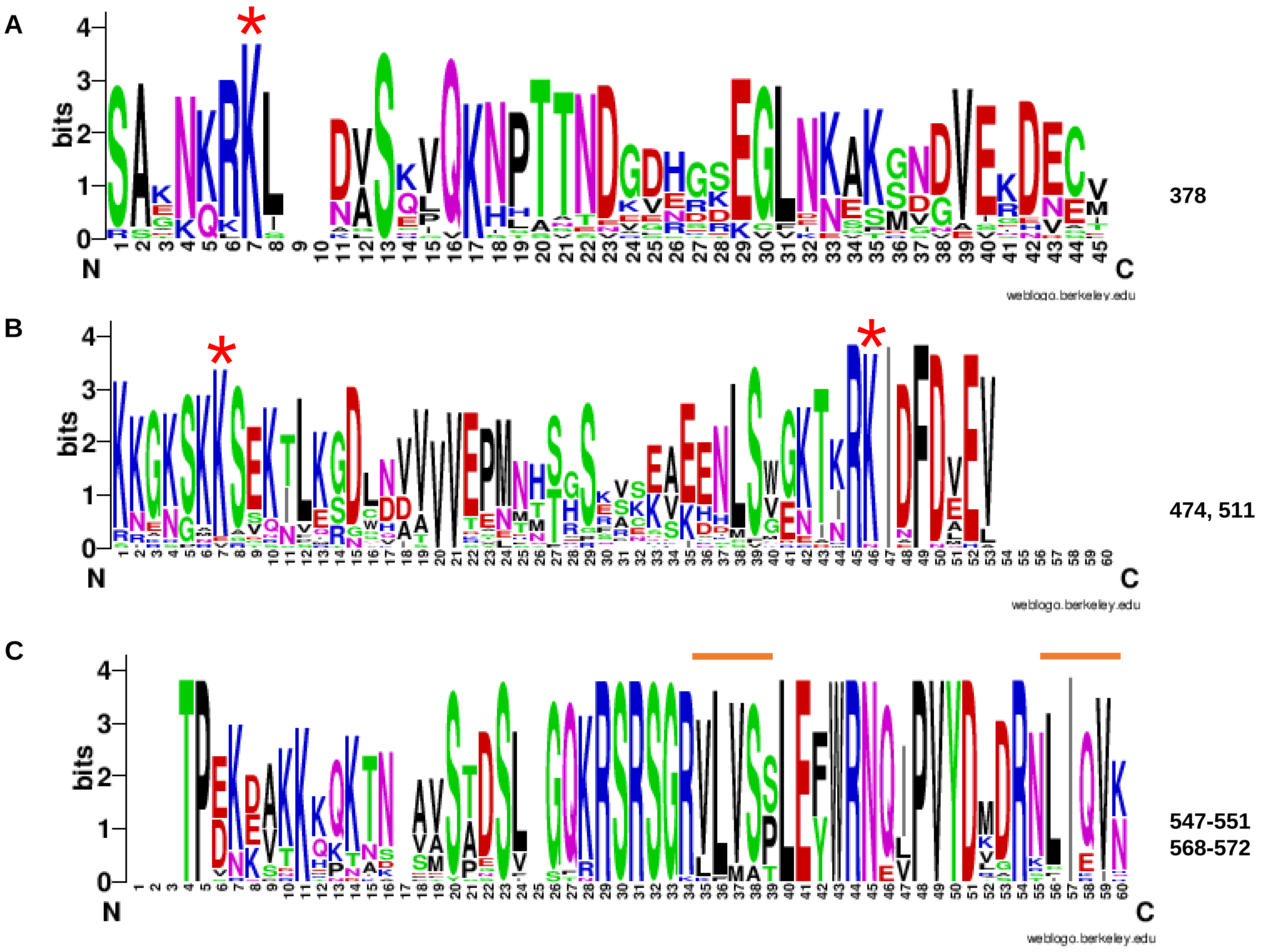

Supplementary Figure 3. The conservation analysis of SUMOylation and SIM sites in αKNL2-C

(A–C) The conservation of SUMOylation and SIM sites predicted by GPS-SUMO in αKNL2-C across Brassicales species, as illustrated using WebLogo (https://weblogo.berkeley.edu/logo.cgi). Conserved lysine residues and SUMO interaction sites are indicated by red asterisks and orange lines, respectively.

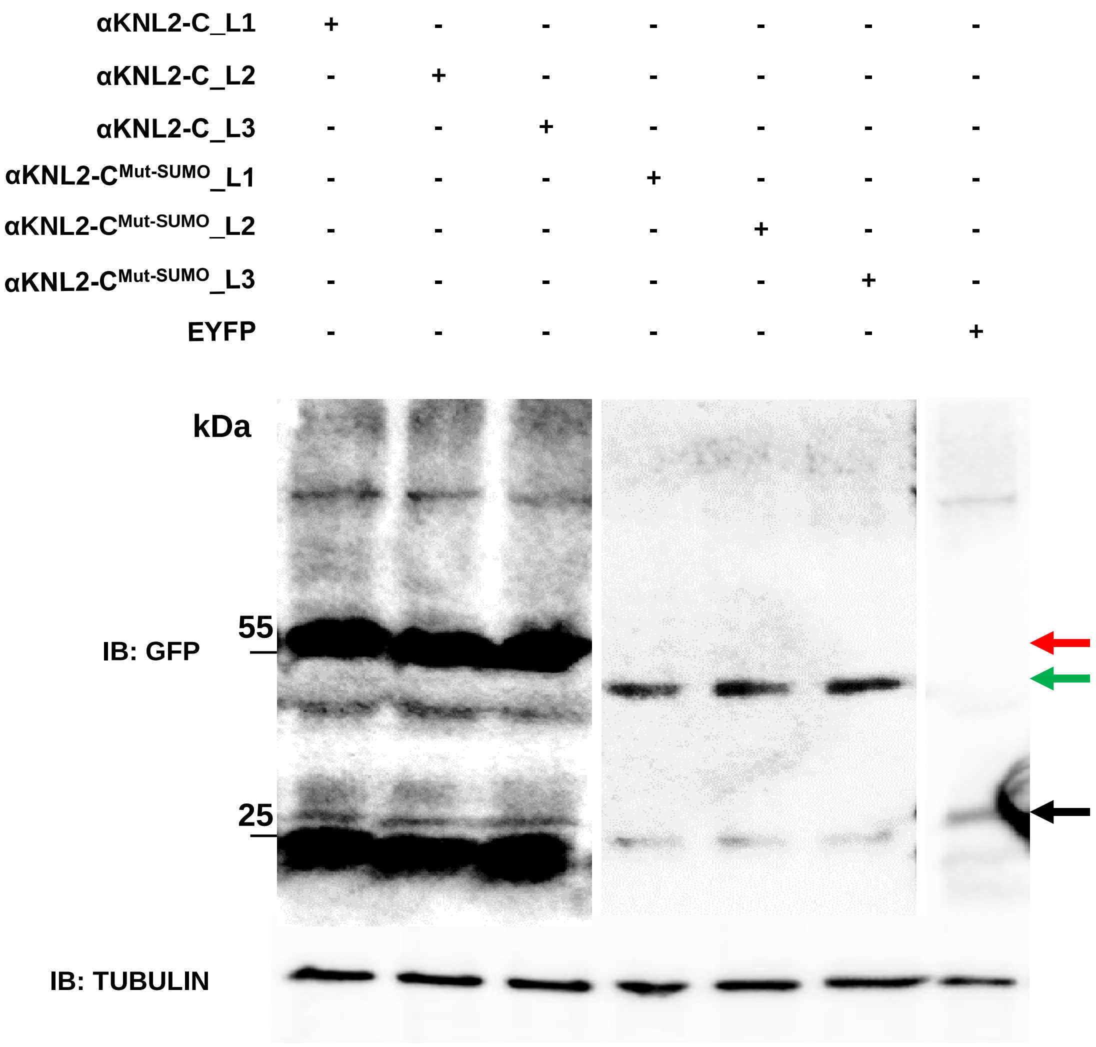

Supplementary Figure 4. Immunoblot detection of αKNL2-C-EYFP and SUMOylation-deficient αKNL2-C$^{Mut\text{-}SUMO}$-EYFP in Arabidopsis transgenic lines

Total protein extracts from three independent transgenic lines expressing αKNL2-C-EYFP, SUMOylation-deficient mutant αKNL2-C$^{Mut\text{-}SUMO}$-EYFP or EYFP alone were subjected to GFP immunoblotting. The red arrow, green and black indicates the expected size of the αKNL2-C-EYFP, αKNL2-C$^{Mut\text{-}SUMO}$-EYFP and EYFP fusion protein, respectively. The tubulin was used as a loading control to confirm equal protein loading. IB, Immunoblot.

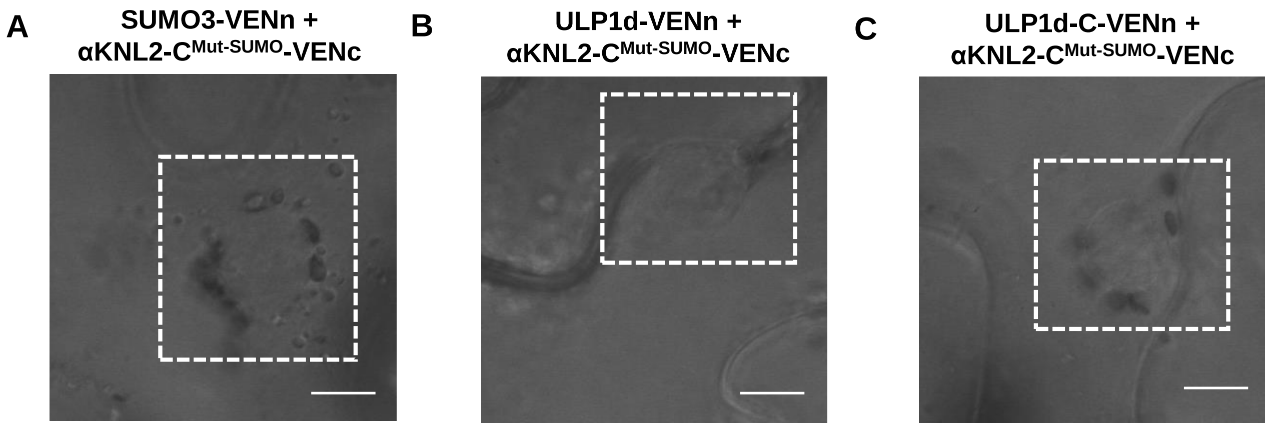

D

		VENUS-C		
	**Proteins**	**SUMO3**	**ULP1d**	**ULP1d-C**
VENUS-N	**αKNL2**			
VENUS-N	**αKNL2-C**	Nucleolus	Nucleolus	Nucleolus
VENUS-N	**αKNL2-N**			
VENUS-N	**αKNL2-C$^{Mut\text{-}SUMO}$**			

Positive interaction   No interaction   No interaction after mutagenesis

E

		VENUS-C		
	**Proteins**	**SUMO3**	**ULP1d**	**ULP1d-C**
VENUS-N	**K378R--αKNL2-C**	Nucleolus	Nucleolus	Nucleolus
VENUS-N	**K474R--αKNL2-C**	Nucleolus	Nucleolus	Nucleolus
VENUS-N	**K511R--αKNL2-C**	Nucleolus	Nucleolus	Nucleolus
VENUS-N	**Δ547-551--αKNL2-C**	Nucleolus	Nucleolus	Nucleolus
VENUS-N	**Δ568-572--αKNL2-C**	Nucleolus	Nucleolus	Nucleolus
VENUS-N	**K378R-K474R--αKNL2-C**	Nucleolus	Nucleolus	Nucleolus
VENUS-N	**K474R-K511R--αKNL2-C**			

Positive interaction   No interaction   No interaction after mutagenesis

Supplementary Figure 5. The interaction of SUMO3 and ULP1d with SUMO mutant variants of αKNL2-C by BiFC
(A-C) No interaction was found between SUMO3, ULP1d, ULP1d-C fused to VENn with αKNL2-C$^{Mut\text{-}SUMO}$ fused to VENc. The nucleus lacking Venus fluorescence is indicated by white dotted circles. Scale bars represents 5 µm. **(D)** The similar interaction results was found when the orientation of the Venus fusion was reversed. The interaction was indicated in a colour code below the table. **(E)** Analysis of the interactions between individual SUMOylation and SIM sites of αKNL2-C with SUMO3, ULP1d, or ULP1d-C. Double lysine mutations (K474R and K511R) abolished interactions with SUMO3 and ULP1d. Interaction outcomes are indicated by a color code below the table.

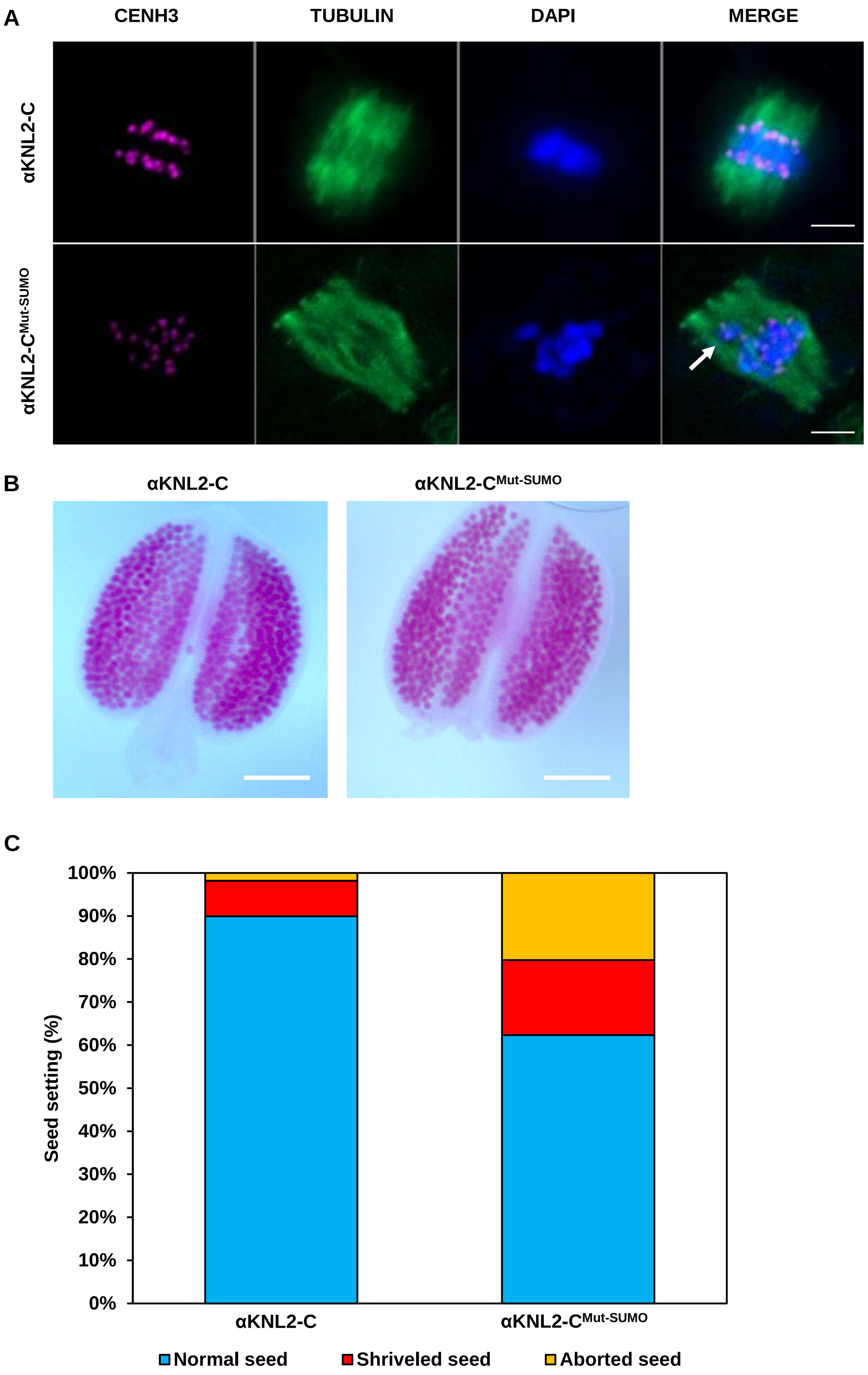

Supplementary Figure 6. Analysis of chromosome segregation defects, pollen viability, and seed set in the SUMOylation-deficient αKNL2 mutant

(A) Representative images of mitotic chromosome segregation in Arabidopsis αKNL2 SUMO mutant plants. Microtubules (green) are stained with anti-tubulin, centromeres (magenta) with anti-CENH3, and DNA (blue) with DAPI. The upper row shows normal metaphase alignment, while the lower panel depicts a misaligned chromosome (arrow) during metaphase. Scale bars 5 µm. **(B)** Alexander staining of pollen grains from αKNL2-C (left) and αKNL2$^{Mut\text{-}SUMO}$ mutant (right) plants. The mutant anthers did not show any difference compared to αKNL2-C control plants, suggesting no defects in pollen viability. Scale bars 10 µm. **(C)** Analysis of seed setting in *Arabidopsis* αKNL2-C or αKNL2-C$^{Mut\text{-}SUMO}$ mutant variant. Bar graph showing the number of normal, shriveled and aborted seeds per silique for 10 plants per construct, with 10 siliques analyzed per plant.

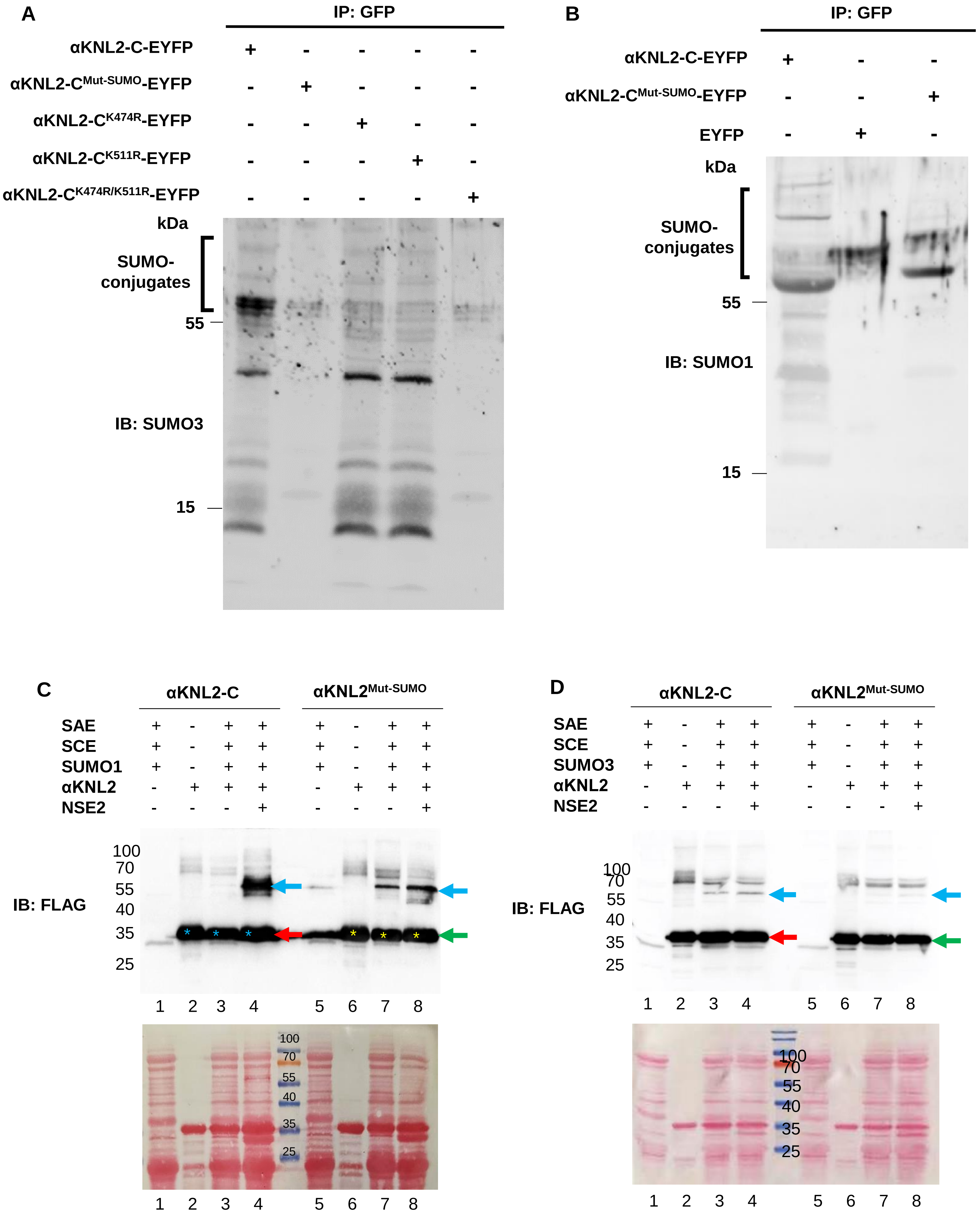

A
IP: GFP
αKNL2-C-EYFP
αKNL2-C^Mut-SUMO-EYFP
αKNL2-C^K474R-EYFP
αKNL2-C^K511R-EYFP
αKNL2-C^K474R/K511R-EYFP
kDa
SUMO-conjugates
55
15
IB: SUMO3
B
IP: GFP
αKNL2-C-EYFP
αKNL2-C^Mut-SUMO-EYFP
EYFP
kDa
SUMO-conjugates
55
15
IB: SUMO1
C
αKNL2-C
αKNL2^Mut-SUMO
SAE
SCE
SUMO1
αKNL2
NSE2
100
70
55
40
35
25
IB: FLAG
1 2 3 4 5 6 7 8
D
αKNL2-C
αKNL2^Mut-SUMO
SAE
SCE
SUMO3
αKNL2
NSE2
100
70
55
40
35
25
IB: FLAG
1 2 3 4 5 6 7 8

Supplementary Figure 7. The in vivo and in vitro SUMOylation analysis of αKNL2-C

(A, B) In vivo SUMOylation of αKNL2 by SUMO3 or SUMO1 in *Nicotiana benthamiana*. **(A)** Leaves expressing αKNL2-C-EYFP, αKNL2-C$^{Mut\text{-}SUMO}$-EYFP, αKNL2-C^{K474R}-EYFP, αKNL2-C^{K511R}-EYFP, or αKNL2-C$^{K474R/K511R}$-EYFP were tested with SUMO3. **(B)** Leaves expressing αKNL2-C-EYFP, αKNL2-C$^{Mut\text{-}SUMO}$-EYFP, or EYFP alone were tested with SUMO1. Total protein extracts were immunoprecipitated with GFP-Trap beads and probed with anti-SUMO3 or anti-SUMO1 antibodies. SUMO conjugates are marked by black brackets. IB, immunoblot; IP, immunoprecipitation. **(C, D)** The in vitro SUMOylation assay was performed to assess the SUMOylation efficiency of αKNL2-C and its SUMO mutant variant using the SUMO1 (**C**) or SUMO3 (**D**) isoforms. The reactions included enzymes only (lanes 1 and 5), substrate only (lanes 2 and 6), a mixture of enzymes and substrate (lanes 3 and 7), and a complete reaction with the addition of NSE2 SUMO-E3 ligase (lanes 4 and 8). Following incubation, samples were analyzed using SDS-PAGE and immunoblotting. The membrane was reversibly stained with Ponceau S red (lower panels) as a loading control to verify equal protein loading across all reactions. Both αKNL2 variants and their SUMOylated forms were detected using an anti-FLAG antibody (upper panels). The red arrows represents the unmodified αKNL2-C, the green arrows marks the unmodified αKNL2-C$^{Mut\text{-}SUMO}$ mutant, and the blue arrows indicate SUMOylated forms. The SUMO mutant shows a significantly reduced SUMOylation efficiency compared to the wild-type variant. Notably, the addition of NSE2 did not enhance SUMO3 efficiency for either αKNL2 variant, while it enhanced SUMO1 efficiency for both variants. IB, immunoblot; IP, immunoprecipitation.

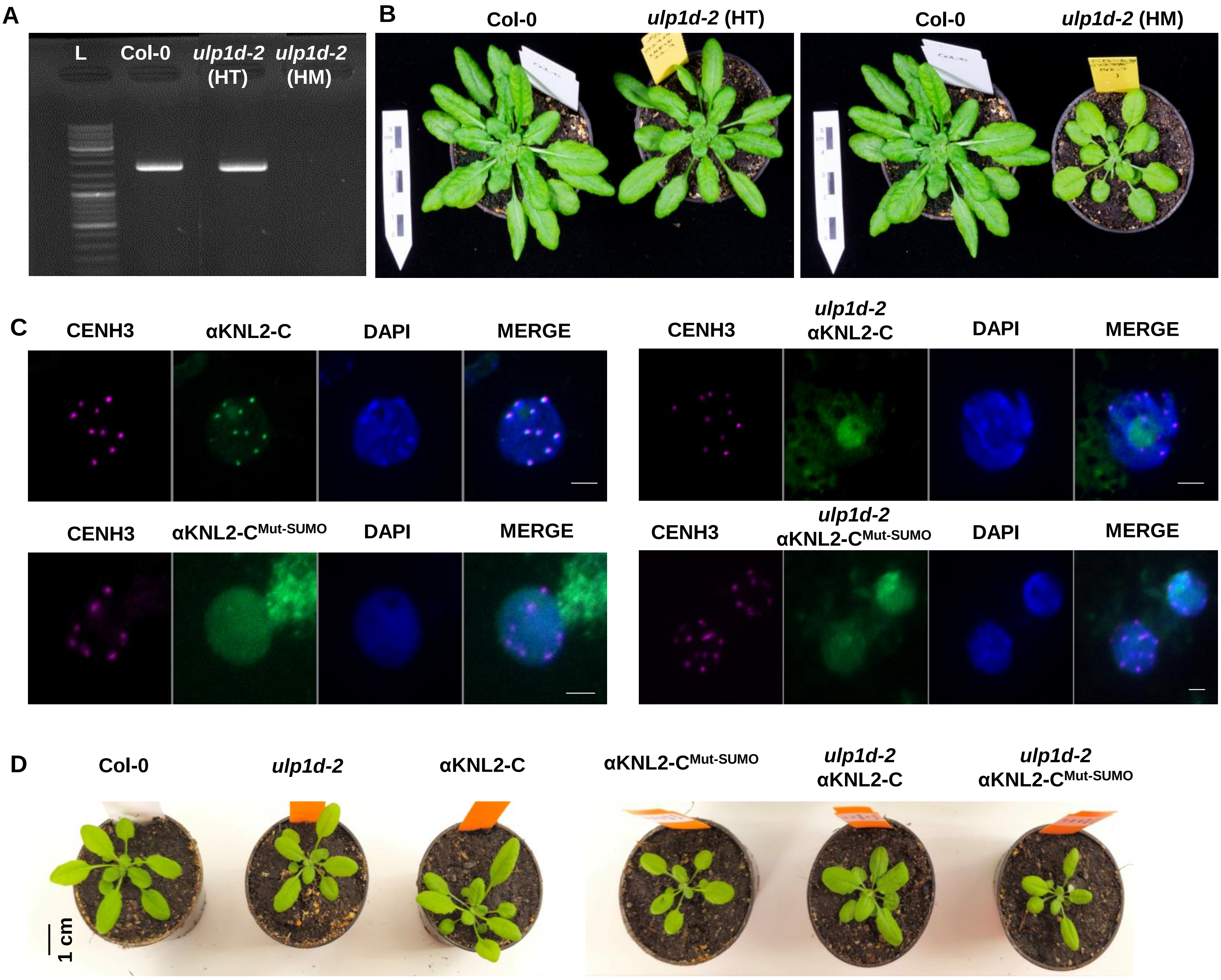

Supplementary Figure 8. The phenotype characteristics and localization of αKNL2-C and αKNL2-C$^{\text{Mut-SUMO}}$ in *ulp1d-2*
(A-B) RT-PCR analysis of ULP1d expression (A) and the phenotype of 5 weeks grown plants (B) of wild-type (Col-0), *ulp1d-2* heterozygous and homozygous backgrounds. **(C)** Immunostaining experiments showing the co-localization of αKNL2-C and KNL2-C$^{\text{Mut-SUMO}}$ (green) in meristematic nuclei of wild-type and *ulp1d-2* mutants. The nuclei were stained with anti-CENH3 (red) and DAPI was used as a counterstain. Scale bars represents 5 µm. **(D)** The phenotype of the αKNL2-C and KNL2-C$^{\text{Mut-SUMO}}$ in wild-type and *ulp1d-2* mutants grown for 4 weeks in soil. Scale bar represents 1 cm.

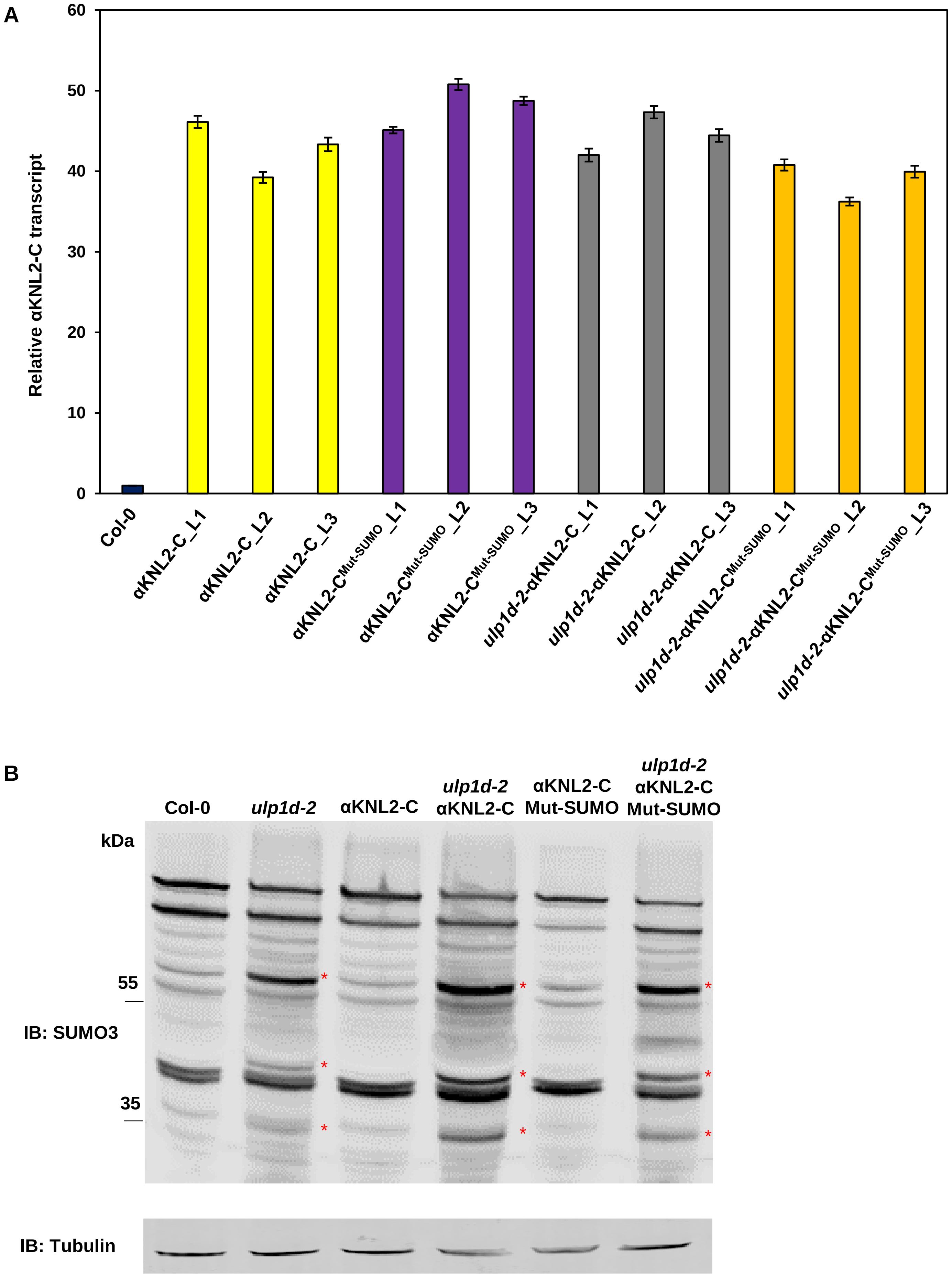

Supplementary Figure 9. Transcript levels of *αKNL2* and SUMO3 western blot in αKNL2-C and αKNL2-C$^{\text{Mut-SUMO}}$ lines in wild-type and *ulp1d-2* mutant plants

(A) Quantitative real-time PCR (RT-qPCR) analysis of αKNL2 transcripts in αKNL2-C and SUMOylation-deficient αKNL2-C$^{\text{Mut-SUMO}}$ independent Arabidopsis transgenic lines. Wild-type (Col-0) were included as controls. Transcript levels were normalized to *ACTIN2 and UBQ* expression. Similar transcript levels were detected across αKNL2-C and αKNL2-C$^{\text{Mut-SUMO}}$ transgenic lines in Col-0 and *ulp1d-2.* Data represent as mean ± SEM. Statistical analysis by ANOVA revealed no significant differences between the lines ($p > 0.5$). **(B)** Western blot analysis against anti-SUMO3 in the total protein extracts from wild-type, *ulp1d-2,* αKNL2-C, and αKNL2-C$^{\text{Mut-SUMO}}$ in wild-type and *ulp1d-2* mutants. The red asterisks shows the increase in the band intensities in *ulp1d-2* mutant background compared to wild-type (Col-0). IB, Immunoblot.

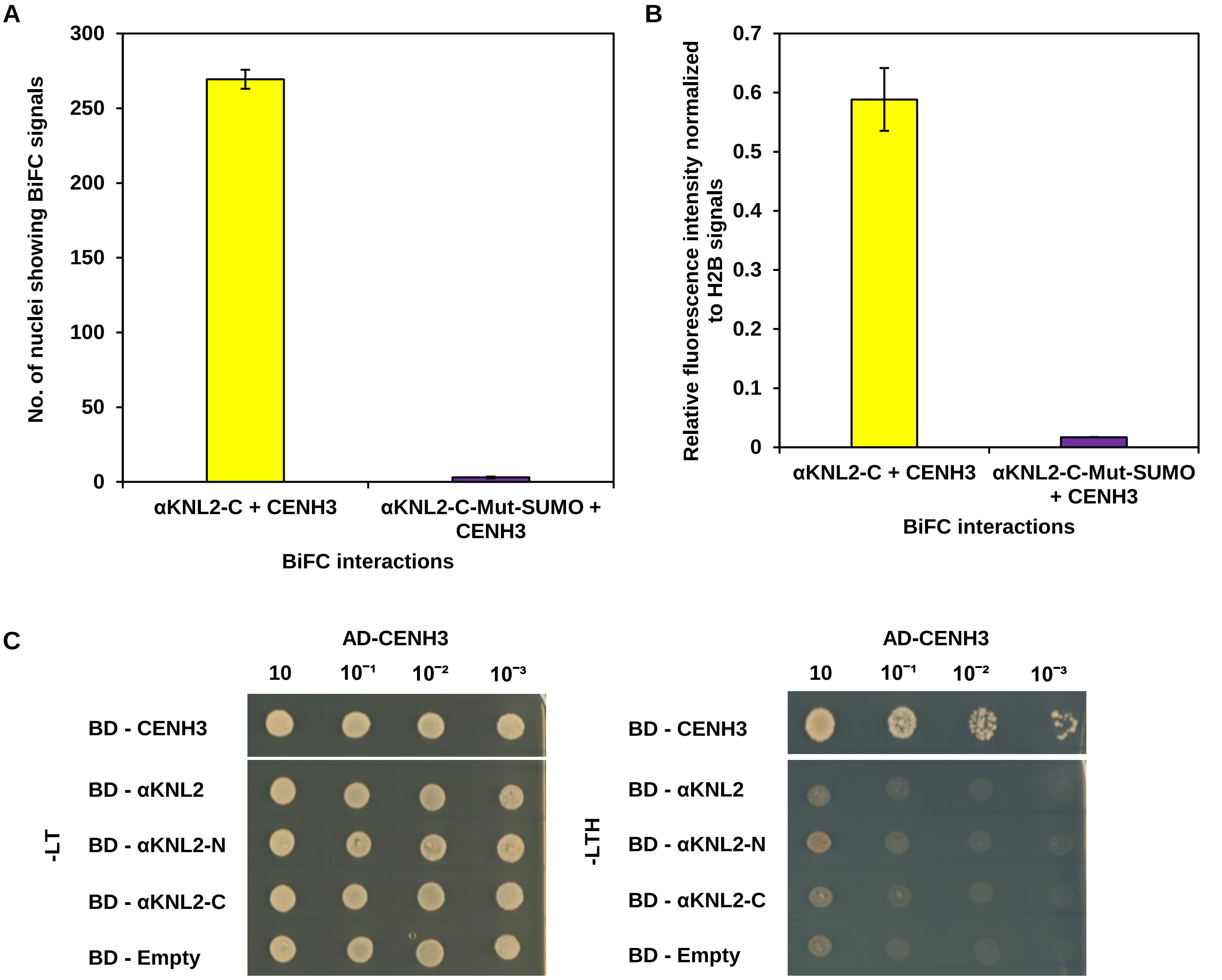

Supplemental Figure 10. BiFC quantification and yeast two-hybrid assay for interactions between αKNL2 and CENH3 **(A)** Bar graphs represent the number of nuclei showing BiFC signals for αKNL2, αKNL2$^{Mut\text{-}SUMO}$ and CENH3 interactions. The number of nuclei showing BiFC signals was measured in 80mm^2 area. Data are presented as mean ± SEM. **(B)** The fluorescence intensity for BiFC signals were measured after normalization with H2B signals from 30 nuclei per sample (n = 30). Data are presented as mean ± SEM. **(C)** Zygotes expressing both prey CENH3 and bait (CENH3, αKNL2, αKNL2-N, αKNL2-C) are selected on -LT (Double dropout: YNB without Leu and Trp). Protein-protein interactions are assessed on -LTH (Triple dropout: YNB without Leu, Trp, and His). The strength of the protein-protein interactions was evaluated by a drop dilution test. AD, activating domain; BD, binding domain.

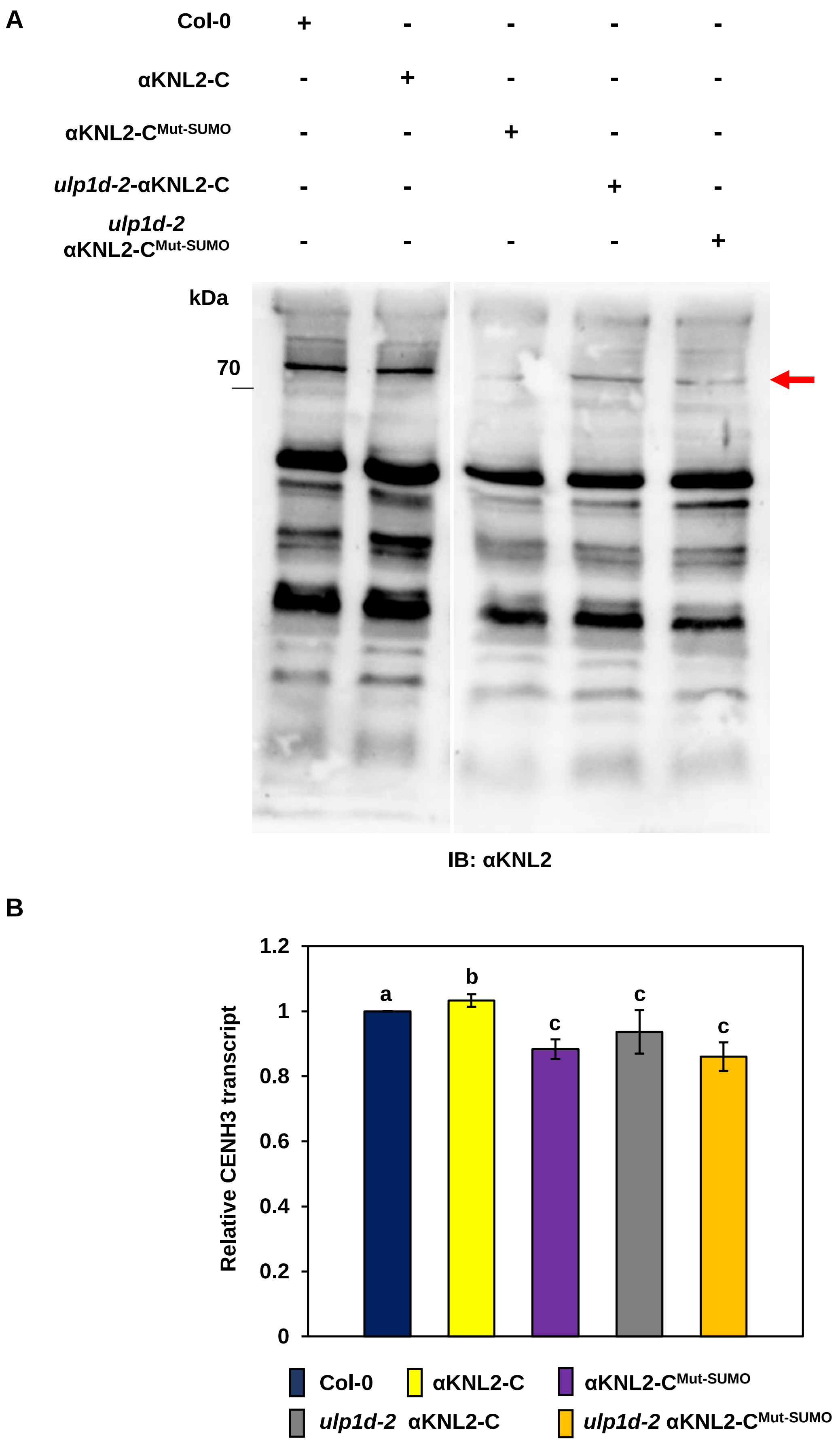

Supplementary Figure 11. αKNL2 protein and *CENH3* transcript levels in αKNL2-C and αKNL2-C$^{Mut\text{-}SUMO}$ lines in wild-type and *ulp1d-2* mutant plants

(A) Immunoblot analysis of αKNL2 in wild-type (Col-0), αKNL2-C-EYFP, and αKNL2-C$^{Mut\text{-}SUMO}$-EYFP in Col-0 and *ulp1d-2* background lines. The nuclear protein extracts were separated by SDS-PAGE and probed with anti-αKNL2 antibodies to detect endogenous αKNL2. The red arrow indicates the αKNL2 specific band. The tubulin control is same as Fig. 7C. **(B)** Quantification of *CENH3* transcript levels by RT-qPCR. Data are normalized to *ACTIN2, UBQ* expression and shown as mean ± SEM from three biological replicates. Significant differences are marked by lowercase letters based on ANOVA and Tukey's multiple comparison tests ($P < 0.005$).

αKNL2 localization pattern

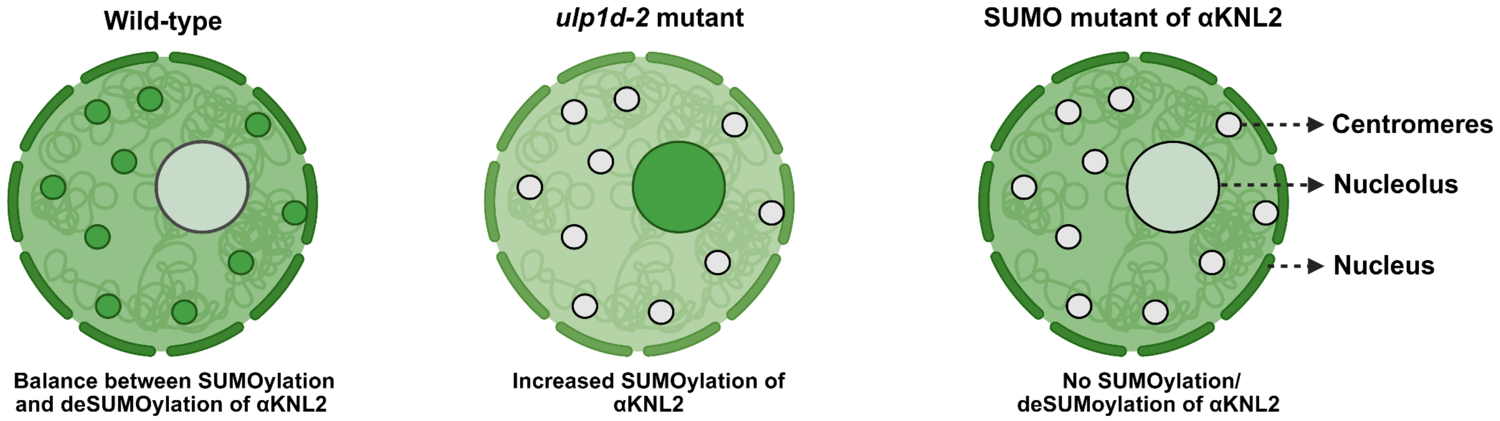

Supplementary Figure 12. SUMOylation-dependent regulation of αKNL2 localization and its impact on centromere function

In wild-type cells, αKNL2 (green) predominantly localizes to the centromere region and facilitates proper CENH3 deposition. In *ulp1d* mutants, αKNL2 localization is restricted to the nucleolus and is absent from the centromeres. In SUMOylation-deficient αKNL2 mutants, αKNL2-C fails to localize correctly and is entirely mislocalized to the cytoplasm. Nucleoplasmic signals were observed across all cases, with occasional weak nucleolar staining detected in both wild-type and SUMO mutant backgrounds.

Supplementary Table 1. Primers used in this study

S. No	Gene	Forward primer	Reverse primer
Amplification of genes using attB primers			
1	SUMO1 (AT4G26840)	GGGGACAAGTTTGTACAAAAAAGCAGGCTTCATGTCTGCAAACCAGGAGG	GGGGACCACTTTGTACAAGAAAGCTGGGTGGCCGTAGCACCACCACCGC
2	SUMO2 (AT5G55160)	GGGGACAAGTTTGTACAAAAAAGCAGGCTTCATGTCTGCTACTCCGGAAGA	GGGGACCACTTTGTACAAGAAAGCTGGGTAAAGCAGAAGAGCTTCAGGC
3	SUMO3 (AT5G55170)	GGGGACAAGTTTGTACAAAAAAGCAGGCTTCATGTCTAACCCTCAAGATGA	GGGGACCACTTTGTACAAGAAAGCTGGGTAAGCCCATTATGATCGAAAAG
4	SUMO5 (AT2G32765)	GGGGACAAGTTTGTACAAAAAAGCAGGCTTCATGGTGAGTTCCACAGACAC	GGGGACCACTTTGTACAAGAAAGCTGGGTAGGAGTGTAAGGACCGCCACC
5	ULP1d (AT1G60220)	GGGGACAAGTTTGTACAAAAAAGCAGGCTTCATGACGAAGAGGAAGAAGG	GGGGACCACTTTGTACAAGAAAGCTGGGTTTACTCTGTCTGGTCACTGAC
6	ULP1d-N (AT1G60220)	GGGGACAAGTTTGTACAAAAAAGCAGGCTTCATGACGAAGAGGAAGAAGG	GGGGACCACTTTGTACAAGAAAGCTGGGTCTTACGGCGCCTTGAACTTTG
7	ULP1d-C (AT1G60220)	GGGGACAAGTTTGTACAAAAAAGCAGGCTTCATGAAATCAGAGGACACAGTG	GGGGACCACTTTGTACAAGAAAGCTGGGTCTCTGTCTGGTCACTGACACG
Primers used to confirm the positive entry and destination clones			
8	attB1	GGGGACAAGTTTGTACAAAAAAGCAGGCTTC	
9	attB2	GGGGACCACTTTGTACAAGAAAGCTGGGTC	
Primers used for PCR-based site-directed mutagenesis			
10	K378R-αKNL2C	AAACAAAAGGAGAATCGATGCGAG	TCCGCACTTTTGACTTTCGTCCCAG
11	K474R-αKNL2C	GAAAATCAAAGAGAAGTGAGAAGA	CTTTCGACAGGGGATCTTGAAATGC
12	K511R-αKNL2C	AATAAAGAGGAGAATCGACTTTG	TTTTCCCATGACAAGTTTTCTTCAG
13	Δ547-551-αKNL2C	CTAGAGTTTTGGCGTAACCAAATTC	CCTTCCTGATCTTGACCGTTTCTGT
14	Δ568-572-αKNL2C	GATGGTAGTGAGACTAACTCCGCTC	GTTCCGATCCATATCATAAACAGG
Primers used for cloning constructs for in vitro SUMOylation assay			
15	αKNL2 C-terminus (WT and SUMO mutant) to pET-Duet ; JJ225, JJ226	ACCATCATCACCACAGCCAGATGAATTACTCTGGGACG	CTGAAAATACAGGTTTTCCGCTTTGATTTTCAAGTTTCTTCG
16	NSE2 to pET28 c+ ; JJ200, JJ201	GGTGGACAGCAAATGGGTCGGATCCCCATGGCGTCGGCGTCCTCG	GGTGGTGGTGGTGGTGCTCGAGCTAATCTTCATCCACATCTTCTGTGAA
**Primers used for RT-qPCR analysis**			
17	qαKNL2-C	TCGACTTTGATGTGGAGGTAACAC	GAATCAGTAGACGCCGCATTGG
18	qCENH3	GCAGGTCCAACTACGACCC	GCTGGTGAAGTTGTAGGATTTGT
